# Palladium-catalyzed hydroalkylation of methylenecyclopropanes with simple hydrazones†

**DOI:** 10.1039/d0sc01221a

**Published:** 2020-05-15

**Authors:** Jinzhong Yao, Zhangpei Chen, Lin Yu, Leiyang Lv, Dawei Cao, Chao-Jun Li

**Affiliations:** Department of Chemistry, FQRNT Centre for Green Chemistry and Catalysis, McGill University 801 Sherbrooke St. W. Montreal Quebec H3A 0B8 Canada cj.li@mcgill.ca; College of Biological, Chemical Sciences and Engineering, Jiaxing University Jiaxing 314001 People's Republic of China

## Abstract

A palladium-catalyzed hydroalkylation reaction of methylenecyclopropanes *via* highly selective C–C σ-bond scission was achieved under mild conditions, in which simple hydrazones served as carbanion equivalents. This method featured good functional group compatibility, affording high yields of C-alkylated terminal alkenes.

## Abstract 2

A palladium-catalyzed hydroalkylation of methylenecyclopropanes *via* selective C–C σ-bond scission was achieved, in which simple hydrazones served as carbanion equivalents. This method affords high yields of C-alkylated terminal alkenes with good functional group compatibility.

## Graphical abstract

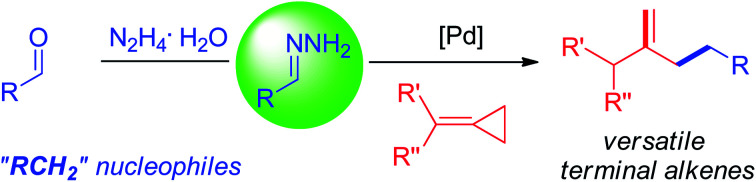

## Introduction

**Scheme 1:**
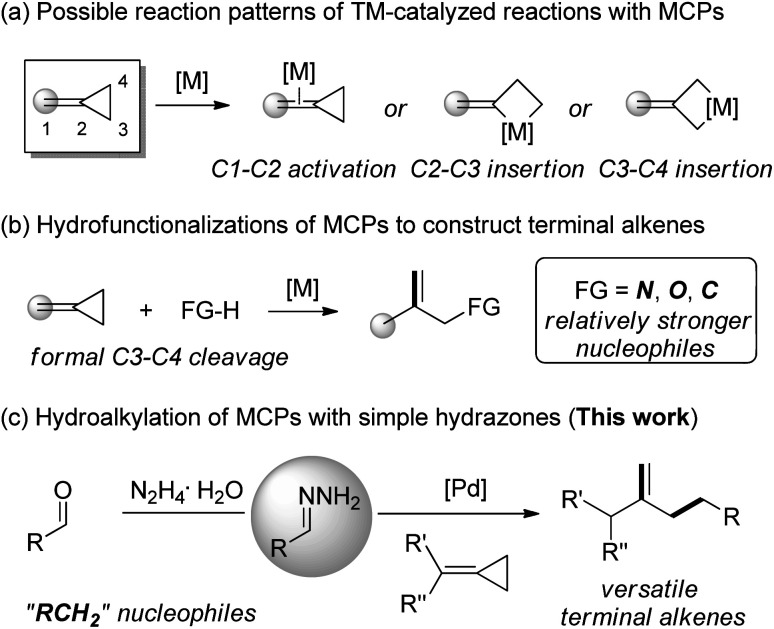
Transition-metal-catalyzed transformations of MCPs.

Transition-metal-catalyzed carbon–carbon σ-bond activation towards the reconstruction of new carbon–carbon/hetero bonds is a fundamentally challenging process in organic chemistry.^1^ Small (three- or four-membered) saturated and unsaturated carbon-rings are ideal candidates for such transformations due to the strain-release driving force.^2^ Consequently, the reactivity of methylenecyclopropanes (MCPs), a type of three-membered ring tethered by a highly strained double bond, has received much attention in organic synthesis.^3^ Possible reaction patterns of MCPs with respect to the transition metal catalysis involve the activation of the proximal *exo*-methylene double bond (C1–C2 activation) and formation of metallacyclobutane species through insertion to the distal single bonds C2–C3 or C3–C4 (Scheme 1a).^4^ Notably, the hydrofunctionalization of MCPs concerning formal C3–C4 cleavage would provide an efficient way to obtain various terminal alkenes despite confronting with the regioselectivity problem (Scheme 1b).^5,6^

On the other hand, terminal alkenes constitute important intermediates for organic synthesis.^7^ Particularly, they are not only widely used in the chemical industry for large-scale polymerisations, but also in more special reactions such as metathesis, epoxidations, hydroformylations, hydroaminations, and others.^8^ Consequently, reliable methods toward the facile generation of versatile terminal alkenes would be very desirable in organic synthesis.^9^ Toward this target, some efforts have been focused on the hydrofunctionalizations of MCPs through selective C–C distal-bond cleavage, mostly developed by the groups of Yamamoto,^10^ Shi^11^ and Mascareñas,^12^ with several types of pronucleophiles with relatively stronger nucleophilicity, such as nitrogen, oxygen and carbanion nucleophiles. As a result, a variety of functionalized terminal alkenes have been fabricated through these transformations. However, as to the more challenging alkyl substituted terminal alkenes, few successes have been achieved.^13^ Thus, the development of a modular approach toward hydroalkylation of MCPs with simple alkyl reagents is of great significance.

Recently, our group discovered that hydrazones could serve as alkyl carbanion equivalents in several cross-coupling reactions^14^ and nucleophilic additions^15^*via* polarity reversal. Inspired by our recent work on the palladium-catalyzed Tsuji–Trost reaction, in which hydrazones served as C-nucleophiles instead of traditional alkyl organometallic reagents,^16^ we wondered if these alkyl pronucleophiles are suitable for the hydroalkylation reaction of MCPs in the presence of the palladium catalyst. To achieve such a transformation would be inherently challenging due to the multiple reactivities of MCPs with the palladium catalyst and the competition between N- and C-nucleophilic attacks of hydrazones (Scheme 1c). Herein, we wish to report a palladium-catalyzed hydroalkylation reaction of MCPs using simple hydrazones as alkyl carbanion equivalents.

## Results and discussion

In the preliminary investigation, we examined the reaction of MCP **1a** with phenyl hydrazone **2a** (Table 1). The reaction of **1a** with two equivalents of **2a** in the presence of catalytic amounts of [Pd(allyl)Cl]_2_ (5 mol%) and IPr·HCl (20 mol%), and 1.2 equiv. of KOH in THF at 50 °C gave the hydroalkylation product in 80% yield (entry 1). The reaction did not occur in the absence of palladium or base (entries 2 and 3). Other palladium catalysts, such as Pd_2_(dba)_3_, showed a relatively lower efficiency (entry 4). The ligand played an important role in this transformation. An N-heterocyclic carbene (NHC) ligand like SIPr·HCl was also suitable for this reaction (entry 5), while other ligands, such as PCy_3_ and dppp, led to poor results (entries 6 and 7). Among the bases examined, NaOH exhibited a comparable reaction efficiency, while others showed poor reactivity (entries 8–12). 1,4-Dioxane was also effective as a solvent for this reaction, delivering the product with 68% yield (entry 13). The temperature was found to influence the reactivity, as both lower and higher temperatures decreased the yields (entries 14 and 15).

**Table 1:** Optimization of the reaction conditions^a^

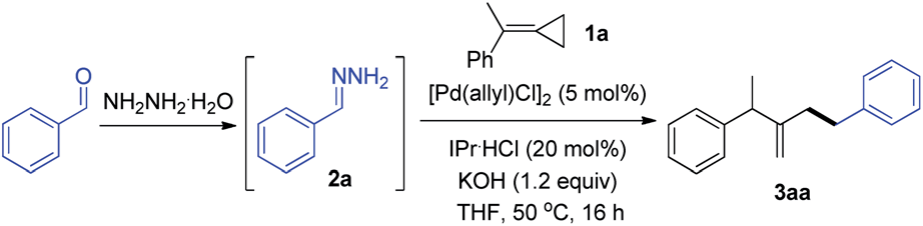		
Entry	Variation from “standard conditions”	NMR yield (%)
1	No change	80 (75)^b^
2	No [Pd(allyl)Cl]_2_	0
3	No base	0
4	Pd_2_(dba)_3_ instead of [Pd(allyl)Cl]_2_	32
5	SIPr·HCl instead of IPr·HCl	70
6	PCy_3_ instead of IPr·HCl	40
7	dppp instead of IPr·HCl	0
8	*t*-BuOLi instead of KOH	54
9	*t*-BuONa instead of KOH	45
10	*t*-BuOK instead of KOH	42
11	NaOH instead of KOH	71
12	K_3_PO_4_ instead of KOH	30
13	1,4-Dioxane instead of THF	68
14	35 °C instead of 50 °C	41
15	65 °C instead of 50 °C	60
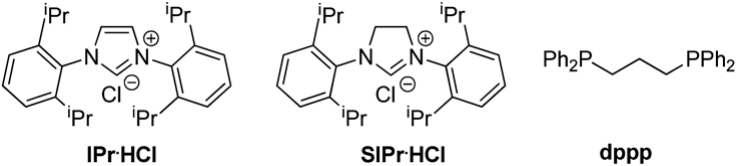		

a Reaction conditions: phenyl hydrazone **2a** (0.2 mmol, 1.0 M generated *in situ* from benzaldehyde and hydrazine), **1a** (0.1 mmol), [Pd(allyl)Cl]_2_ (5 mol%), IPr·HCl (20 mol%), and KOH (1.2 equiv.) in THF (0.2 M) were stirred under N_2_ at 50 °C for 16 h. NMR yields were given with mesitylene as the internal standard, and yields were calculated based on **1a**.

b Isolated yield was given in the parentheses.

With the optimized reaction conditions in hand, the scope and limitation of hydrazones were examined in their reaction with (1-cyclopropylideneethyl)benzene (**1a**) or (cyclopropylidenemethylene)dibenzene (**1b**) as shown in Table 2. A series of hydrazones with functional groups such as methyl, methoxy, fluoro, trifluoromethyl and *N*,*N*-dimethylamino participated in the reaction smoothly to give the products in 40–89% yields (**3aa–3ak**, **3ba**). In addition, *para*-, *meta*-, *ortho*-, and multisubstituted aromatic hydrazones were all effective in this reaction. The hydrazone generated from a polycyclic aromatic aldehyde such as 1-naphthaldehyde also led to a smooth reaction to give the desired product (**3an**) in 85% yield. Moreover, hydrazones prepared from heteroaryl aldehydes containing furan (**2l**), pyrrole (**2m**) and indole (**2o**) were also tolerated in this system (**3al**, **3am**, **3bo**). To further expand the utility of this reaction, hydrazones derived from aliphatic aldehydes were examined and found to be also effective, providing the desired products in moderate yields (**3ap**, **3bq**, **3br**).

**Table 2:** Substrate scope of hydrazones^a^

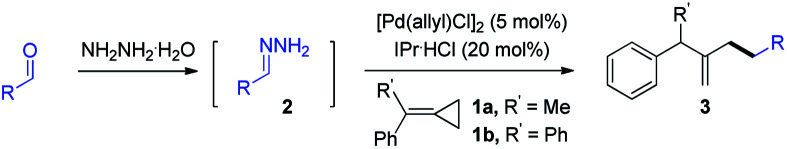
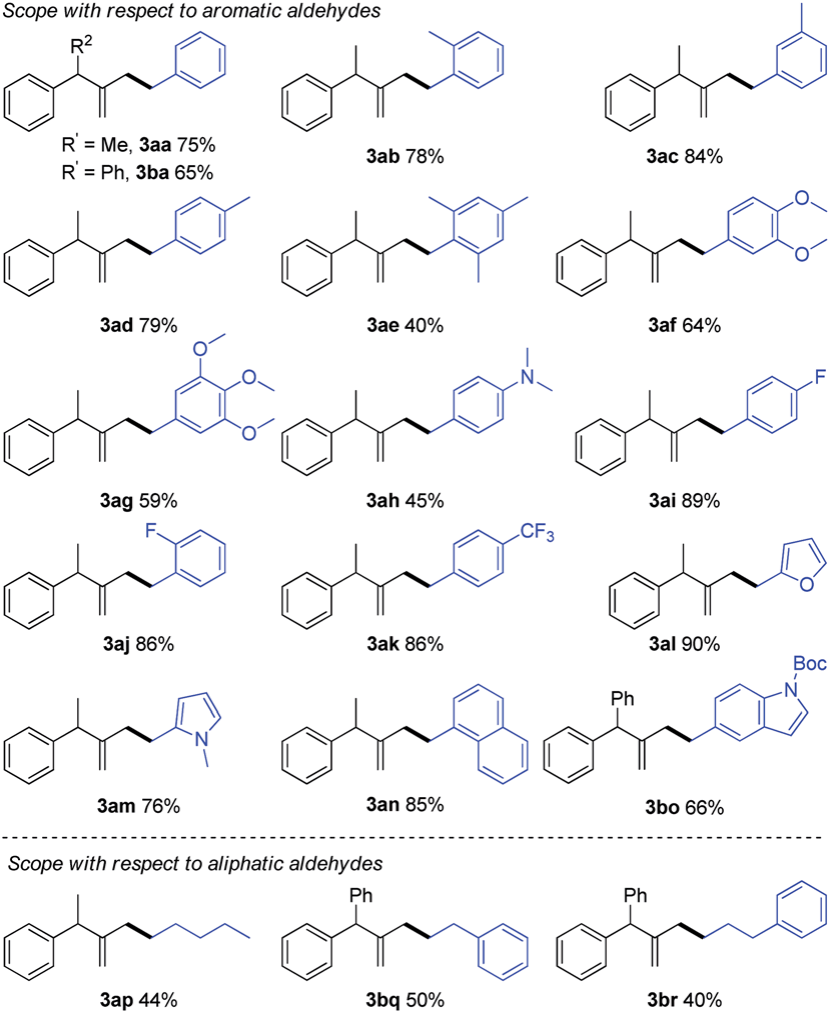

a Reaction conditions: hydrazone **2** (0.2 mmol, 1.0 M generated *in situ* from aldehyde and hydrazine), **1a** or **1b** (0.1 mmol), [Pd(allyl)Cl]_2_ (5 mol%), IPr·HCl (20 mol%), and KOH (1.2 equiv.) in THF (0.2 M) were stirred under N_2_ at 50 °C for 16 h; isolated yields were given.

Next, we evaluated the scope of the reaction with regard to the range of methylenecyclopropanes (MCPs) as shown in Table 3. In general, the reaction proceeded smoothly to give the hydroalkylation products in moderate to good yields. A variety of functional groups, including methyl, methoxy, fluoro, on the aryl ring, were compatible under the optimal reaction conditions (**3ca–3ga**). Notably, the substrate having an alkynyl group was well tolerated, and the corresponding product (**3ha**) was obtained in 62% yield. Besides the substrates with methyl and phenyl groups at the R′ position, other alkyl-substituted MCPs were all suitable for the reaction (**3ja**, **3ka**), furnishing the desired product with 66% and 81% yields, respectively. Moreover, cyclic MCPs were tolerated to provide products (**3la**, **3ma**) in good yields. However, when R′ was hydrogen, a regioisomeric mixture of **3na** and **3na′** was obtained in 60% total yield with a 3 : 2 ratio.

**Table 3:** Substrate scope of methylenecyclopropanes^a^

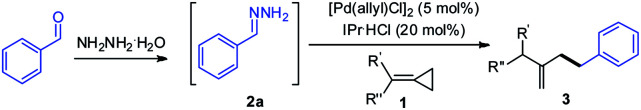
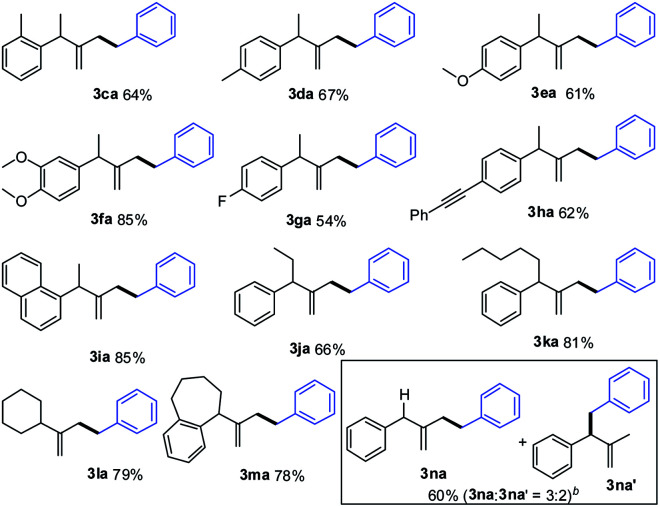

a Reaction conditions: phenyl hydrazone **2a** (0.2 mmol, 1.0 M generated *in situ* from benzaldehyde and hydrazine), **1** (0.1 mmol), [Pd(allyl)Cl]_2_ (5 mol%), IPr·HCl (20 mol%), and KOH (1.2 equiv.) in THF (0.2 M) were stirred under N_2_ at 50 °C for 16 h; isolated yields were given.

b The ratio of **3na** and **3na′** was determined by ^1^H NMR analysis of the crude mixture.

**Scheme 2:**
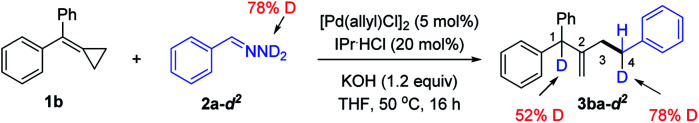
Deuterium-labelling experiment.

To gain mechanistic insight into this transformation, a preliminary D-labelling experiment was conducted (Scheme 2). When hydrazone (**2a-d2**) was reacted with **1b**, the deuterated product **3ba-d2** was obtained, in which the deuterium isotope is incorporated at the C1 (52% D) and C4 (78% D) positions.

**Scheme 3:**
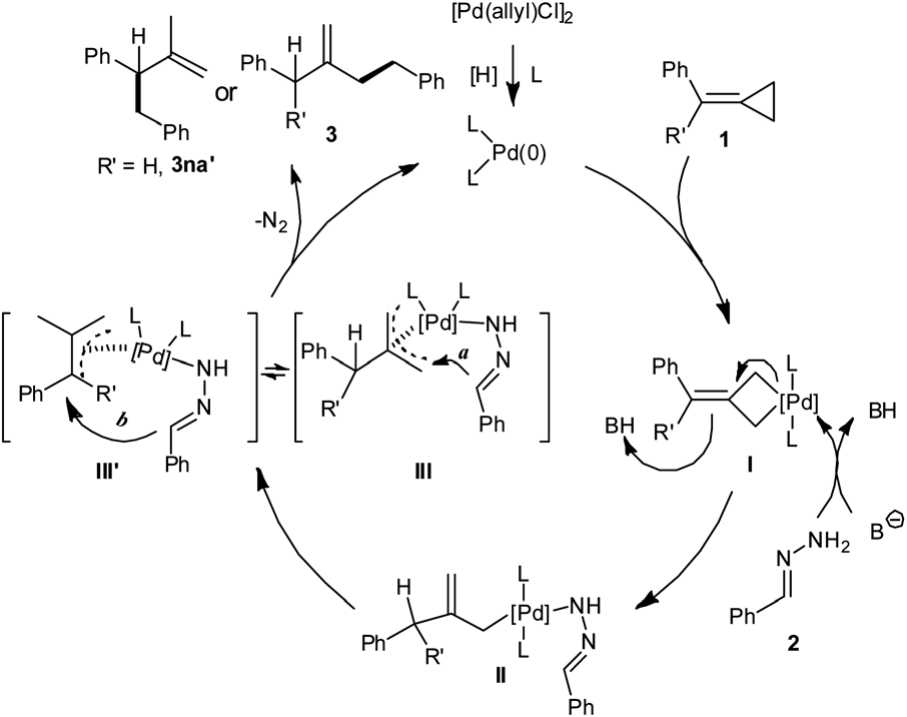
Proposed reaction mechanism.

Based on the results and previous studies, a plausible mechanism is proposed as illustrated in Scheme 3. Firstly, palladium(0) is generated from precatalyst [Pd(allyl)Cl]_2_ upon reduction possibly by the extra hydrazine. Then the direct insertion of palladium(0) species into the distal σ-bond of MCPs **1** gives palladacyclobutane **I**.^10*d*^ The base promotes the interaction of hydrazone **2** with palladacyclobutane **I** to form intermediate **II**. Then intermediate **II** leads to π-allyl–Pd species **III** or **III′**.^6,10*a*^ The decomposition of **III** with N_2_ extrusion releases product **3**, and completes the catalytic cycle (Scheme 3, route a).^17^ The formation of isomer **3na′** (when R′ = H) could be explained in terms of a possible isomerization of the π-allyl–Pd species of type **III** to **III′** probably due to the lower steric hindrance (Scheme 3, route b).^12^ The result of the deuterium-labelling experiment supports the proposed mechanism.

## Conclusions

In summary, we have developed a novel palladium-catalyzed ring-opening reaction of methylenecyclopropanes with simple hydrazones to produce the corresponding hydroalkylation products in good yields with high regioselectivities. Hydrazones originating from aryl and alkyl aldehydes successfully delivered the C-alkylated products, serving as surrogates of highly reactive organometallic reagents.

## Conflicts of interest

There are no conflicts to declare.

## Supplementary Material

SC-011-D0SC01221A-s001
