# Supplementary material for: Palladium-catalyzed hydroalkylation of methylenecyclopropanes with simple hydrazones
Source: Chem Sci. 2020 May 15;11(39):10759–63. doi: 10.1039/d0sc01221a (PMC8162302; doi:10.1039/d0sc01221a)
Supplement: SC-011-D0SC01221A-s001 [file SC-011-D0SC01221A-s001.pdf]

Electronic Supplementary Material (ESI) for Chemical Science.
This journal is © The Royal Society of Chemistry 2020

Supplementary Information

Palladium-catalyzed hydroalkylation of methylenecyclopropanes with simple hydrazones

Jinzhong Yao, [a, b] Zhangpei Chen, [a] Lin Yu, [a] Leiyang Lv, [a] Dawei Cao [a] and Chao-Jun Li *[a]

[a] *Department of Chemistry, FQRNT Center for Green Chemistry and Catalysis, McGill University, 801 Sherbooke Street West, Montreal, Quebec H3A 0B8, Canada*

[b] *College of Biological, Chemical Sciences and Engineering, Jiaxing University, Jiaxing 314001, People's Republic of China..*

Email: cj.li@mcgill.ca

TABLE OF CONTENTS

1. General information S2
2. Preparation of substrates S4
3. General procedure and characterization data for products S4
4. Deuterium-labelling study S16
5. Copies of NMR spectra for products S18

1. General information

Chemicals: All catalysts, ligands, bases and additives that are commercially available (Aldrich), were used without further purification: $[Pd(allyl)Cl_2]_2$ (Aldrich), $Pd_2(dba)_3$ (Aldrich), ligand (Aldrich), potassium tert-butoxide (Aldrich), sodium tert-butoxide (Aldrich), Lithium tert-butoxide (Aldrich), potassium hydroxide (Aldrich), sodium hydroxide (Aldrich), potassium phosphate (Aldrich), hydrazine hydrate (Reagent Grade, N_2H_4 64–65% wt, Aldrich), mesitylene (Aldrich), anhydrous sodium sulfate, and 4 Å Molecular Sieves powder (Aldrich). Hydrazine hydrate-*d6* was purchased from Toronto Research Chemicals. All other reagents were purchased from Alfa, Acros, Aldrich, and TCI and used without further purification.

Solvents: Tetrahydrofuran (THF) and 1,4-dioxane were taken directly from the *Pure Solvent MD-7* purification system (Innovative Technology). Solvents for filtration, transfers, chromatography and recrystallization were hexane (Fisher, ACS grade), pentane (ACS grade) and ethyl acetate (EtOAc) (Fisher, ACS grade).

NMR Spectroscopy: Nuclear magnetic resonance (^1H and ^{13}C NMR) spectra were recorded on a Bruker AV500 equipped with a 60-position SampleXpress sample changer (^1H, 500 MHz; ^{13}C, 125 MHz; ^{19}F, 470 MHz). Chemical shifts for both ^1H NMR and ^{13}C NMR spectra are expressed in parts per million (ppm) units downfield from TMS, with the solvent residue peak as the reference ($CDCl_3$: δ 7.26 ppm in ^1H NMR; δ 77.0 ppm in ^{13}C NMR). Data are reported as following: chemical shift, multiplicity (s = singlet, d = doublet, dd = doublet of doublets, t = triplet, td = triplet of doublets, dt = doublet of triplets, q = quartet, quin = quintet, sep = septet, m = multiplet, br = broad singlet), coupling constants *J* (Hz), and integration.

Mass Spectrometry: Mass spectrometry (MS) was performed by the McGill Chemistry Department Mass Spectrometry Facility. High Resolution Mass spectra were recorded using electrospray ionization (ESI+) and/or atmospheric pressure chemical ionization APCI (+/-), performed either on an "Exactive Plus Orbitrap" Thermo Scientific high resolution accurate mass (HR/AM) FT mass spectrometer, or a Bruker Daltonics Maxis Impact quadrupole-time of flight (QTOF) mass spectrometer.

Reaction Setup: All reactions were carried out in flamed-dried V-shaped microwave reaction vials, covered by aluminum seals with PTFE-faced silicone septa, under an atmosphere of nitrogen unless otherwise stated. All reported reaction temperatures correspond to oil bath temperatures. All air and moisture sensitive catalysts, ligands, and reagents were stored and charged in MBRAUN UNIlab Pro Glove Box Workstation.

Purifications: All work-up and purification procedures were carried out with reagent grade solvents. Analytical thin-layer chromatography (TLC) was performed using E. Merck silica gel 60 F254 pre-coated plates (0.25 mm). Flash column chromatography was performed with E. Merck silica gel P60 (40-63 μm particle size, 230–00 mesh) (SiO_2). Unless otherwise specified, "SiO_2" refers to P60 grade silica gel. Automated flash column chromatography was performed on Biotage Isolera™Spektra Systems with ACI™.

2. Preparation of substrates

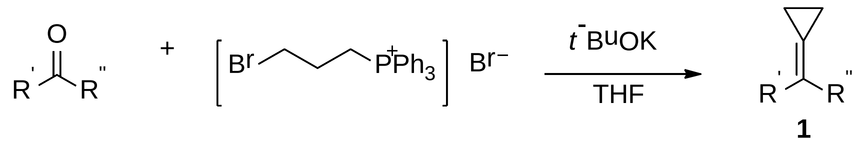

General procedure[1]: 3-bromopropyltriphenylphosphonium bromide (1.5 equiv) and *t*-BuOK (3.0 equiv) were suspended in dry THF at 0 °C in a three-neck round bottom flask under N_2. After the solution was stirred at room temperature for 30 min, the orange solution was then refluxed for 2 h before ketone or aldehyde (1.0 equiv) was added and stirring was continued at 80 °C for 12 h. The reaction mixture was quenched by water at room temperature, and the aqueous layer was extracted with hexane. The combined organic layers were washed with brine, dried over anhydrous $MgSO_4$, filtered and concentrated under reduced pressure. The crude reaction mixture was purified by flash column chromatography to obtain the MCP **1**.

3. General procedure and characterization data for products

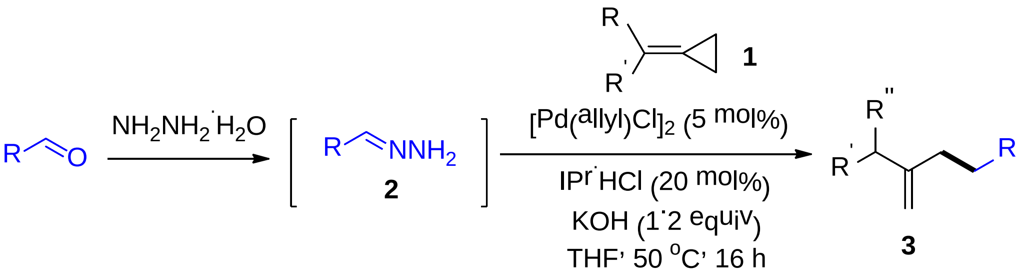

In situ preparation of hydrazone solution **2**: A mixture of aldehyde (0.2 mmol, 2.0 equiv) and hydrazine monohydrate (12 µL, 0.24 mmol, 64-65 wt%, 2.4 equiv) in THF (0.2 mL) solution was stirred at room temperature for 30 min. Before use, a small amount of anhydrous Na_2SO_4 and 4Å MS were added sequentially.

In a glovebox, a flame-dried reaction tube (10 cm^3) equipped with a magnetic stir bar was charged with $[Pd(allyl)Cl]_2$ (1.8 mg, 5 mol%), IPr•HCl (8.5 mg, 20 mol%), KOH (1.1 mg, 20 mol%) and THF (0.3 mL) before being sealed with a rubber septum. The reaction mixture was stirred at room temperature for 30 min. Then MCP **1** (0.1 mmol, 1.0 equiv), hydrazone solution **2** (0.2 mmol in 0.2 mL THF) and KOH (5.6 mg, 1.0 mmol) were added sequentially. After that, the reaction mixture was sealed with aluminum cap,

moved out of glovebox, and stirred at 50 °C for 16 hour. After the mixture was cooled to room temperature, the resulting solution was directly filtered through a pad of celite by EtOAc (3.0 mL). The solvent was evaporated in vacuo to give the crude product. NMR yield was determined by ^{1}H NMR using mesitylene as an internal standard. The residue was purified by preparative TLC (ethyl acetate/petroleum ether) to give the pure product **3**.

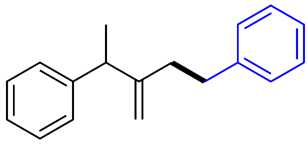

(3-methylenepentane-1,4-diyl)dibenzene (3aa, CAS: 1260690-81-0**):**[2] Colorless liquid (17.7 mg, 75%). Isolated by preparative TLC (hexane, R_f = 0.6); ^{1}H NMR (500 MHz, $CDCl_3$) δ 7.34 – 7.31 (m, 2H), 7.29 – 7.28 (m, 1H), 7.26 – 7.22 (m, 4H), 7.19 (t, *J* = 7.4 Hz, 1H), 7.14 – 7.13 (m, 2H), 5.05 (s, 1H), 5.01 (s, 1H), 3.48 (q, *J* = 7.0 Hz, 1H), 2.79 – 2.67 (m, 2H), 2.28 – 2.20 (m, 2H), 1.43 (d, *J* = 7.1 Hz, 3H); ^{13}C NMR (125 MHz, $CDCl_3$) δ 152.6, 145.3, 142.2, 128.4, 128.3, 128.3, 127.6, 126.2, 125.7, 109.1, 45.6, 36.8, 34.6, 20.7; HRMS (APCI) calcd for $C_{18}H_{19}$ [M – H$^-$] 235.1481, found 235.1488.

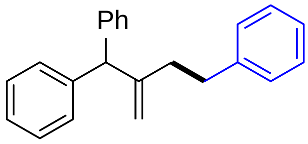

(2-methylenebutane-1,1,4-triyl)tribenzene (3ba): Colorless liquid (19.4 mg, 65%). Isolated by preparative TLC (hexane, R_f = 0.6); ^{1}H NMR (500 MHz, $CDCl_3$) δ 7.31 – 7.27 (m, 5H), 7.26 – 7.22 (m, 4H), 7.16 – 7.15 (m, 6H), 5.17 (s, 1H), 4.76 (s, 1H), 4.57 (s, 1H), 2.82 (t, *J* = 10.0 Hz, 2H), 2.38 (t, *J* = 10.0 Hz, 2H); ^{13}C NMR (125 MHz, $CDCl_3$) δ 151.4, 142.3, 141.9, 129.3, 128.4, 128.3, 128.2, 126.3, 125.8, 114.1, 57.7, 37.8, 34.7; HRMS (APCI) calcd for $C_{23}H_{23}$ [M + H$^+$] 299.1794, found 299.1800.

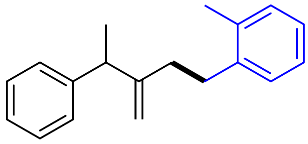

1-methyl-2-(3-methylene-4-phenylpentyl)benzene (3ab): Colorless liquid (19.5 mg, 78%). Isolated by preparative TLC (hexane, R_f = 0.6); ^{1}H NMR (500 MHz, $CDCl_3$) δ 7.32

(t, J = 7.5 Hz, 2H), 7.26 – 7.21 (m,, 3H), 7.13 – 7.09 (m, 3H), 7.07 – 7.05 (m, 1H), 5.07 (s, 1H), 5.04 (s, 1H), 3.50 (q, J = 7.0 Hz, 1H), 2.75 – 2.71(m, 1H), 2.69 – 2.64 (m, 1H), 2.22 (s, 3H), 2.20 – 2.12 (m, 2H), 1.43 (d, J = 7.1 Hz, 3H); ^{13}C NMR (125 MHz, $CDCl_3$) δ 152.9, 145.3, 140.4, 135.8, 130.1, 128.7, 128.4, 127.6, 126.2, 125.9, 109.1, 45.6, 35.5, 32.3, 20.7, 19.1; HRMS (APCI) calcd for $C_{19}H_{21}$ [M – H^-] 249.1638, found 249.1632.

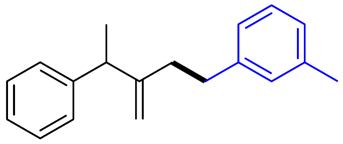

1-methyl-3-(3-methylene-4-phenylpentyl)benzene (3ac): Colorless liquid (21.0 mg, 84%). Isolated by preparative TLC (hexane, R_f = 0.6); ^1H NMR (500 MHz, $CDCl_3$) δ 7.34 – 7.31 (m, 2H), 7.25 – 7.22 (m, 3H), 7.17 (t, J = 7.5 Hz, 1H), 7.01 (d, J = 7.5 Hz, 1H), 6.94 (d, J = 8.5 Hz, 2H), 5.04 (s, 1H), 5.00 (s, 1H), 3.48 (q, J = 7.0 Hz, 1H), 2.76 – 2.63 (m, 2H), 2.34 (s, 3H), 2.30 – 2.15 (m, 2H), 1.43 (d, J = 7.1 Hz, 3H); ^{13}C NMR (125 MHz, $CDCl_3$) δ 152.7, 145.3, 142.2, 137.8, 129.1, 128.3, 128.2, 127.6, 126.5, 126.1, 125.3, 109.0, 45.6, 36.8, 34.5, 21.4, 20.7; HRMS (APCI) calcd for $C_{19}H_{21}$ [M – H^-] 249.1638, found 249.1630

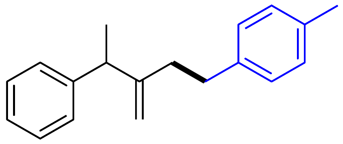

1-methyl-4-(3-methylene-4-phenylpentyl)benzene (3ad): Colorless liquid (19.8 mg, 79%). Isolated by preparative TLC (hexane, R_f = 0.6); ^1H NMR (500 MHz, $CDCl_3$) δ 7.32 (t, J = 7.5 Hz, 2H), 7.25 – 7.21 (m, 3H), 7.08 (d, J = 7.9 Hz, 2H), 7.02 (d, J = 7.9 Hz, 2H), 5.03 (s, 1H), 4.99 (s, 1H), 3.47 (q, J = 7.0 Hz, 1H), 2.74 – 2.62 (m, 2H), 2.33 (s, 3H), 2.28 – 2.14 (m, 2H), 1.42 (d, J = 7.1 Hz, 3H); ^{13}C NMR (125 MHz, $CDCl_3$) δ 152.7, 145.3, 139.1, 135.1, 128.9, 128.3, 128.2, 127.6, 126.1, 109.0, 45.6, 36.9, 34.1, 21.0, 20.6; HRMS (APCI) calcd for $C_{19}H_{21}$ [M – H^-] 249.1638, found 249.1634.

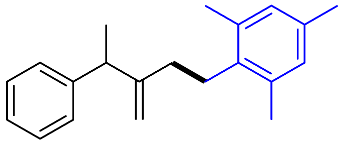

1,3,5-trimethyl-2-(3-methylene-4-phenylpentyl)benzene (3ae): Colorless liquid (11.1 mg, 40%). Isolated by preparative TLC (hexane, R_f = 0.5); ^1H NMR (500 MHz, $CDCl_3$) δ

7.33 – 7.30 (m, 2H), 7.27 – 7.25 (m, 2H), 7.23 – 7.20 (m, 1H), 6.81 (s, 2H), 5.10 (s, 1H), 5.09 (s, 1H), 3.53 (q, J = 7.0 Hz, 1H), 2.76 – 2.70 (m, 1H), 2.65 – 2.59 (m, 1H), 2.25 (s, 3H), 2.17 (s, 6H), 2.05 – 1.99 (m, 2H), 1.45 (d, J = 7.0 Hz, 3H); ^{13}C NMR (125 MHz, $CDCl_3$) δ 153.4, 145.3, 135.9, 134.9, 128.8, 128.3, 127.6, 126.2, 108.9, 45.6, 34.2, 28.7, 20.8, 20.7, 19.5; HRMS (APCI) calcd for $C_{21}H_{25}$ [M – H$^-$] 277.1951, found 277.1934.

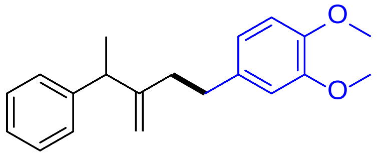

1,2-dimethoxy-4-(3-methylene-4-phenylpentyl)benzene (3af): Colorless liquid (19.0 mg, 64%). Isolated by preparative TLC (hexane: ethyl acetate = 20:1, R_f = 0.4); ^{1}H NMR (500 MHz, $CDCl_3$) δ 7.32 – 7.29 (m, 2H), 7.24 – 7.20 (m, 3H), 6.77 (d, J = 8.1 Hz, 1H), 6.65 (dd, J = 8.1, 2.0 Hz, 1H), 6.62 (d, J = 1.9 Hz, 1H), 5.03 (s, 1H), 4.98 (s, 1H), 3.86 (s, 3H), 3.85 (s, 3H), 3.45 (q, J = 7.0 Hz, 1H), 2.69 – 2.63 (m, 2H), 2.24 – 2.16 (m, 2H), 1.41 (d, J = 7.1 Hz, 3H); ^{13}C NMR (125 MHz, $CDCl_3$) δ 152.6, 148.7, 147.1, 145.3, 134.9, 128.3, 127.6, 126.1, 120.1, 111.7, 111.1, 109.1, 55.9, 55.8, 45.6, 36.9, 34.2, 20.7; HRMS (APCI) calcd for $C_{20}H_{25}O_2$ [M + H$^+$] 297.1849, found 297.1843.

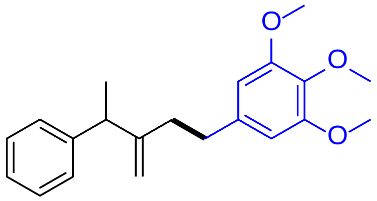

1,2,3-trimethoxy-5-(3-methylene-4-phenylpentyl)benzene (3ag): Colorless liquid (19.3 mg, 59%). Isolated by preparative TLC (hexane: ethyl acetate = 10:1, R_f = 0.3); ^{1}H NMR (500 MHz, $CDCl_3$) δ 7.31 (t, J = 7.4 Hz, 2H), 7.24 – 7.21 (m, 3H), 6.31 (s, 2H), 5.05 (s, 1H), 4.99 (s, 1H), 3.83 (s, 9H), 3.46 (q, J = 7.0 Hz, 1H), 2.69 – 2.62 (m, 2H), 2.26 – 2.18 (m, 2H), 1.42 (d, J = 7.0 Hz, 3H); ^{13}C NMR (125 MHz, $CDCl_3$) δ 153.0, 152.5, 145.2, 138.0, 136.0, 128.3, 127.6, 126.2, 109.2, 105.2, 60.9, 56.0, 45.6, 36.8, 35.0, 20.7; HRMS (APCI) calcd for $C_{21}H_{27}O_3$ [M + H$^+$] 327.1955, found 327.1958.

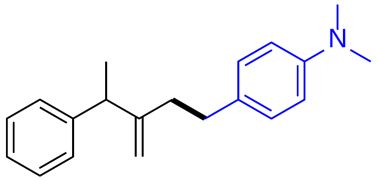

***N,N*-dimethyl-4-(3-methylene-4-phenylpentyl)aniline (3ah):** Yellow liquid (12.6 mg, 45%). Isolated by preparative TLC (hexane: ethyl acetate = 30:1, R_f = 0.4); ^{1}H NMR (500 MHz, $CDCl_3$) δ 7.29 (dd, *J* = 12.7, 5.3 Hz, 2H), 7.21 (dd, *J* = 14.6, 7.3 Hz, 3H), 7.00 (d, *J* = 8.4 Hz, 2H), 6.68 (d, *J* = 7.8 Hz, 2H), 5.00 (s, 1H), 4.97 (s, 1H), 3.46 (q, *J* = 7.0 Hz, 1H), 2.91 (s, 6H), 2.64 – 2.58 (m, 2H), 2.21 – 2.13 (m, 2H), 1.40 (d, *J* = 7.1 Hz, 3H); ^{13}C NMR (125 MHz, $CDCl_3$) δ 152.9, 145.4, 128.9, 128.3, 127.6, 126.1, 113.1, 108.9, 45.6, 41.0, 37.1, 33.6, 20.6; HRMS (APCI) calcd for $C_{20}H_{26}N$ [M + H$^+$] 280.2060, found 280.2061.

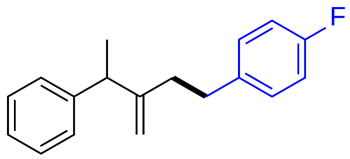

1-fluoro-4-(3-methylene-4-phenylpentyl)benzene (3ai): Colorless liquid (22.6 mg, 89%). Isolated by preparative TLC (hexane, R_f = 0.6); ^{1}H NMR (500 MHz, $CDCl_3$) δ 7.33 – 7.30 (m, 2H), 7.24 – 7.21 (m, 3H), 7.05 (dd, *J* = 8.7, 5.5 Hz, 2H), 6.94 (t, *J* = 8.8 Hz, 2H), 5.04 (s, 1H), 4.97 (s, 1H), 3.45 (q, *J* = 7.0 Hz, 1H), 2.75 – 2.63 (m, 2H), 2.26 – 2.16 (m, 2H), 1.41 (d, *J* = 7.1 Hz, 3H); ^{13}C NMR (125 MHz, $CDCl_3$) δ 161.2 (*J* = 241.3 Hz), 152.2, 145.1, 137.7 (*J* = 3.8 Hz), 129.6 (*J* = 7.5 Hz), 128.4, 127.5, 126.2, 114.9 (*J* = 21.3 Hz), 109.3, 45.6, 36.8, 33.7, 20.6; ^{19}F NMR (470 MHz, $CDCl_3$) δ -117.9; HRMS (APCI) calcd for $C_{18}H_{18}F$ [M – H$^-$] 253.1387, found 253.1391.

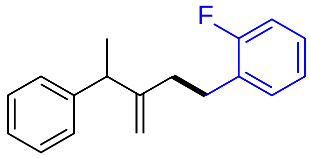

1-fluoro-4-(3-methylene-4-phenylpentyl)benzene (3aj): Colorless liquid (21.9 mg, 86%). Isolated by preparative TLC (hexane, R_f = 0.6); ^{1}H NMR (500 MHz, $CDCl_3$) δ 7.33 – 7.30 (m, 2H), 7.24 – 7.22 (m, 3H), 7.17 – 7.15 (m, 1H), 7.09 – 7.07 (m, 1H), 7.04 – 6.99 (m, 2H), 5.03 (s, 1H), 4.98 (s, 1H), 3.48 (q, *J* = 7.0 Hz, 1H), 2.78 – 2.70 (m, 2H), 2.28 – 2.15 (m, 2H), 1.41 (d, *J* = 7.1 Hz, 3H); ^{13}C NMR (125 MHz, $CDCl_3$) δ 161.1 (*J* = 243.8 Hz), 152.2, 145.2, 130.5 (*J* = 5.0 Hz), 128.9 (*J* = 16.3 Hz), 128.3, 127.5, 127.4 (*J* = 8.8 Hz), 126.1, 123.8 (*J* = 3.8 Hz), 115.1 (*J* = 21.3 Hz), 109.4, 45.4, 35.3, 27.8 (*J* = 1.3 Hz), 20.6; ^{19}F NMR (470 MHz, $CDCl_3$) δ -118.9; HRMS (APCI) $C_{18}H_{18}F$ [M – H$^-$]

253.1387, found 253.1378.

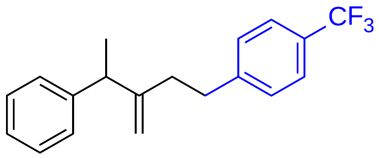

1-(3-methylene-4-phenylpentyl)-4-(trifluoromethyl)benzene (3ak): Colorless liquid (26.2 mg, 86%). Isolated by preparative TLC (hexane, $R_f = 0.4$); ^{1}H NMR (500 MHz, $CDCl_3$) δ 7.50 (d, J = 8.1 Hz, 2H), 7.32 (t, J = 7.4 Hz, 2H), 7.24 – 7.19 (m, 5H), 5.06 (s, 1H), 4.97 (s, 1H), 3.45 (q, J = 7.0 Hz, 1H), 2.81 – 2.70 (m, 2H), 2.29 – 2.16 (m, 2H), 1.41 (d, J = 7.1 Hz, 3H); ^{13}C NMR (125 MHz, $CDCl_3$) δ 151.8, 146.2, 145.0, 128.6, 128.4, 128.1 (J = 32.5 Hz), 127.5, 126.3, 125.1 (J = 3.8 Hz), 124.4 (J = 270 Hz), 109.5, 45.6, 36.2, 34.3, 20.6; ^{19}F NMR (470 MHz, $CDCl_3$) δ -64.9; HRMS (APCI) calcd for $C_{19}H_{18}F_3$ [M – H^-] 303.1355, found 303.1343.

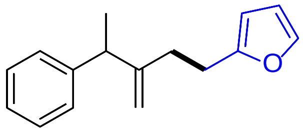

2-(3-methylene-4-phenylpentyl)furan (3al): Colorless liquid (20.4 mg, 90%). Isolated by preparative TLC (hexane, $R_f = 0.5$); ^{1}H NMR (500 MHz, $CDCl_3$) δ 7.32 – 7.27 (m, 3H), 7.23 – 7.19 (m, 3H), 6.26 (dd, J = 3.0, 1.9 Hz, 1H), 5.92 (dd, J = 3.1, 0.6 Hz, 1H), 5.02 (s, 1H), 4.95 (s, 1H), 3.44 (q, J = 7.0 Hz, 1H), 2.78 – 2.67 (m, 2H), 2.33 – 2.18 (m, 2H), 1.40 (d, J = 7.1 Hz, 3H); ^{13}C NMR (125 MHz, $CDCl_3$) δ 155.8, 151.9, 145.1, 140.7, 128.4, 127.5, 126.2, 110.0, 109.3, 104.7, 45.5, 33.1, 26.7, 20.7; HRMS (APCI) calcd for $C_{16}H_{19}O$ [M + H^+] 227.1430, found 227.1433.

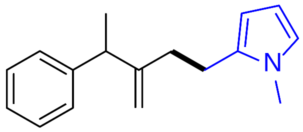

1-methyl-2-(3-methylene-4-phenylpentyl)-1H-pyrrole (3am): Yellow liquid (18.2 mg, 76%). Isolated by preparative TLC (hexane: ethyl acetate = 50:1, $R_f = 0.3$); ^{1}H NMR (500 MHz, $CDCl_3$) δ 7.32 – 7.27 (m, 2H), 7.24 – 7.19 (m, 3H), 6.51 (s, 1H), 6.03 (d, J = 3.2 Hz, 1H), 5.82 (d, J = 3.3 Hz, 1H), 5.06 (s, 1H), 5.01 (s, 1H), 3.47 (q, J = 7.1 Hz, 1H), 3.42 (s, 3H), 2.66 – 2.54 (m, 2H), 2.26 – 2.19 (m, 2H), 1.42 (d, J = 7.1 Hz, 3H); ^{13}C NMR (125 MHz, $CDCl_3$) δ 152.5, 145.2, 133.1, 128.4, 127.6, 126.2, 121.0, 109.2, 106.5, 105.4,

45.6, 34.2, 33.4, 25.3, 20.7; HRMS (APCI) calcd for $C_{17}H_{22}N$ [M + H$^+$] 240.1747, found 240.1748.

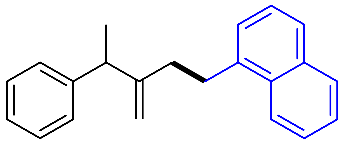

1-(3-methylene-4-phenylpentyl)naphthalene (3an): Colorless liquid (24.3 mg, 85%). Isolated by preparative TLC (hexane, R_f = 0.5); ^{1}H NMR (500 MHz, $CDCl_3$) δ 7.81 (d, J = 7.8 Hz, 1H), 7.76 (t, J = 7.1 Hz, 2H), 7.55 (s, 1H), 7.44 (td, J = 14.7, 6.8 Hz, 2H), 7.32 (t, J = 7.2 Hz, 2H), 7.27 – 7.22 (m, 4H), 5.06 (s, 1H), 5.03 (s, 1H), 3.50 (q, J = 7.0 Hz, 1H), 2.95 – 2.83 (m, 2H), 2.40 – 2.25 (m, 2H), 1.43 (d, J = 8.2 Hz, 3H); ^{13}C NMR (125 MHz, $CDCl_3$) δ 152.5, 145.2, 139.7, 133.6, 132.0, 128.4, 127.7, 127.6, 127.4, 127.3, 126.3, 126.2, 125.8, 125.1, 109.3, 45.7, 36.6, 34.7, 20.7; HRMS (APCI) calcd for $C_{22}H_{23}$ [M + H$^+$] 287.1794, found 287.789.

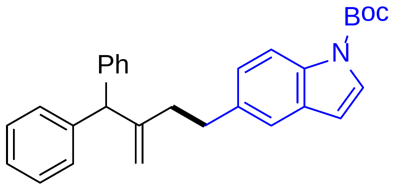

***tert*-butyl 5-(3-benzhydrylbut-3-en-1-yl)-1*H*-indole-1-carboxylate (3bo):** Yellow liquid (28.9 mg, 66%). Isolated by preparative TLC (hexane: ethyl acetate = 10:1, R_f = 0.3); ^{1}H NMR (500 MHz, $CDCl_3$) δ 8.02 (d, J = 7.0 Hz, 1H), 7.57 (s, 1H), 7.32 – 7.27 (m, 5H), 7.22 (t, J = 7.3 Hz, 2H), 7.16 (d, J = 7.3 Hz, 4H), 7.11 (d, J = 8.3 Hz, 1H), 6.50 (d, J = 3.5 Hz, 1H), 5.18 (s, 1H), 4.77 (s, 1H), 4.57 (s, 1H), 2.92 – 2.89 (m, 2H), 2.44 – 2.41 (m, 2H), 1.68 (s, 9H); ^{13}C NMR (125 MHz, $CDCl_3$) δ 151.5, 142.3, 136.3, 130.7, 129.3, 128.3, 128.2, 126.3, 126.0, 124.9, 120.3, 114.9, 114.1, 107.1, 83.5, 57.7, 38.3, 34.7, 28.2; HRMS (APCI) calcd for $C_{30}H_{31}NO_2Na$ [M +Na$^+$] 460.2247, found 460.2258.

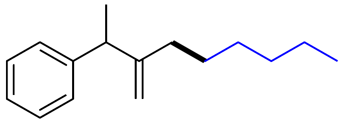

(3-methylenenonan-2-yl)benzene (3ap): Colorless liquid (9.5 mg, 44%). Isolated by preparative TLC (hexane, R_f = 0.6); ^{1}H NMR (500 MHz, $CDCl_3$) δ 7.32 – 7.28 (m, 2H), 7.23 – 7.19 (m, 3H), 4.95 (s, 1H), 4.91 (s, 1H), 3.43 (q, J = 7.0 Hz, 1H), 1.97 – 1.82 (m,

2H), 1.43 – 1.38 (m, 1H), 1.39 (d, J = 7.1 Hz, 3H), 1.30 – 1.21 (m, 7H), 0.88 (t, J = 7.0 Hz, 3H); ^{13}C NMR (125 MHz, $CDCl_3$) δ 153.4, 145.6, 128.2, 127.5, 126.0, 108.5, 45.2, 35.1, 31.8, 29.1, 27.9, 22.6, 20.8, 14.1; HRMS (APCI) calcd for $C_{16}H_{23}$ [M – H$^-$] 215.1794, found 215.1790.

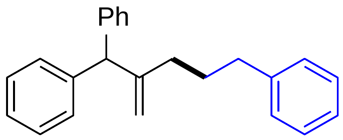

(2-methylenepentane-1,1,5-triyl)tribenzene (3bq): Colorless liquid (15.6 mg, 50%). Isolated by preparative TLC (hexane, R_f = 0.6); ^{1}H NMR (500 MHz, $CDCl_3$) δ 7.33 – 7.30 (m, 6H), 7.28 – 7.22 (m, 3H), 7.19 – 7.18 (m, 6H), 5.14 (s, 1H), 4.79 (s, 1H), 4.55 (s, 1H), 2.63 – 2.60 (m, 2H), 2.16 – 2.13 (m, 2H), 1.87 – 1.84 (m, 2H); ^{13}C NMR (125 MHz, $CDCl_3$) δ 151.6, 142.5, 142.4, 129.3, 128.4, 128.3, 128.2, 126.3, 125.7, 113.9, 57.4, 35.9, 35.6, 29.9; HRMS (APCI) calcd for $C_{24}H_{25}$ [M + H$^+$] 313.1951, found 313.1954.

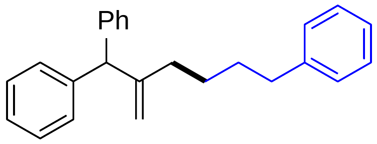

(2-methylenehexane-1,1,6-triyl)tribenzene (3br): Colorless liquid (13.1 mg, 40%). Isolated by preparative TLC (hexane, R_f = 0.6); ^{1}H NMR (500 MHz, $CDCl_3$) δ 7.31 – 7.27 (m, 6H), 7.23 – 7.19 (m, 3H), 7.19 – 7.14 (m, 6H), 5.09 (s, 1H), 4.75 (s, 1H), 4.49 (s, 1H), 2.60 (t, J = 7.5 Hz, 2H), 2.10 (t, J = 7.3 Hz, 2H), 1.62 (dd, J = 14.6, 7.5 Hz, 2H), 1.55 (dt, J = 13.7, 6.9 Hz, 2H); ^{13}C NMR (125 MHz, $CDCl_3$) δ 151.8, 142.6, 142.5, 129.3, 128.4, 128.3, 128.2, 126.3, 125.6, 113.7, 57.3, 36.0, 35.8, 31.0, 27.5; HRMS (APCI) calcd for $C_{25}H_{27}$ [M + H$^+$] 327.2107, found 327.2111.

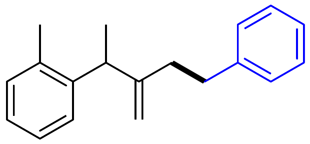

1-methyl-2-(3-methylene-5-phenylpentan-2-yl)benzene (3ca): Colorless liquid (16.0 mg, 64%). Isolated by preparative TLC (hexane, R_f = 0.6); ^{1}H NMR (500 MHz, $CDCl_3$) δ 7.26 (t, J = 7.4 Hz, 2H), 7.20 – 7.16 (m, 4H), 7.13 – 7.11 (m, 3H), 5.02 (s, 1H), 4.97 (s, 1H), 3.67 (q, J = 6.9 Hz, 1H), 2.73 – 2.69 (m, 2H), 2.35 (s, 3H), 2.30 – 2.24 (m, 1H), 2.20 – 2.13 (m, 1H), 1.36 (d, J = 7.0 Hz, 3H); ^{13}C NMR (125 MHz, $CDCl_3$) δ 152.4, 143.2,

142.2, 135.8, 130.3, 128.3, 128.2, 126.5, 126.1, 125.9, 125.7, 109.5, 41.0, 37.0, 34.7, 20.0, 19.5; HRMS (APCI) calcd for $C_{19}H_{21}$ [M – H$^-$] 249.1638, found 249.1642.

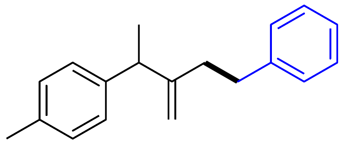

1-methyl-4-(3-methylene-5-phenylpentan-2-yl)benzene (3da): Colorless liquid (16.8 mg, 67%). Isolated by preparative TLC (hexane, R_f = 0.6); ^{1}H NMR (500 MHz, $CDCl_3$) δ 7.26 (t, J = 7.4 Hz, 2H), 7.17 (t, J = 7.4 Hz, 1H), 7.12 (d, J = 6.2 Hz, 6H), 5.01 (s, 1H), 4.96 (s, 1H), 3.42 (q, J = 7.0 Hz, 1H), 2.77 – 2.65 (m, 2H), 2.35 (s, 3H), 2.26 – 2.18 (m, 2H), 1.39 (d, J = 7.1 Hz, 3H); ^{13}C NMR (125 MHz, $CDCl_3$) δ 152.8, 142.3, 142.2, 135.6, 129.0, 128.3, 128.2, 127.4, 125.7, 108.9, 45.2, 36.7, 34.6, 21.0, 20.7; HRMS (APCI) calcd for $C_{19}H_{23}$ [M + H$^+$] 251.1794, found 251.1798.

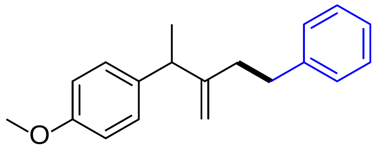

1-methoxy-4-(3-methylene-5-phenylpentan-2-yl)benzene (3ea): Colorless liquid (16.2 mg, 61%). Isolated by preparative TLC (hexane: ethyl acetate = 50:1, R_f = 0.3); ^{1}H NMR (500 MHz, $CDCl_3$) δ 7.26 (t, J = 7.5 Hz, 2H), 7.18 (t, J = 7.3 Hz, 1H), 7.13 (t, J = 8.7 Hz, 4H), 6.85 (d, J = 8.6 Hz, 2H), 5.00 (s, 1H), 4.96 (s, 1H), 3.82 (s, 3H), 3.41 (q, J = 7.0 Hz, 1H), 2.78 – 2.65 (m, 2H), 2.27 – 2.17 (m, 2H), 1.38 (d, J = 7.1 Hz, 3H); ^{13}C NMR (125 MHz, $CDCl_3$) δ 157.9, 152.9, 142.3, 137.3, 128.4, 128.3, 128.2, 125.7, 113.7, 108.8, 55.3, 44.8, 36.6, 34.6, 20.7; HRMS (APCI) calcd for $C_{19}H_{23}O$ [M + H$^+$] 267.1743, found 267.1748.

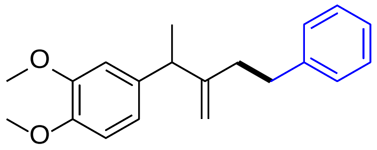

1-methoxy-4-(3-methylene-5-phenylpentan-2-yl)benzene (3fa): Colorless liquid (25.2 mg, 85%). Isolated by preparative TLC (hexane: ethyl acetate = 20:1, R_f = 0.4); ^{1}H NMR (500 MHz, $CDCl_3$) δ 7.27 (dd, J = 12.0, 4.6 Hz, 2H), 7.18 (t, J = 7.4 Hz, 1H), 7.12 (d, J = 7.1 Hz, 2H), 6.82 (d, J = 8.2 Hz, 1H), 6.78 (dd, J = 8.2, 1.9 Hz, 1H), 6.73 (d, J = 1.9 Hz, 1H), 5.02 (s, 1H), 4.97 (s, 1H), 3.89 (s, 3H), 3.88 (s, 3H), 3.41 (q, J = 7.0 Hz, 1H), 2.74 –

2.69 (m, 2H), 2.30 – 2.16 (m, 2H), 1.40 (d, J = 7.1 Hz, 3H); ^{13}C NMR (125 MHz, $CDCl_3$) δ 152.8, 148.8, 147.4, 142.2, 137.9, 128.3, 128.2, 125.7, 119.5, 111.0, 110.7, 108.9, 55.9, 55.8, 45.2, 36.6, 34.6, 20.7; HRMS (APCI) calcd for $C_{20}H_{25}O_2$ [M + H$^+$] 297.1849, found 297.1856.

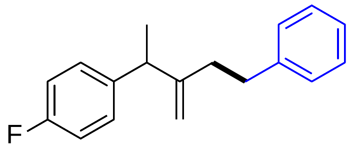

1-fluoro-4-(3-methylene-5-phenylpentan-2-yl)benzene (3ga): Colorless liquid (13.7 mg, 54%). Isolated by preparative TLC (hexane, R_f = 0.5); ^{1}H NMR (500 MHz, $CDCl_3$) δ 7.25 (t, J = 7.4 Hz, 2H), 7.18 – 7.14 (m, 3H), 7.10 (d, J = 7.0 Hz, 2H), 6.97 (t, J = 8.7 Hz, 2H), 5.00 (s, 1H), 4.97 (s, 1H), 3.42 (q, J = 7.0 Hz, 1H), 2.75 – 2.64 (m, 2H), 2.25 – 2.14 (m, 2H), 1.37 (d, J = 7.1 Hz, 3H); ^{13}C NMR (125 MHz, $CDCl_3$) δ 161.4 (J = 241.3 Hz), 152.4, 142.1, 140.8 (J = 2.5 Hz), 128.9 (J = 8.8 Hz), 128.3, 128.3, 125.8, 115.0 (J = 20.0 Hz), 109.3, 44.9, 36.6, 34.5, 20.7; ^{19}F NMR (470 MHz, $CDCl_3$) δ -117.4; HRMS (APCI) calcd for $C_{18}H_{18}F$ [M – H$^-$] 253.1387, found 253.1391.

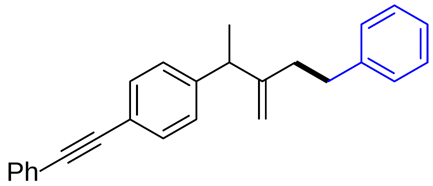

1-(3-methylene-5-phenylpentan-2-yl)-4-(phenylethynyl)benzene (3ha): Colorless liquid (20.9 mg, 62%). Isolated by preparative TLC (hexane, R_f = 0.5); ^{1}H NMR (500 MHz, $CDCl_3$) δ 7.56 – 7.54 (m, 2H), 7.48 (d, J = 8.3 Hz, 2H), 7.39 – 7.35 (m, 3H), 7.26 (d, J = 7.6 Hz, 2H), 7.19 (dd, J = 17.9, 7.7 Hz, 3H), 7.12 (d, J = 7.4 Hz, 2H), 5.04 (s, 1H), 5.01 (s, 1H), 3.47 (q, J = 7.0 Hz, 1H), 2.78 – 2.66 (m, 2H), 2.29 – 2.17 (m, 2H), 1.41 (d, J = 7.1 Hz, 3H); ^{13}C NMR (125 MHz, $CDCl_3$) δ 152.1, 145.7, 142.1, 131.7, 131.6, 128.3, 128.3, 128.3, 128.1, 127.6, 125.8, 123.4, 121.0, 109.5, 89.5, 88.9, 45.5, 36.7, 34.5, 20.4; HRMS (APCI) calcd for $C_{26}H_{25}$ [M + H$^+$] 337.1951, found 337.1950.

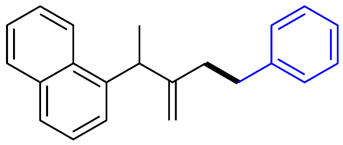

1-(3-methylene-5-phenylpentan-2-yl)naphthalene (3ia): Colorless liquid (24.3 mg, 85%). Isolated by preparative TLC (hexane, R_f = 0.5); ^{1}H NMR (500 MHz, $CDCl_3$) δ 7.82 (dd, J = 18.2, 8.8 Hz, 3H), 7.67 (s, 1H), 7.51 – 7.46 (m, 2H), 7.38 (dd, J = 8.4, 1.7 Hz, 1H), 7.24 (t, J = 7.4 Hz, 2H), 7.17 (t, J = 7.3 Hz, 1H), 7.11 (d, J = 7.2 Hz, 2H), 5.11 (s, 1H), 5.05 (s, 1H), 3.64 (q, J = 7.0 Hz, 1H), 2.82 – 2.69 (m, 2H), 2.30 – 2.22 (m, 2H), 1.50 (d, J = 7.0 Hz, 3H); ^{13}C NMR (125 MHz, $CDCl_3$) δ 152.4, 142.7, 142.1, 133.6, 132.3, 128.3, 128.2, 127.9, 127.6, 127.6, 126.3, 125.9, 125.7, 125.3, 109.4, 45.7, 36.7, 34.5, 20.6; HRMS (APCI) calcd for $C_{22}H_{23}$ $[M + H^+]$ 287.1794, found 287.1797.

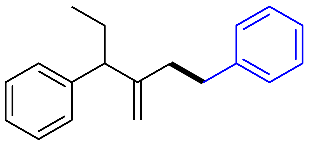

(3-methylenehexane-1,4-diyl)dibenzene (3ja): Colorless liquid (16.5 mg, 66%). Isolated by preparative TLC (hexane, R_f = 0.6); ^{1}H NMR (500 MHz, $CDCl_3$) δ 7.32 – 7.29 (m, 2H), 7.28 – 7.22 (m, 5H), 7.18 (t, J = 7.3 Hz, 1H), 7.11 (d, J = 7.4 Hz, 2H), 5.05 (s, 1H), 4.97 (s, 1H), 3.14 (t, J = 7.5 Hz, 1H), 2.73 – 2.66 (m, 2H), 2.23 – 2.16 (m, 2H), 1.94 – 1.88 (m, 1H),, 1.79 – 1.75 (m, 1H), 0.87 (t, J = 7.3 Hz, 3H); ^{13}C NMR (125 MHz, $CDCl_3$) δ 151.6, 143.8, 142.2, 128.3, 128.2, 128.2, 128.1, 126.2, 125.7, 109.2, 53.8, 36.6, 34.5, 26.7, 12.6; HRMS (APCI) calcd for $C_{19}H_{21}$ $[M – H^-]$ 249.1638, found 249.1641.

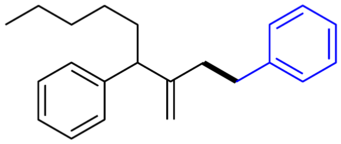

(3-methylenenonane-1,4-diyl)dibenzene (3ka): Colorless liquid (23.7 mg, 81%). Isolated by preparative TLC (hexane, R_f = 0.6); ^{1}H NMR (500 MHz, $CDCl_3$) δ 7.32 – 7.31 (m, 2H), 7.27 (t, J = 7.4 Hz, 2H), 7.25 – 7.17 (m, 4H), 7.12 (d, J = 7.1 Hz, 2H), 5.05 (s, 1H), 4.96 (s, 1H), 3.24 (t, J = 7.5 Hz, 1H), 2.75 – 2.63 (m, 2H), 2.24 – 2.16 (m, 2H), 1.87 – 1.83 (m, 1H), 1.75 – 1.72 (m, 1H), 1.30 – 1.18 (m, 6H), 0.89 (t, J = 6.7 Hz, 3H); ^{13}C NMR (125 MHz, $CDCl_3$) δ 151.8, 144.0, 142.2, 128.3, 128.2, 128.2, 128.0, 126.1, 125.7, 109.1, 51.9, 36.6, 34.5, 33.8, 32.0, 27.6, 22.6, 14.1; HRMS (APCI) calcd for $C_{22}H_{27}$ $[M – H^-]$ 291.2107, found 291.2109.

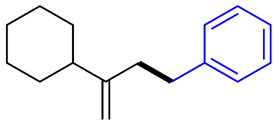

(3-cyclohexylbut-3-en-1-yl)benzene (3la): Colorless liquid (16.9 mg, 79%). Isolated by preparative TLC (hexane, R_f = 0.6); ^{1}H NMR (500 MHz, $CDCl_3$) δ 7.31 (t, J = 7.5 Hz, 2H), 7.22 (dd, J = 13.6, 7.1 Hz, 3H), 4.80 (s, 1H), 4.78 (d, J = 1.4 Hz, 1H), 2.79 – 2.76 (m, 2H), 2.37 – 2.35 (m, 2H), 1.91 (t, J = 12.7 Hz, 1H), 1.82 – 1.79 (m, 4H), 1.72 (d, J = 14.0 Hz, 1H), 1.34 – 1.27 (m, 2H), 1.23 – 1.15 (m, 3H); ^{13}C NMR (125 MHz, $CDCl_3$) δ 154.9, 142.5, 128.4, 128.3, 125.7, 107.1, 44.5, 36.7, 34.8, 32.5, 26.8, 26.4; HRMS (APCI) calcd for $C_{16}H_{23}$ [M + H$^+$] 215.1794, found 215.1800.

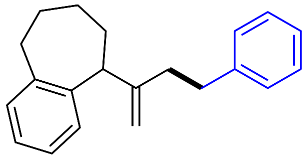

5-(4-phenylbut-1-en-2-yl)-6,7,8,9-tetrahydro-5*H*-benzo[7]annulene (3ma): Colorless liquid (21.6 mg, 78%). Isolated by preparative TLC (hexane, R_f = 0.6); ^{1}H NMR (500 MHz, $CDCl_3$) δ 7.31 – 7.28 (m, 2H), 7.22 – 7.19 (m, 3H), 7.14 – 7.09 (m, 4H), 5.15 (s, 1H), 4.79 (s, 1H), 3.56 (d, J = 8.2 Hz, 1H), 2.84 (dd, J = 14.0, 6.2 Hz, 4H), 2.46 – 2.33 (m, 2H), 1.98 – 1.72 (m, 6H); ^{13}C NMR (125 MHz, $CDCl_3$) δ 151.1, 143.6, 143.3, 142.2, 129.6, 128.4, 128.3, 127.9, 126.1, 126.0, 125.8, 111.7, 50.2, 37.8, 36.2, 34.7, 32.0, 29.6, 27.9; HRMS (APCI) calcd for $C_{21}H_{25}$ [M + H$^+$] 277.1951, found 277.1957.

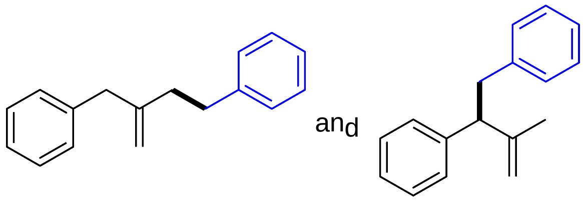

(2-methylenebutane-1,4-diyl)dibenzene (3na) and (3-methylbut-3-ene-1,2-diyl)dibenzene (3na'): Colorless liquid (mixture of **3na** and **3na'** with ratio 3: 2, 13.3 mg, 60%). Isolated by preparative TLC (hexane, R_f = 0.6); ^{1}H NMR (500 MHz, $CDCl_3$) for **3na** δ 7.32 – 7.24 (m, 4H), 7.24 – 7.14 (m, 6H), 4.89 (s, 1H), 4.82 (s, 1H), 3.39 (s, 2H), 2.79 – 2.75 (m, 2H), 2.32 – 2.28 (m, 2H); for **3na'** δ 7.32 – 7.24 (m, 2H), 7.24 – 7.14 (m, 6H), 7.04 (d, J = 7.0 Hz, 2H), 4.95 (s, 1H), 4.88 (s, 1H), 3.53 (t, J = 7.6 Hz, 1H), 3.20 (dd, J = 13.6, 7.0 Hz, 1H), 3.02 (dd, J = 13.6, 8.3 Hz, 1H),

1.64 (s, 3H). ^{13}C NMR (125 MHz, $CDCl_3$) δ 148.5, 147.4, 142.9, 142.1, 140.6, 139.6, 129.0, 129.0, 128.4, 128.3, 128.3, 128.2, 128.0, 128.0, 126.3, 126.1, 125.8, 125.8, 111.6, 110.9, 54.5, 43.4, 40.0, 37.1, 34.3, 21.6; HRMS (APCI) calcd for $C_{17}H_{19}$ [M + H$^+$] 223.1481, found 223.1486.

4. Deuterium-labelling study

The operations were following the general procedure. Hydrazone **2a-*d*^{2}** (78% D). ^{1}H NMR (500 MHz, $CDCl_3$) δ 7.76 (s, 1H), 7.55 (d, *J*= 7.4 Hz, 2H), 7.37 – 7.26 (m, 3H), 5.46 (br, 0.44H). Product **3ba-*d*^{2}**, 60% yield. ^{1}H NMR (500 MHz, $CDCl_3$) δ 7.31 – 7.26 (m, 6H), 7.23-7.19 (m, 3H), 7.16 (d, *J* = 7.2 Hz, 6H), 5.17 (s, 1H), 4.76 (s, 0.48H), 4.57 (s, 1H), 2.80 (t, *J* = 8.2 Hz, 1.22H), 2.39 (t, *J* = 7.7 Hz, 2H); ^{2}H NMR (77 MHz, $CDCl_3$) δ 4.34, 2.39. Deuterium incorporation was determined by ^{1}H NMR.

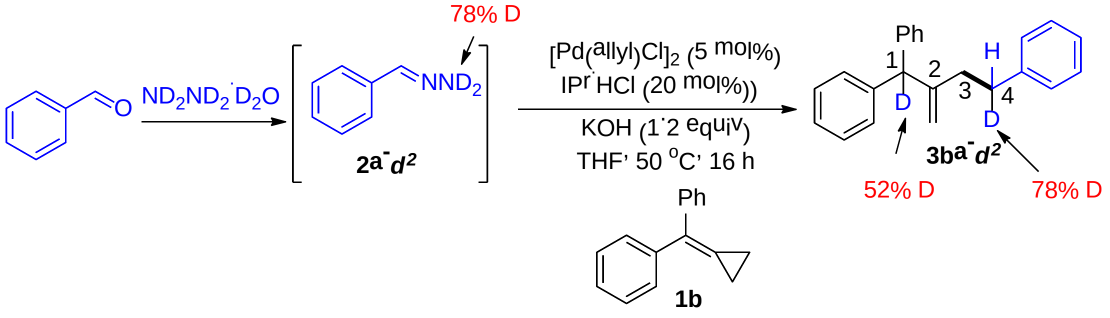

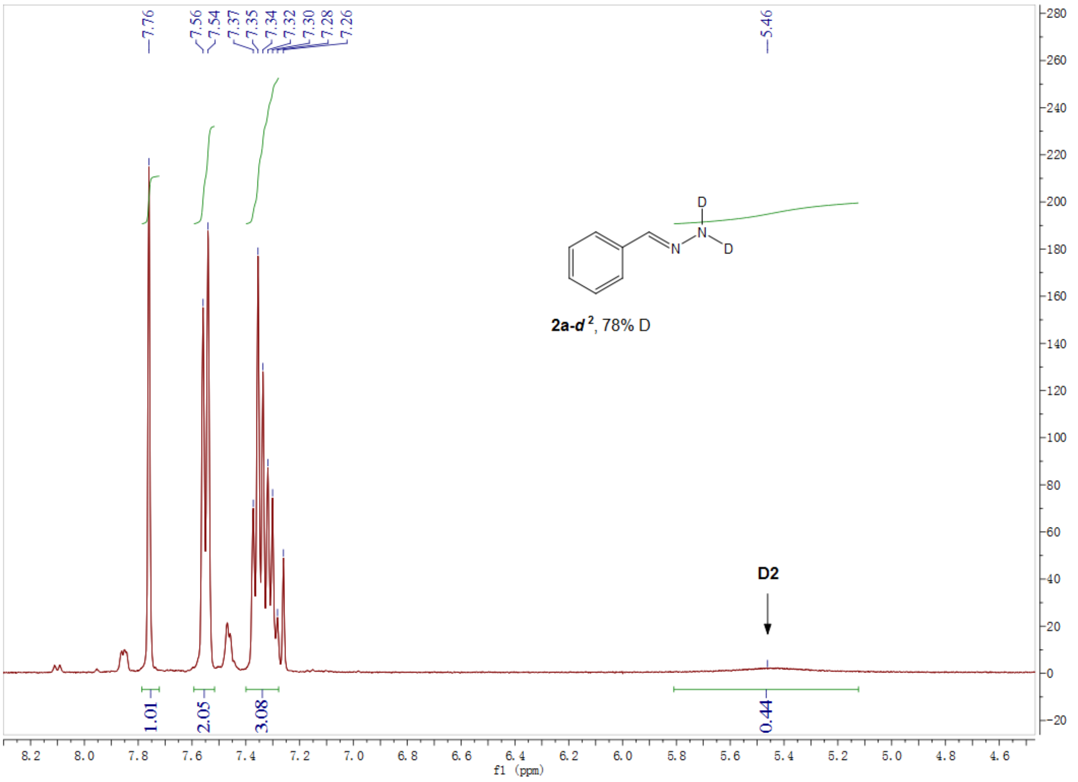
2a-d², 78% D
D2

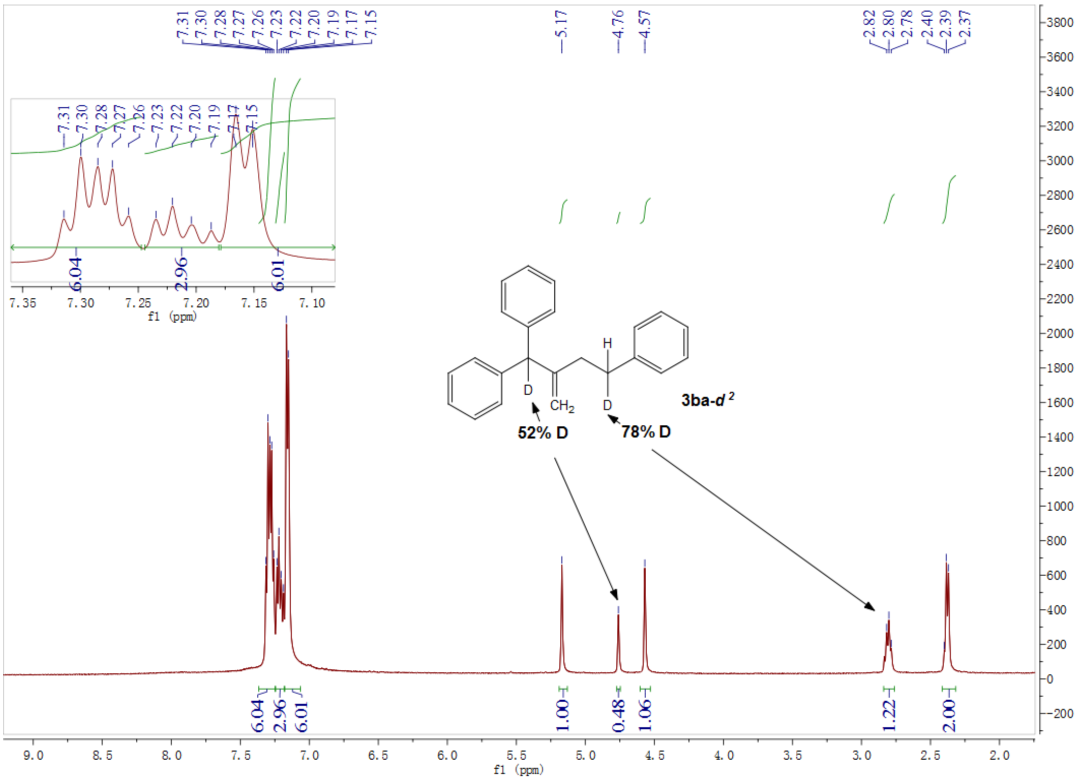
3ba-d²
52% D
78% D

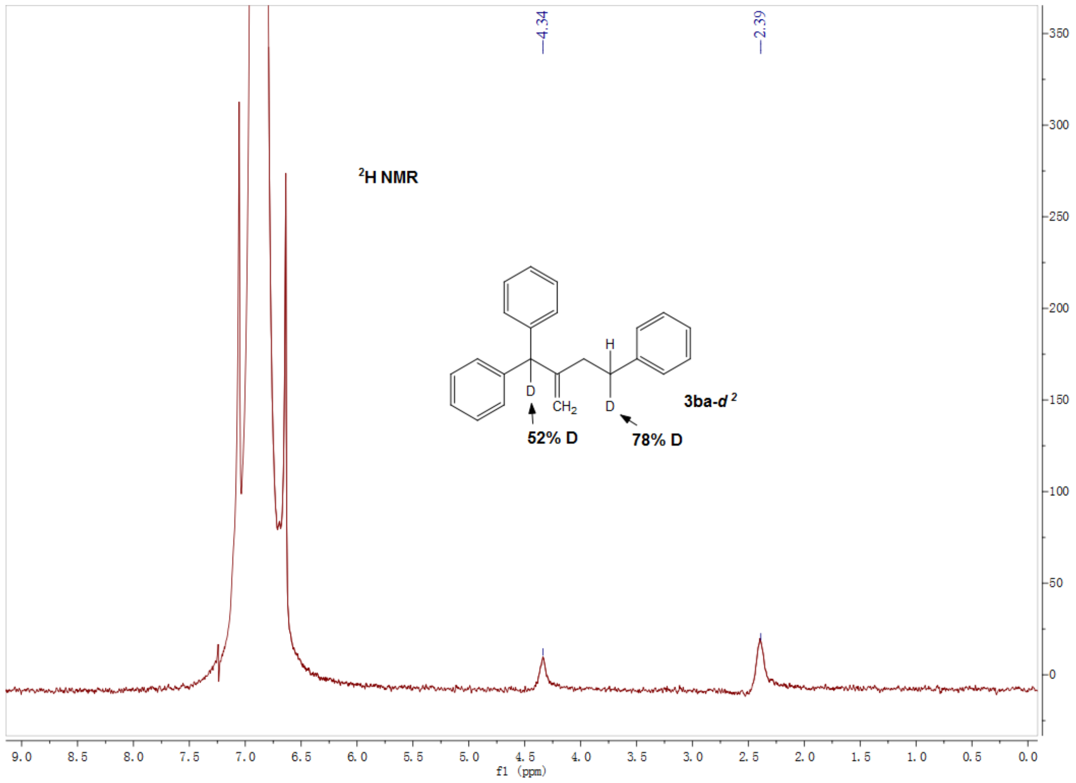

References:

(1) T. Kippo, K. Hamaoka and I. Ryu, *J. Am. Chem. Soc.*, 2013, **135**, 632-635.

(2) C.-Y. Ho and L. He, *Angew. Chem., Int. Ed.*, 2010, **49**, 9182-9186.

5. Copies of NMR spectra of products

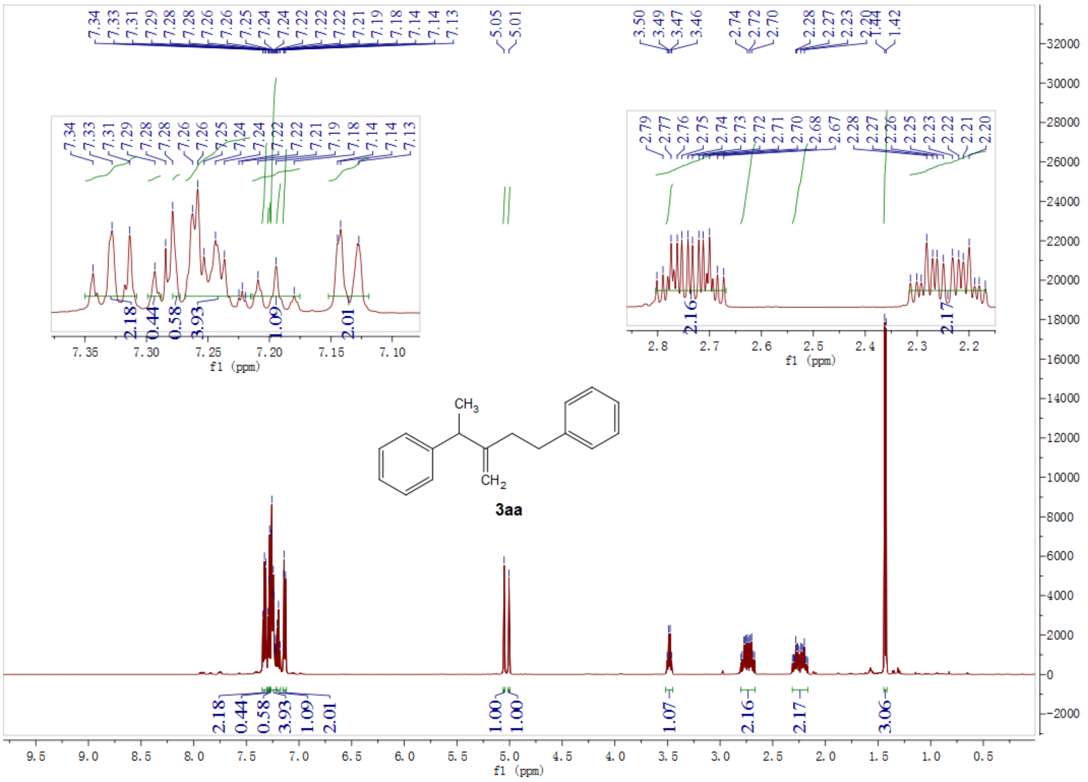

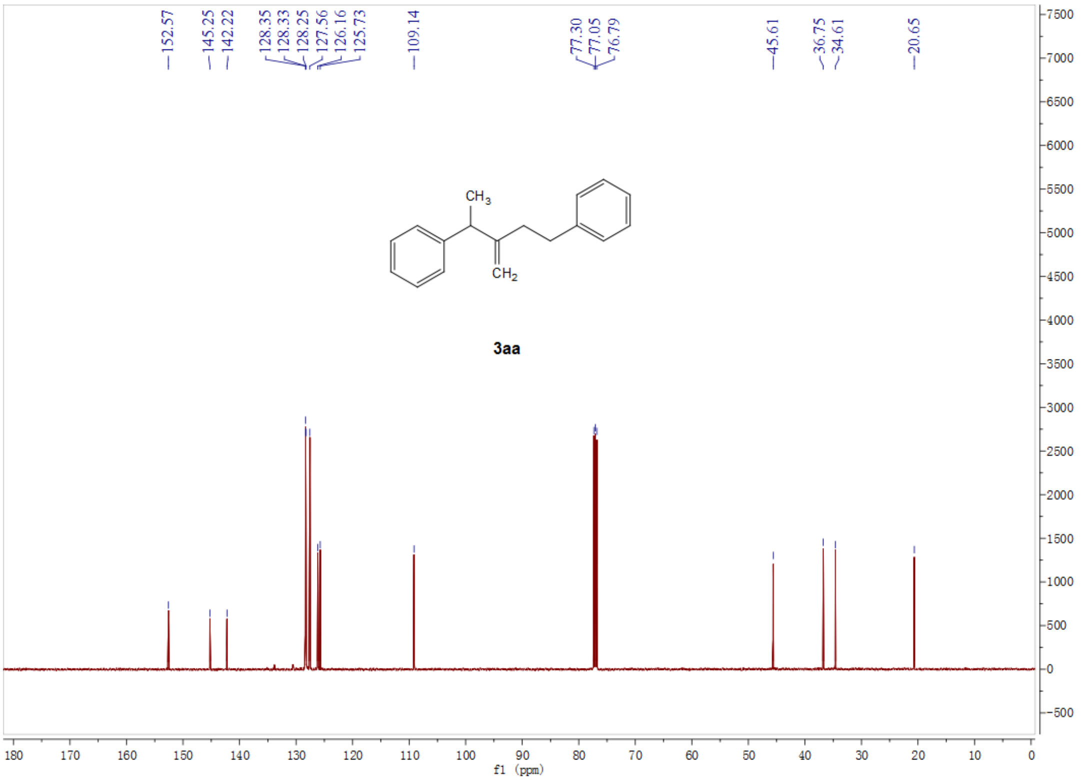

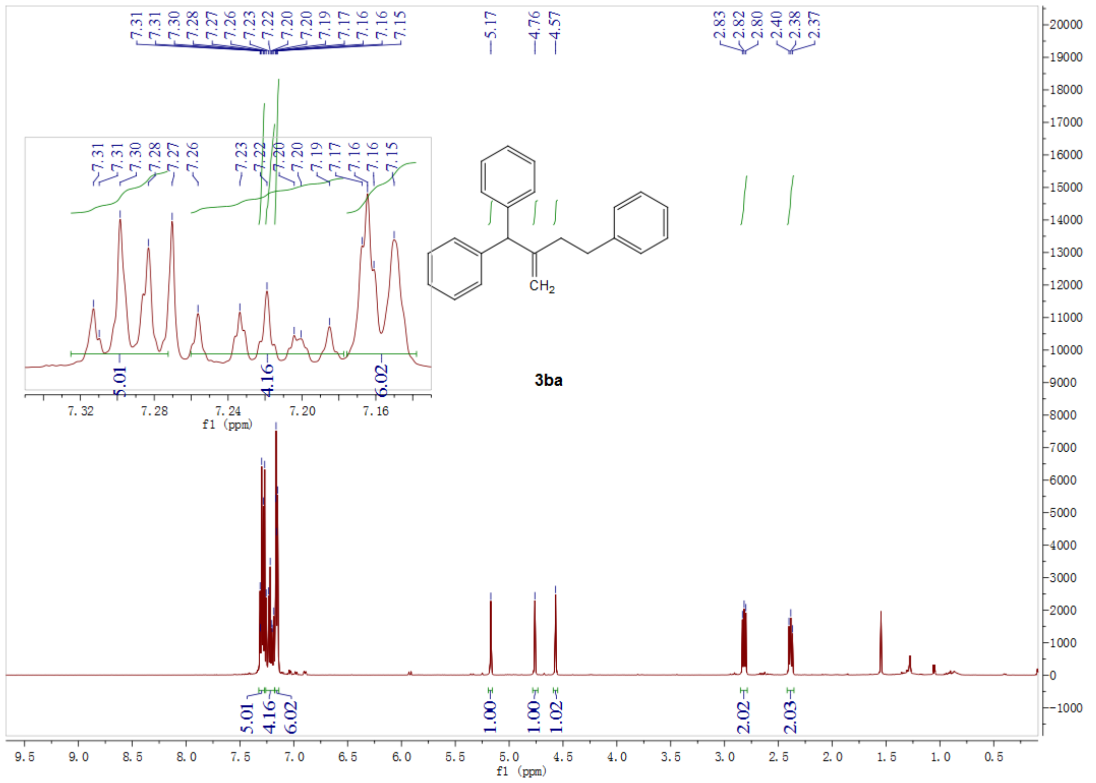
3ba
CH2
f1 (ppm)

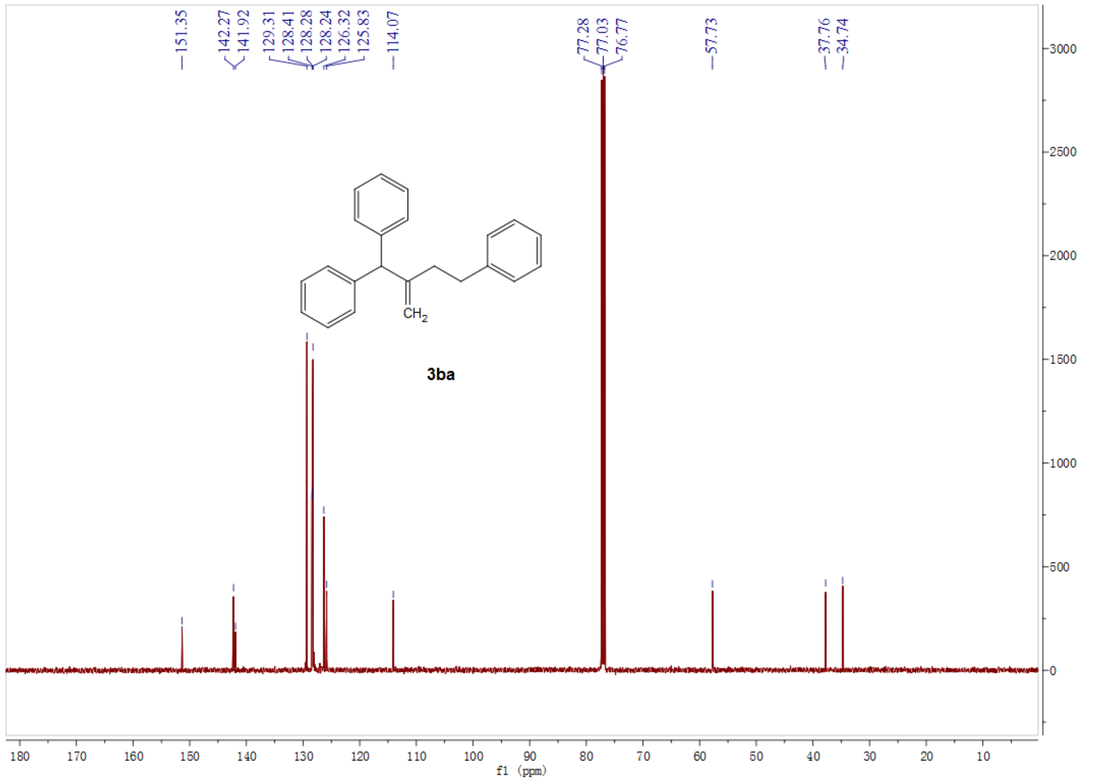
3ba
CH2
f1 (ppm)

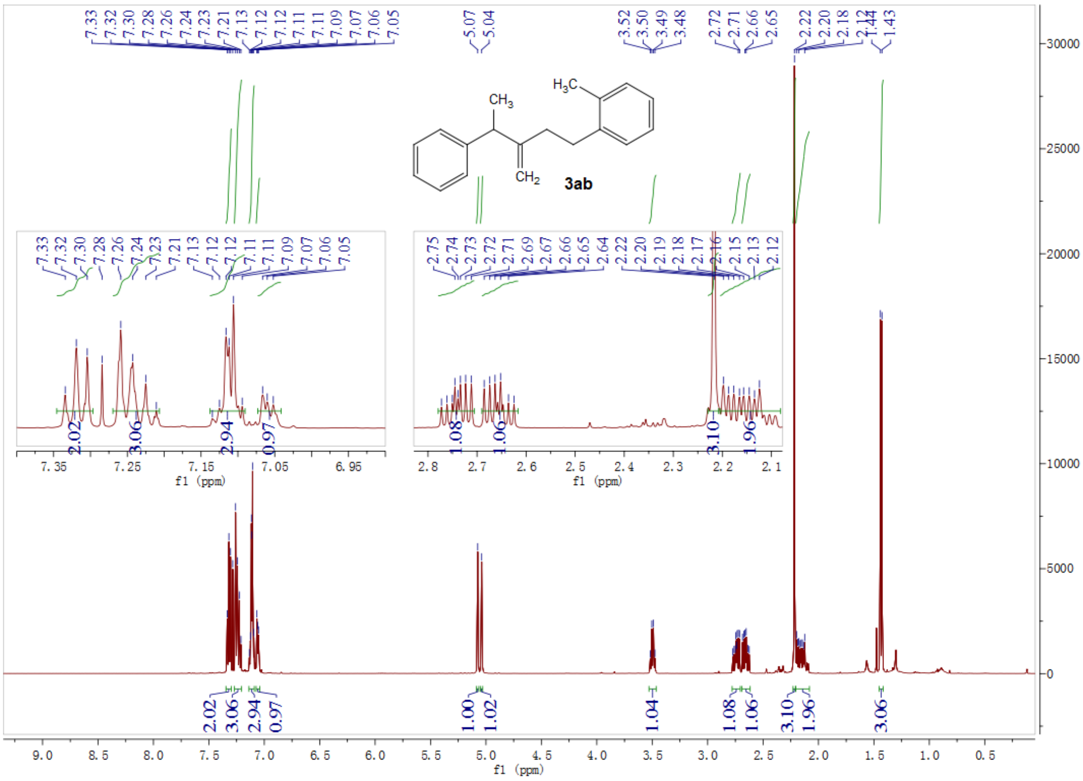
3ab
f1 (ppm)

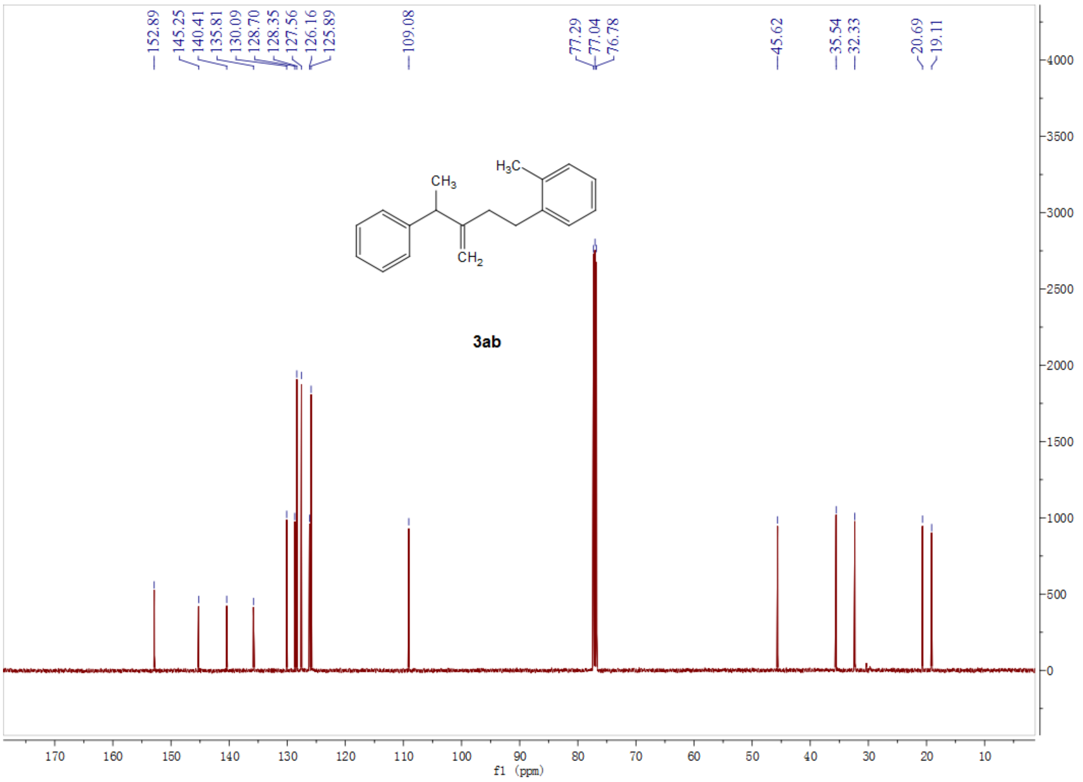
3ab
f1 (ppm)

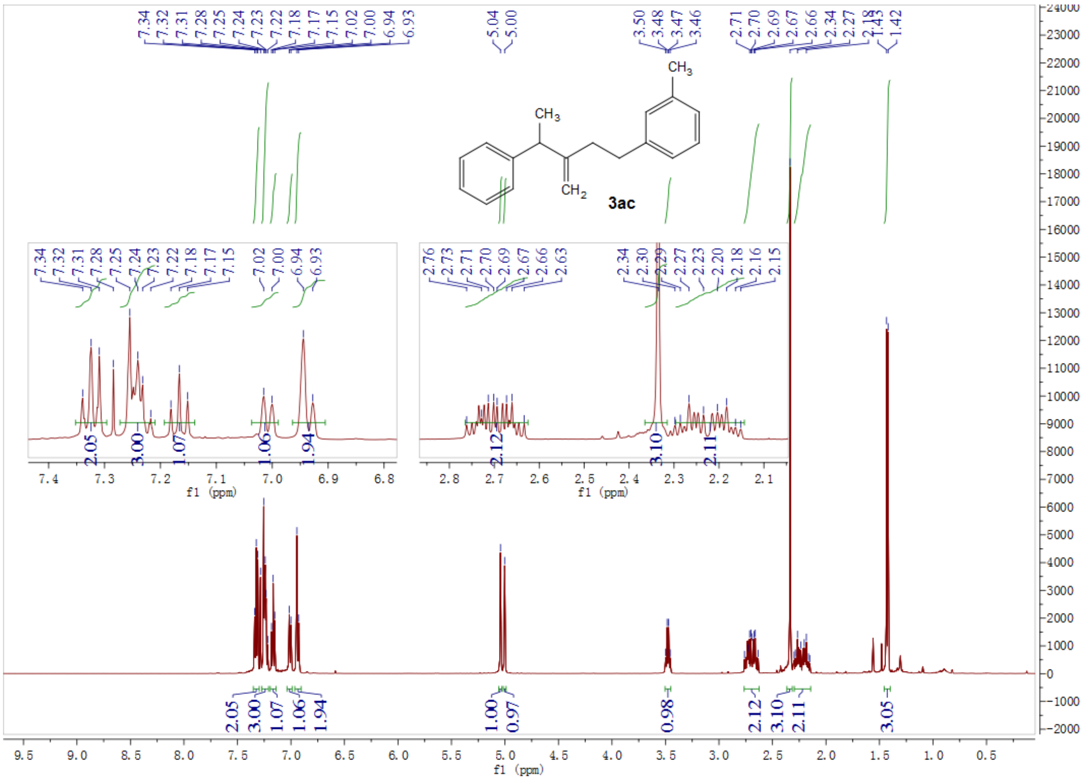
7.34 7.32 7.31 7.28 7.25 7.24 7.23 7.22 7.18 7.17 7.15 7.02 7.00 6.94 6.93
5.04 5.00
3.50 3.48 3.47 3.46
2.71 2.70 2.69 2.67 2.66 2.34 2.27 2.18 1.43 1.42
CH3
CH3
CH2
3ac
2.05 3.00 1.07 1.06 1.94
2.12 3.10 2.11
1.00 0.97 0.98 2.12 3.10 2.11 3.05
f1 (ppm)

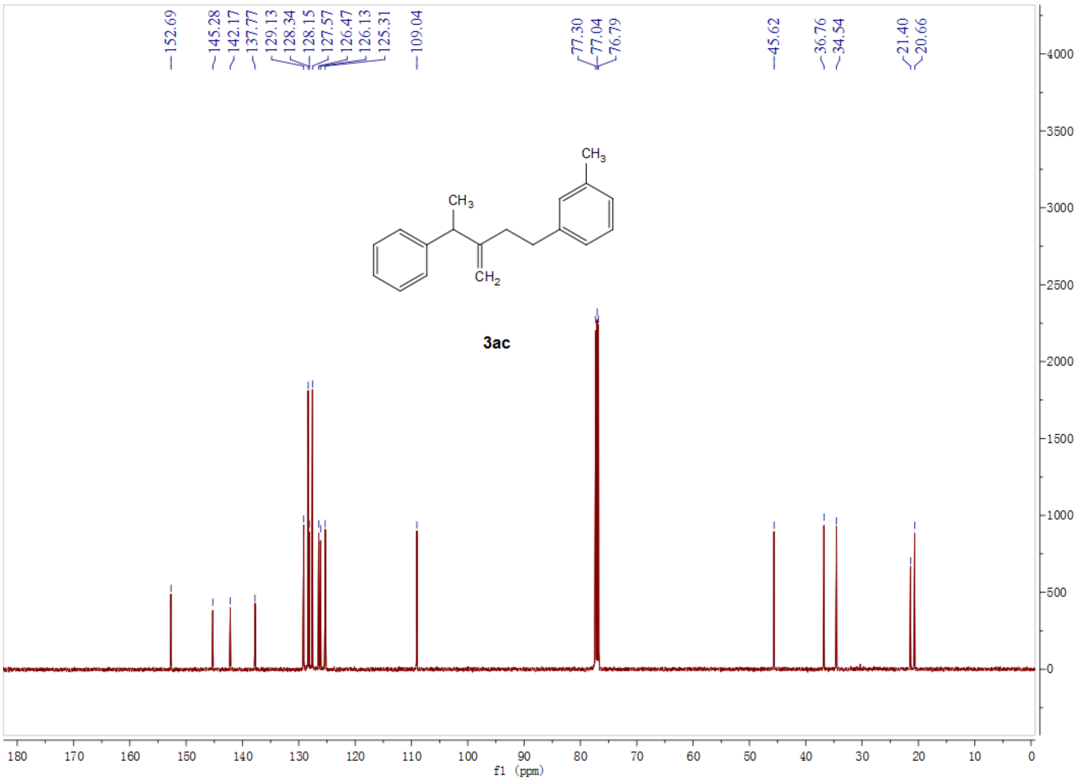
152.69
145.28 142.17 137.77 129.13 128.34 128.15 127.57 126.47 126.13 125.31
109.04
77.30 77.04 76.79
45.62
36.76 34.54
21.40 20.66
CH3
CH3
CH2
3ac
f1 (ppm)

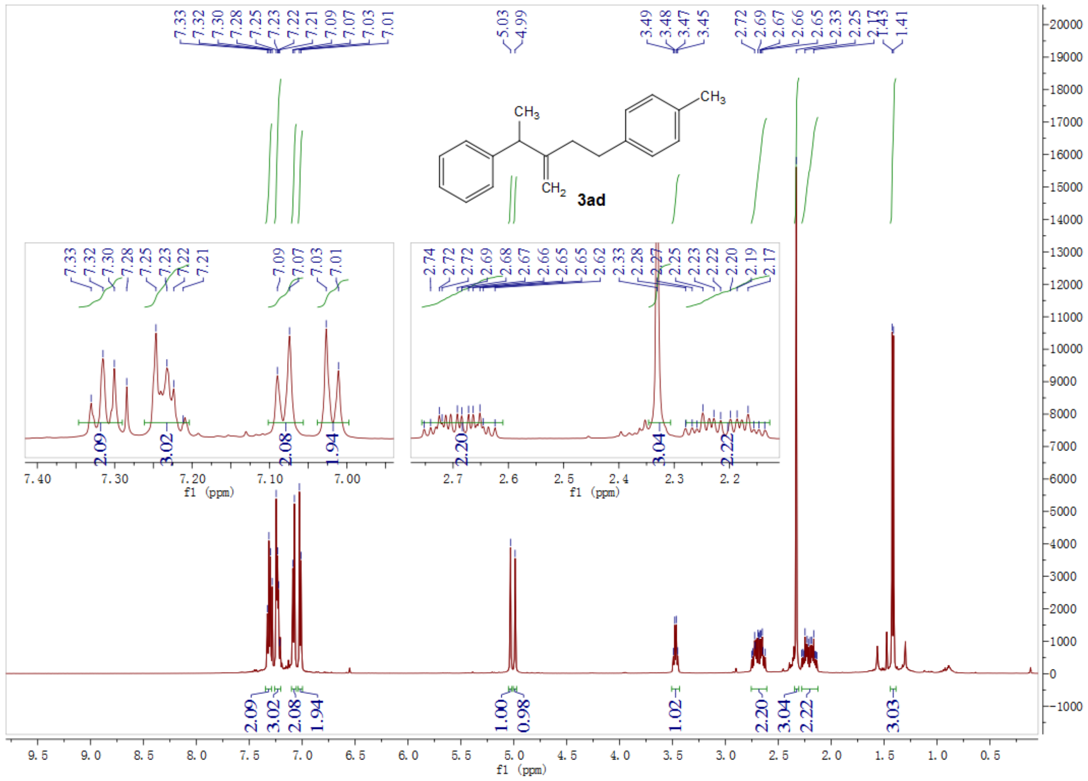
7.33 7.32 7.30 7.28 7.25 7.23 7.22 7.21 7.09 7.07 7.03 7.01
5.03 4.99
3.49 3.48 3.47 3.45
2.72 2.69 2.67 2.66 2.65 2.33 2.25 2.17 1.43 1.41
CH3
CH2
3ad
2.09 3.02 2.08 1.94
1.00 0.98
1.02
2.20 3.04 2.22
3.03
f1 (ppm)

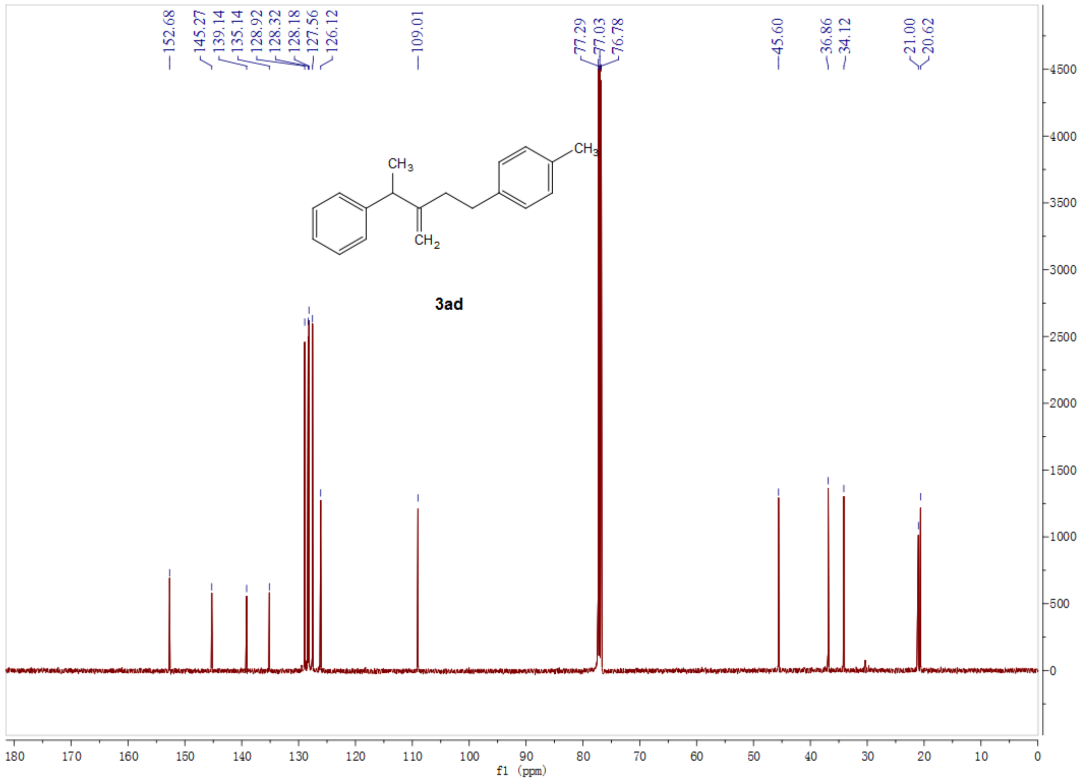
152.68 145.27 139.14 135.14 128.92 128.32 128.18 127.56 126.12
109.01
77.29 77.03 76.78
45.60
36.86 34.12
21.00 20.62
CH3
CH2
3ad
f1 (ppm)

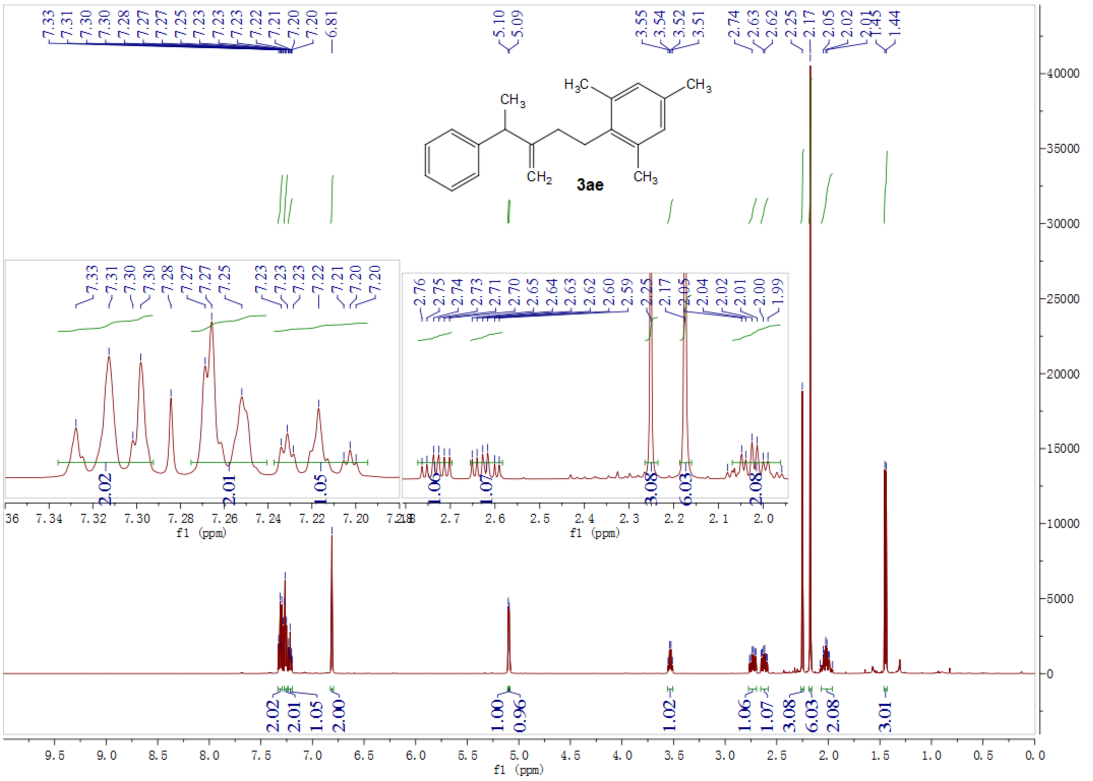
3ae
f1 (ppm)

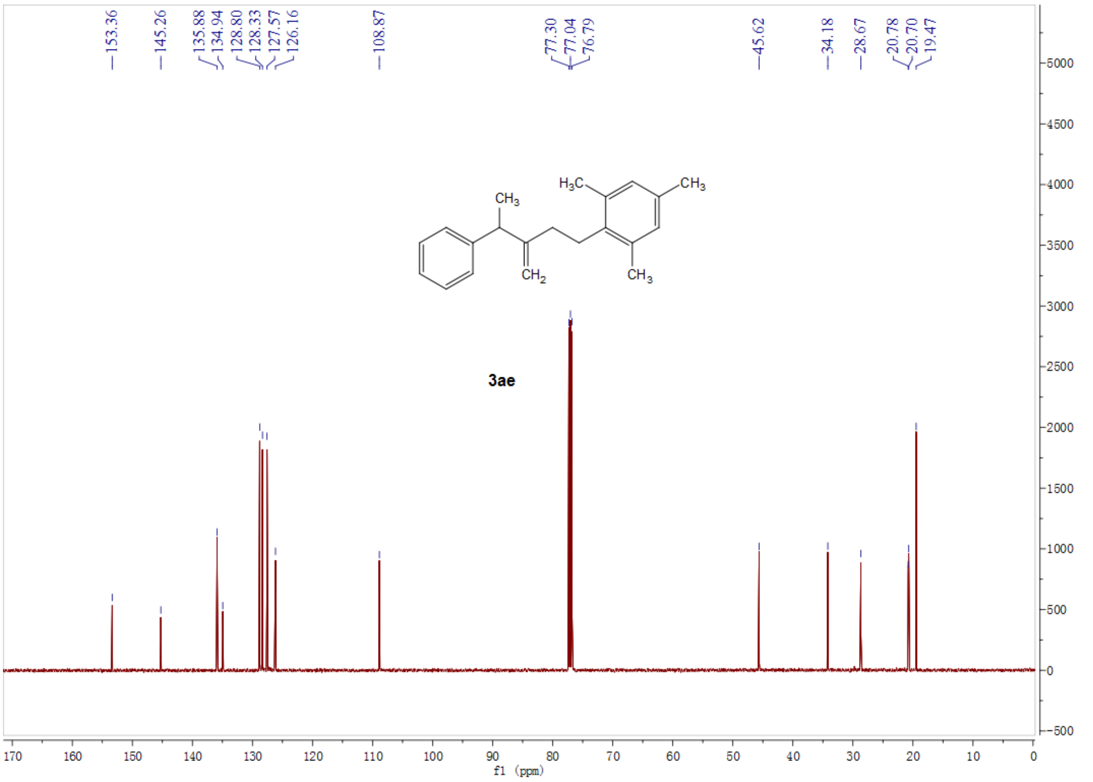
3ae
f1 (ppm)

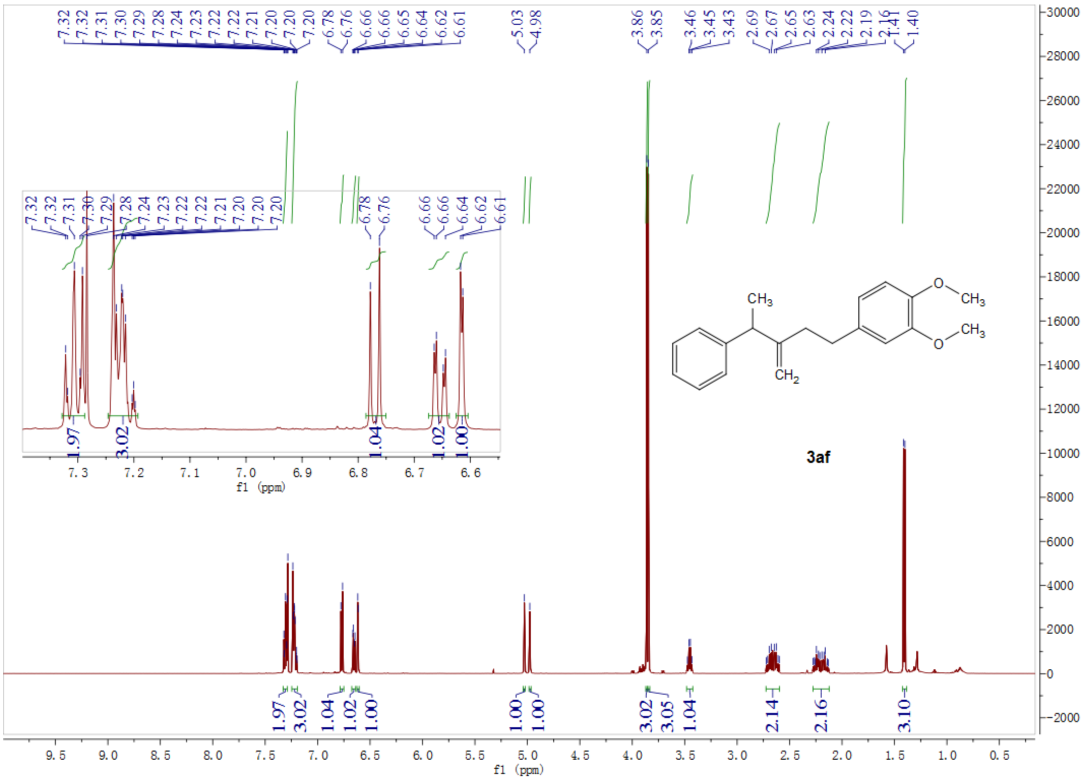
3af
f1 (ppm)

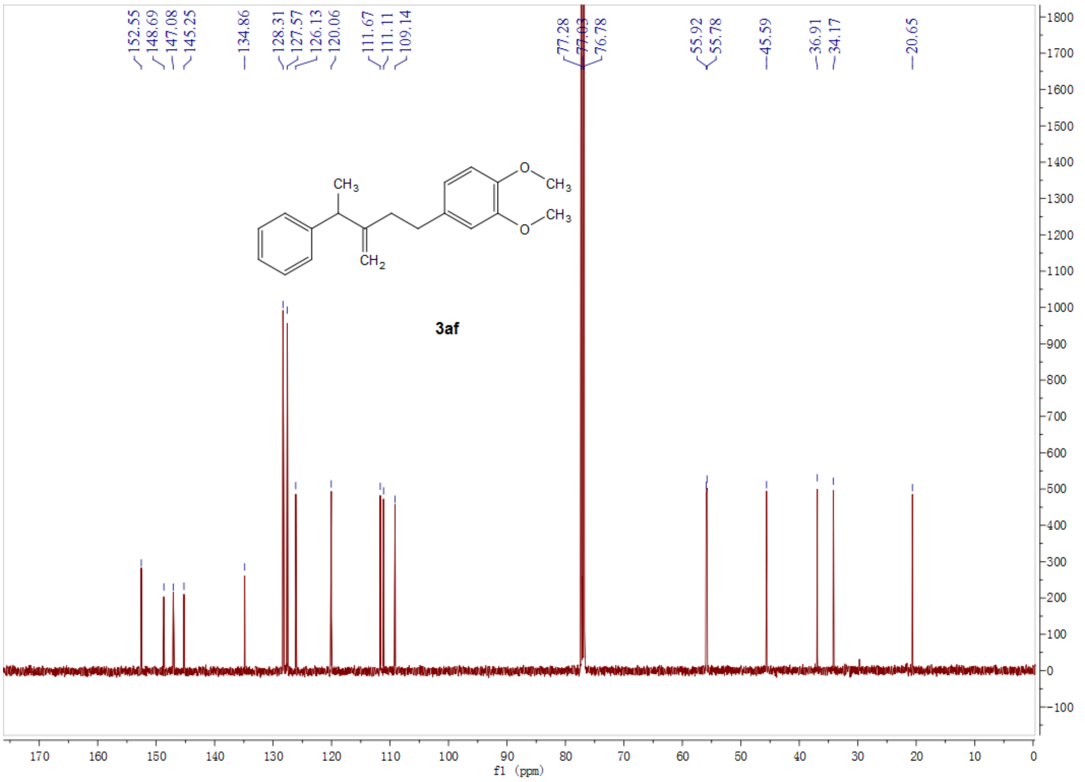
3af
f1 (ppm)

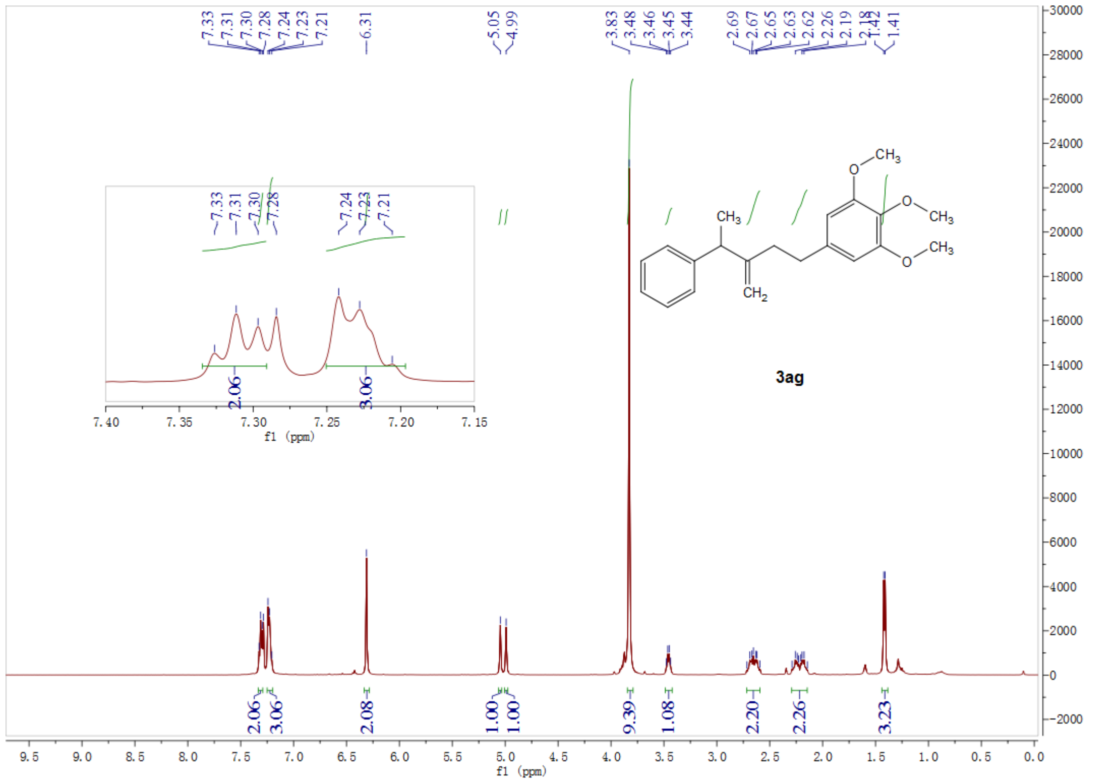

7.33
7.31
7.30
7.28
7.24
7.23
7.21
6.31
5.05
4.99
3.83
3.48
3.46
3.45
3.44
2.69
2.67
2.65
2.63
2.62
2.26
2.19
2.18
1.42
1.41
CH3
CH2
3ag
2.06
3.06
2.08
1.00
1.00
9.39
1.08
2.20
2.26
3.23
f1 (ppm)

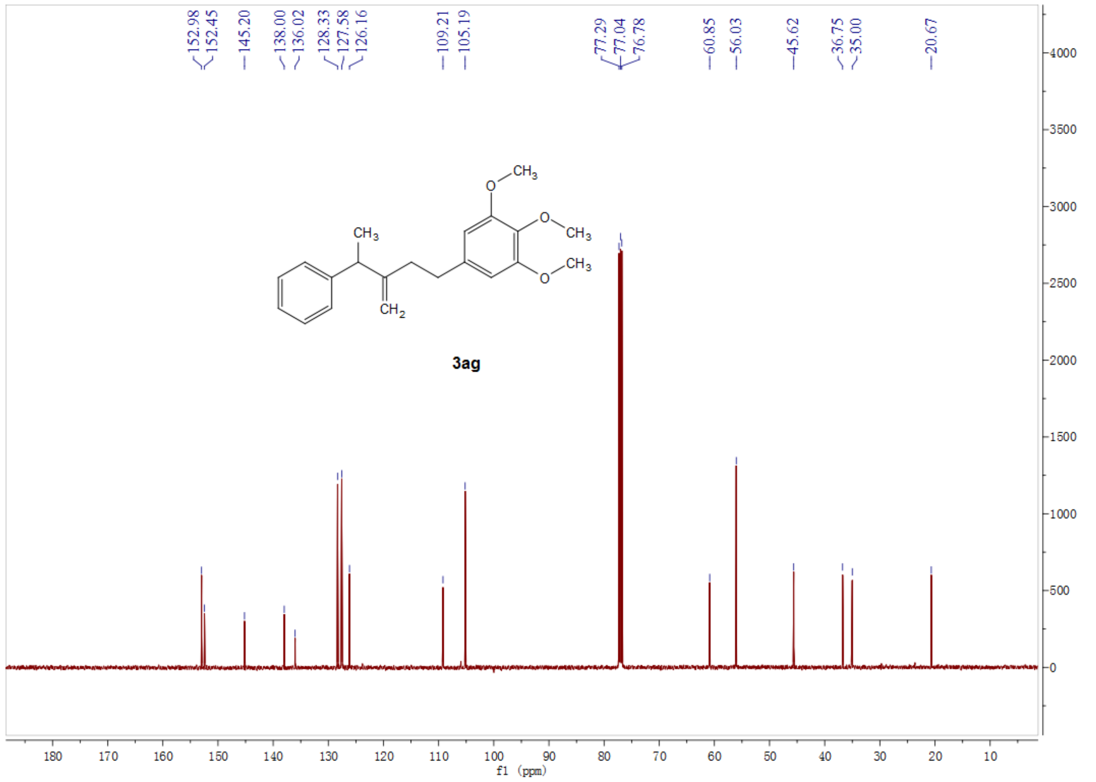

152.98
152.45
145.20
138.00
136.02
128.33
127.58
126.16
109.21
105.19
77.29
77.04
76.78
60.85
56.03
45.62
36.75
35.00
20.67
CH3
CH2
3ag
f1 (ppm)

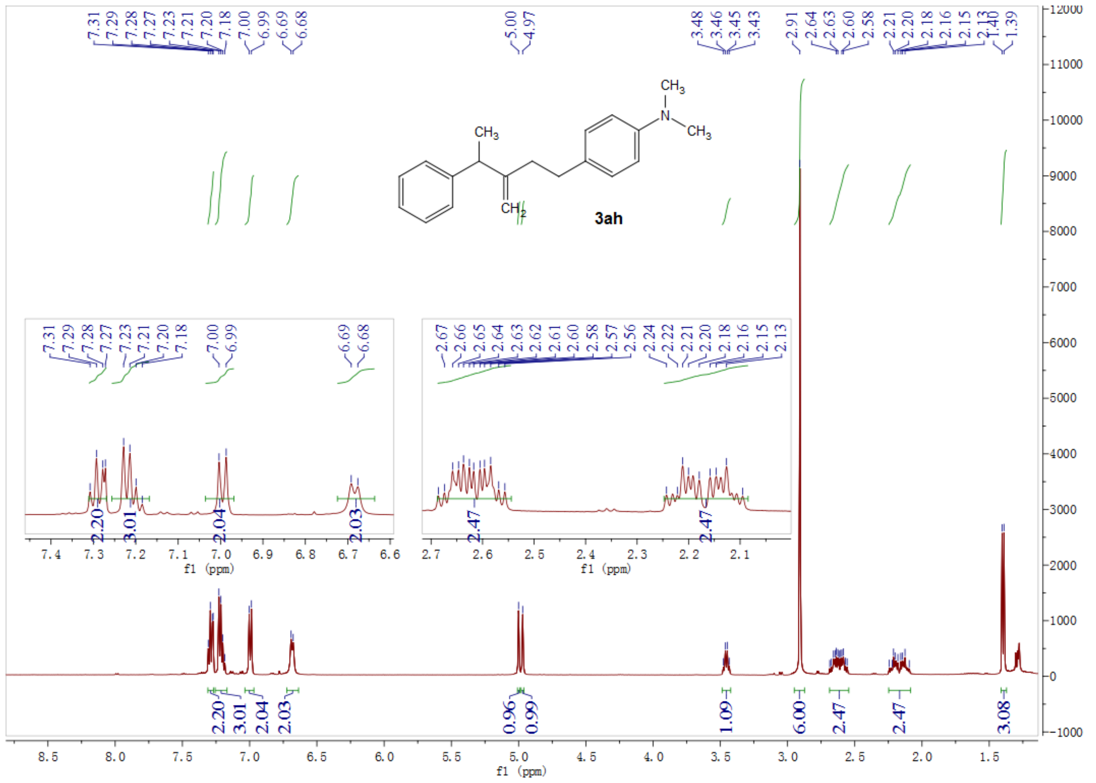
3ah

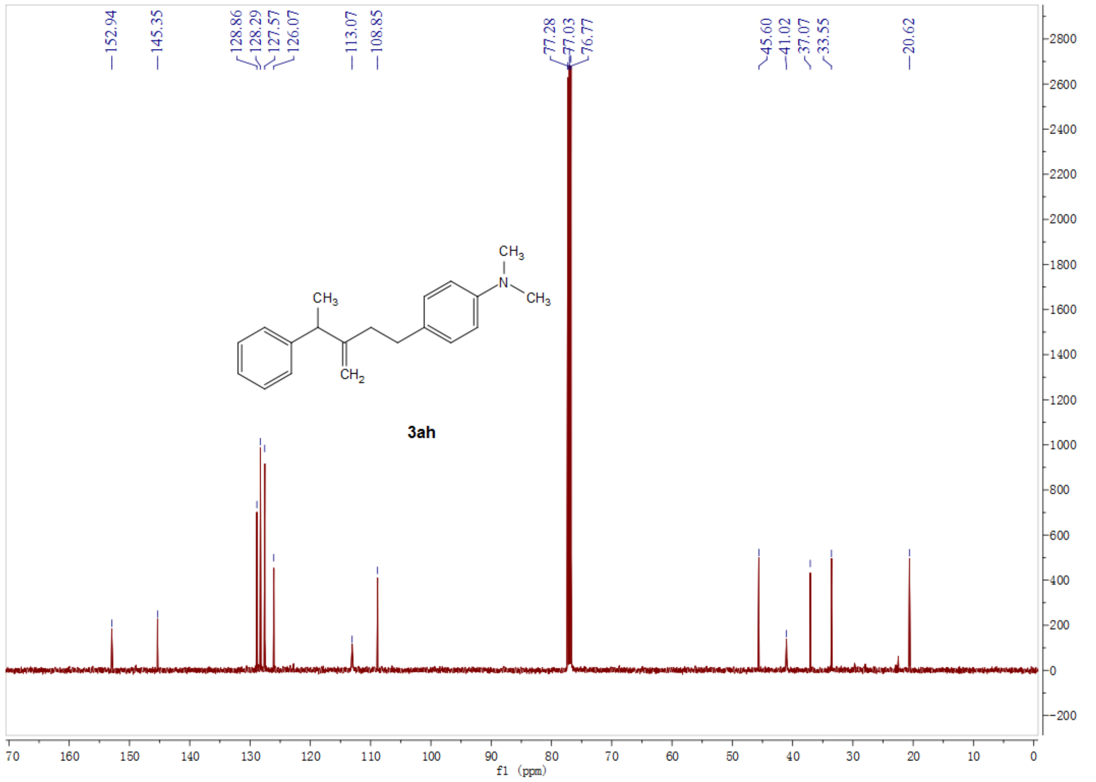
3ah

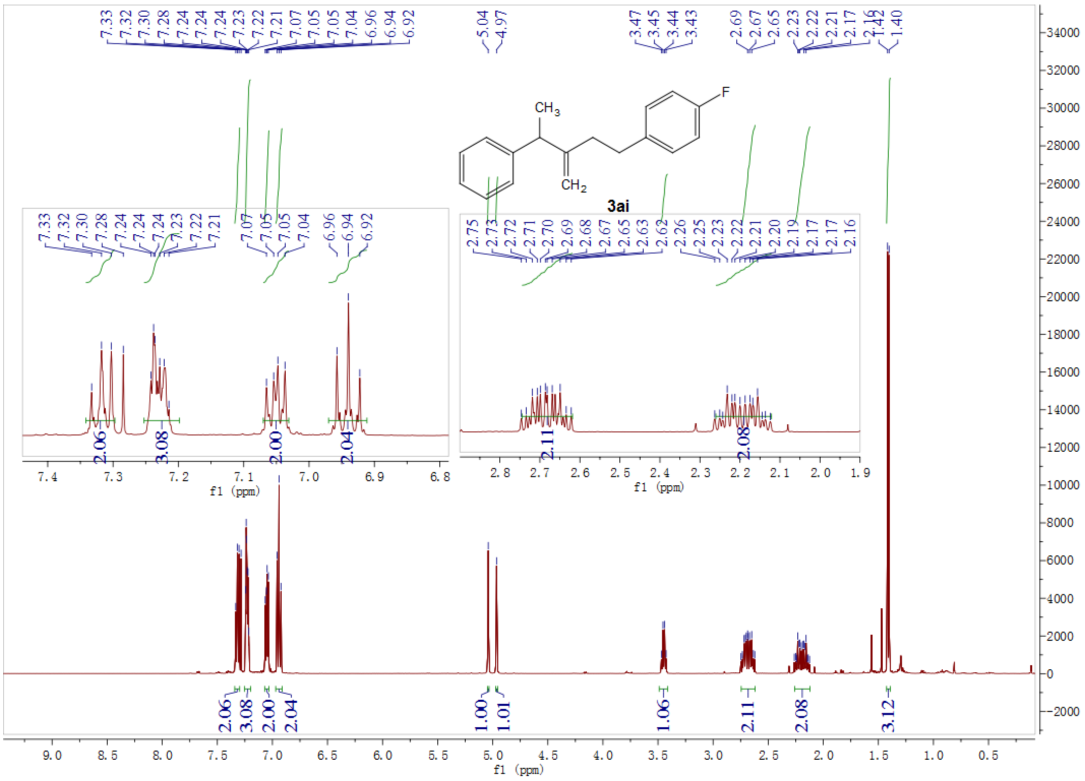
7.33 7.32 7.30 7.28 7.24 7.24 7.24 7.23 7.22 7.21 7.07 7.05 7.05 7.04 6.96 6.94 6.92
5.04 4.97
3.47 3.45 3.44 3.43
2.69 2.67 2.65 2.23 2.22 2.21 2.17 2.16 1.42 1.40
CH3
CH2
F
3ai
2.06 3.08 2.00 2.04 1.00 1.01 1.06 2.11 2.08 3.12
f1 (ppm)

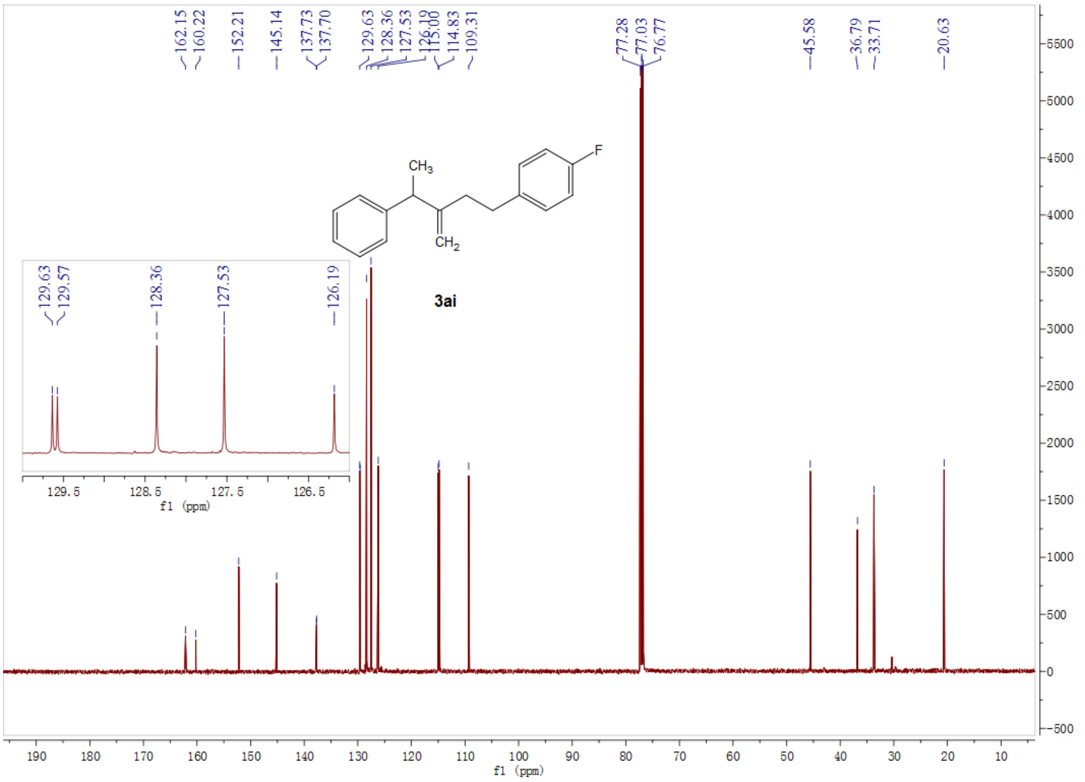
162.15 160.22 152.21 145.14 137.73 137.70 129.63 128.36 127.53 126.19 115.00 114.83 109.31 77.28 77.03 76.77 45.58 36.79 33.71 20.63
CH3
CH2
F
3ai
129.63 129.57 128.36 127.53 126.19
f1 (ppm)

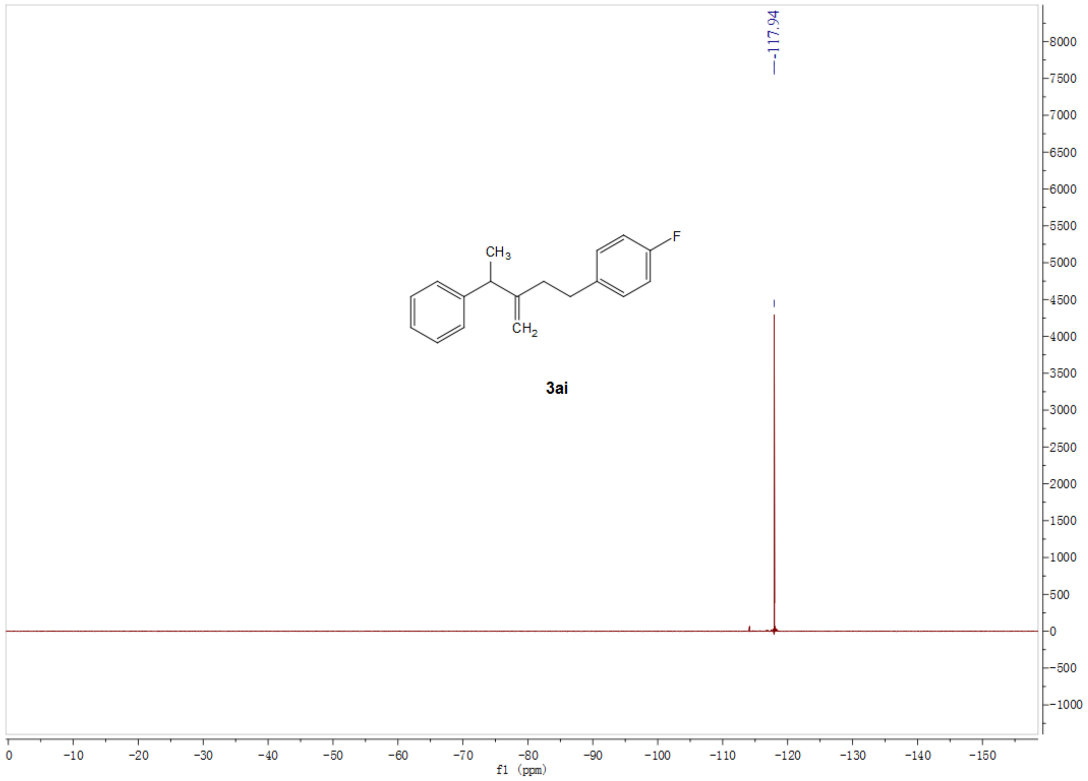

-117.94
CH3
F
CH2
3ai
8000
7500
7000
6500
6000
5500
5000
4500
4000
3500
3000
2500
2000
1500
1000
500
0
-500
-1000
0
-10
-20
-30
-40
-50
-60
-70
-80
-90
-100
-110
-120
-130
-140
-150
f1 (ppm)

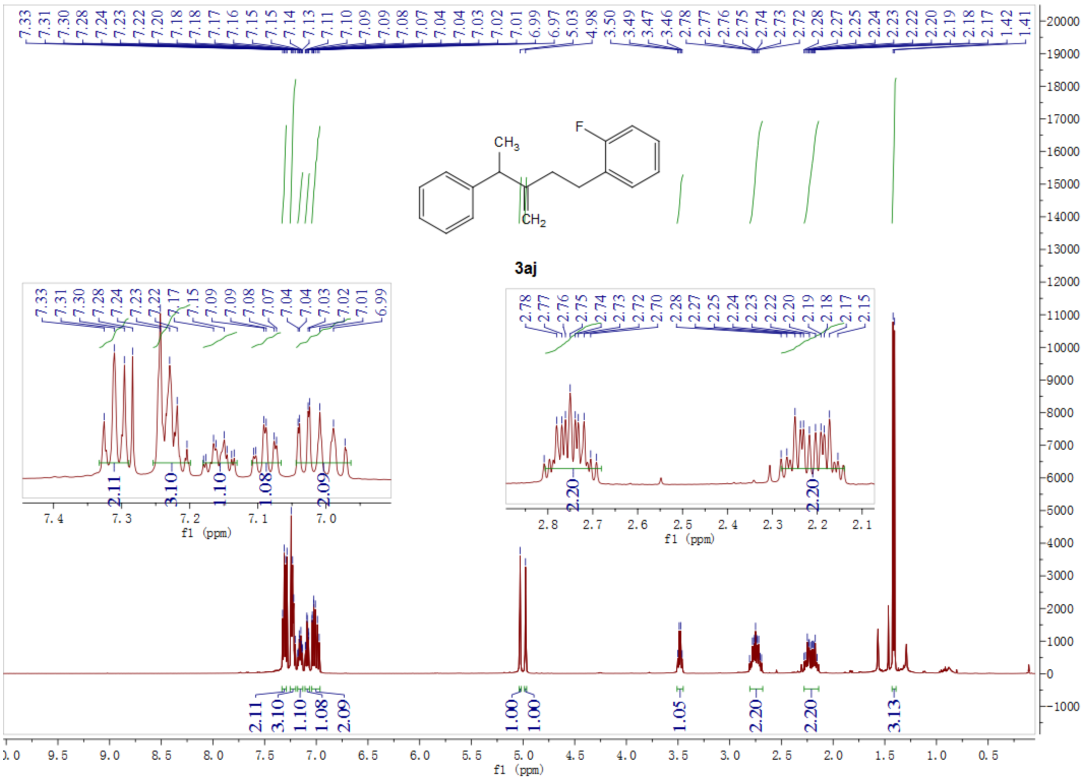

7.33 7.31 7.30 7.28 7.24 7.23 7.22 7.20 7.18 7.18 7.17 7.16 7.15 7.15 7.14 7.13 7.11 7.10 7.09 7.09 7.08 7.07 7.04 7.04 7.03 7.02 7.01 6.99 6.97 5.03 4.98 3.50 3.49 3.47 3.46 2.78 2.77 2.76 2.75 2.74 2.73 2.72 2.28 2.27 2.25 2.24 2.23 2.22 2.20 2.19 2.18 2.17 1.42 1.41
CH3
F
CH2
3aj
2.11 3.10 1.10 1.08 2.09 1.00 1.00 1.05 2.20 2.20 3.13
f1 (ppm)

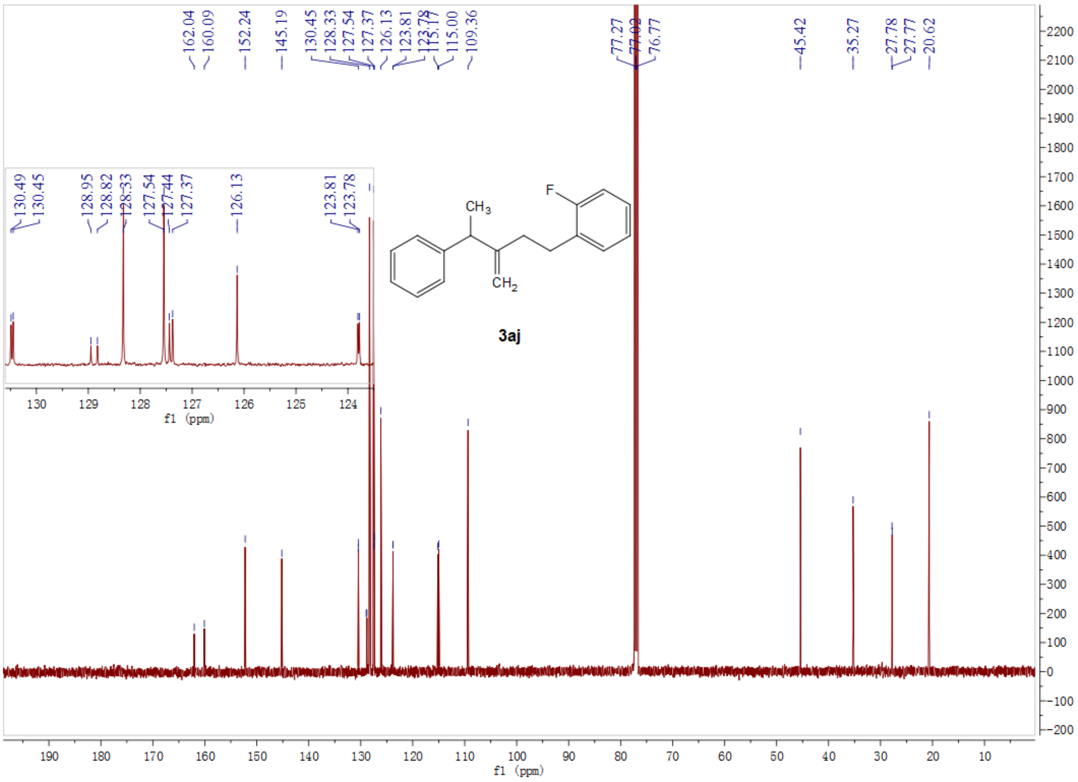

162.04 160.09 152.24 145.19 130.45 128.33 127.54 127.37 126.13 123.81 123.78 115.17 115.00 109.36 77.27 77.02 76.77 45.42 35.27 27.78 27.77 20.62
130.49 130.45 128.95 128.82 128.33 127.54 127.44 127.37 126.13 123.81 123.78
CH3
F
CH2
3aj
f1 (ppm)

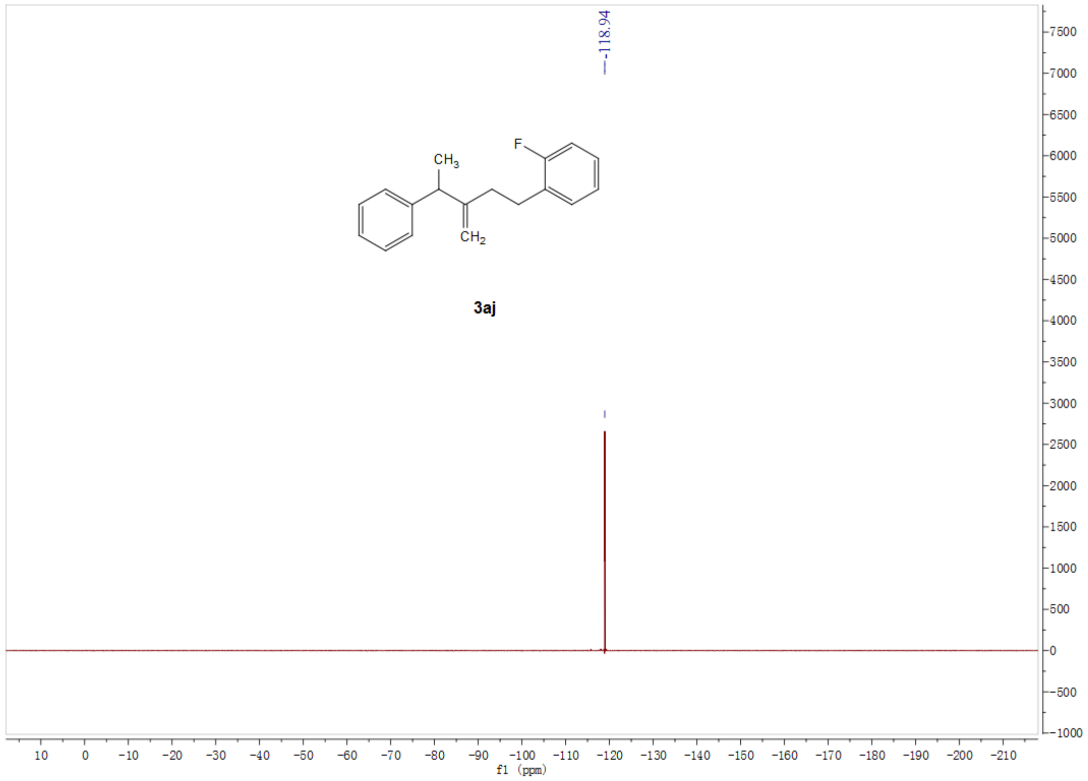

-118.94
CH3
F
CH2
3aj
7500
7000
6500
6000
5500
5000
4500
4000
3500
3000
2500
2000
1500
1000
500
0
-500
-1000
10 0 -10 -20 -30 -40 -50 -60 -70 -80 -90 -100 -110 -120 -130 -140 -150 -160 -170 -180 -190 -200 -210
f1 (ppm)

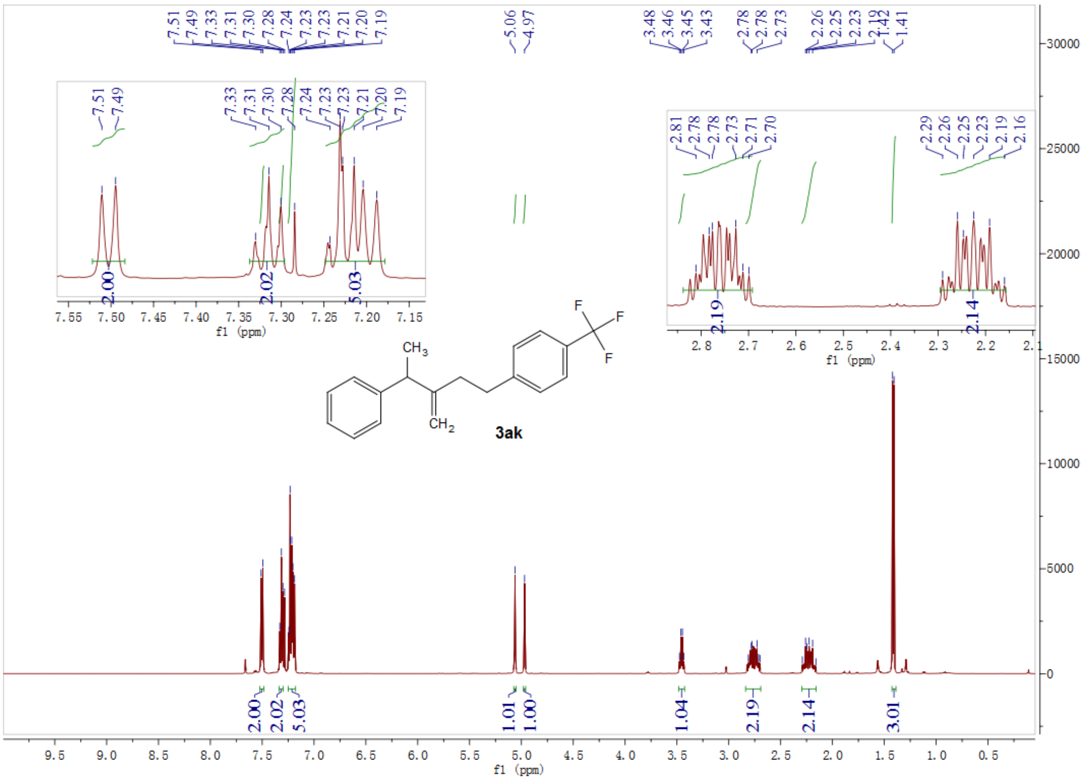

CH3
CH2
F
F
F
3ak
f1 (ppm)

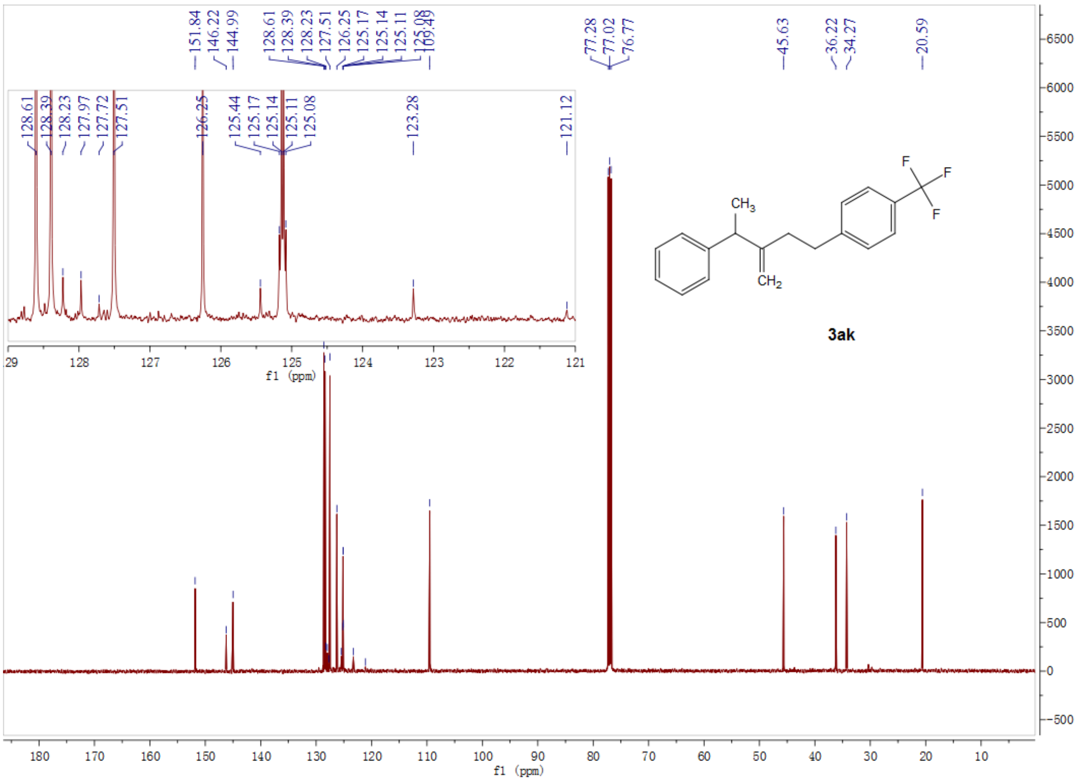

CH3
CH2
F
F
F
3ak
f1 (ppm)

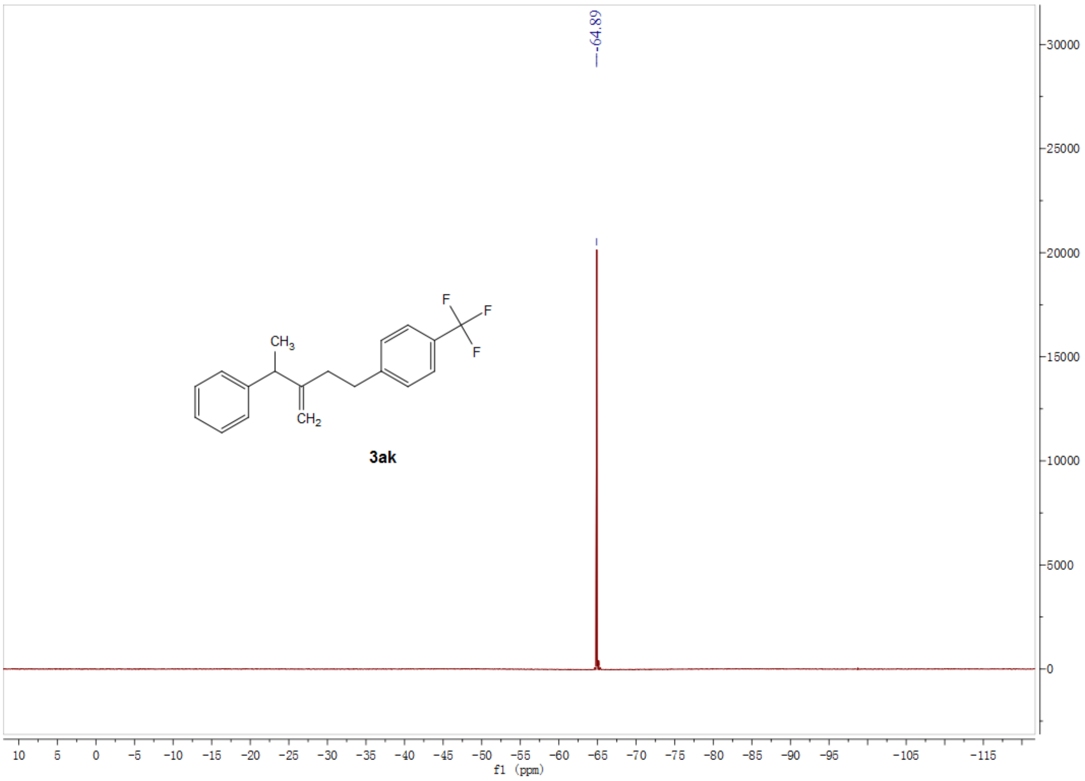

—64.89
CH3
CH2
F
F
F
3ak
30000
25000
20000
15000
10000
5000
0
10 5 0 -5 -10 -15 -20 -25 -30 -35 -40 -45 -50 -55 -60 -65 -70 -75 -80 -85 -90 -95 -105 -115
f1 (ppm)

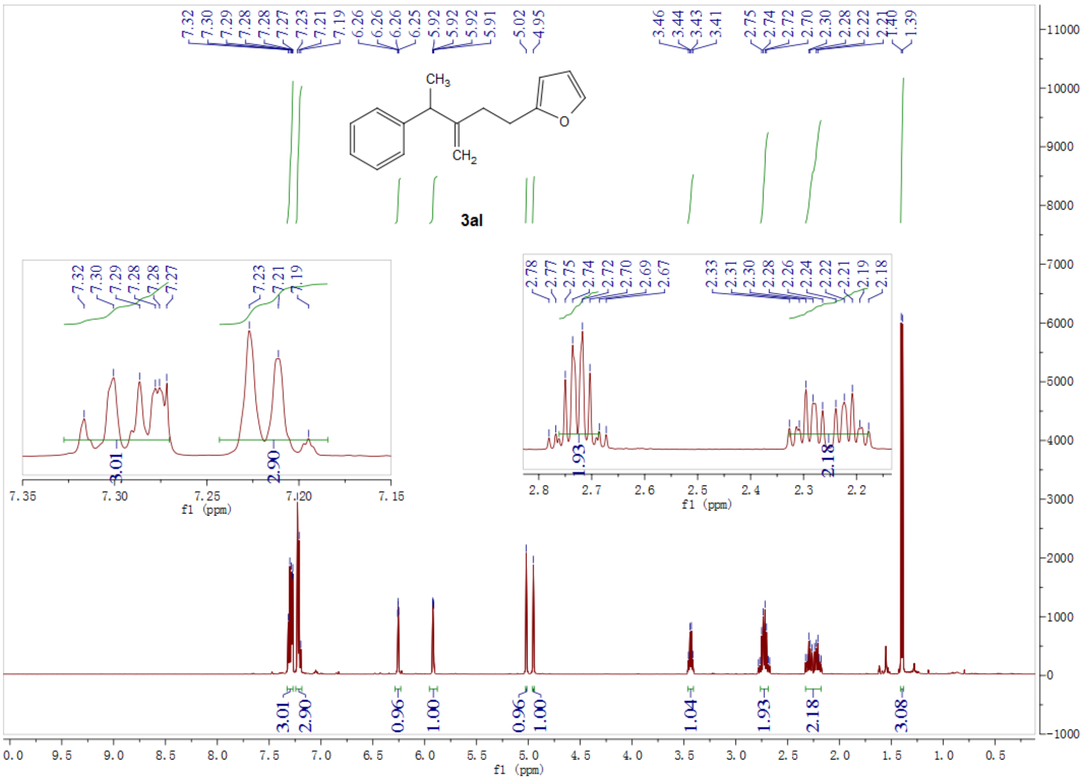

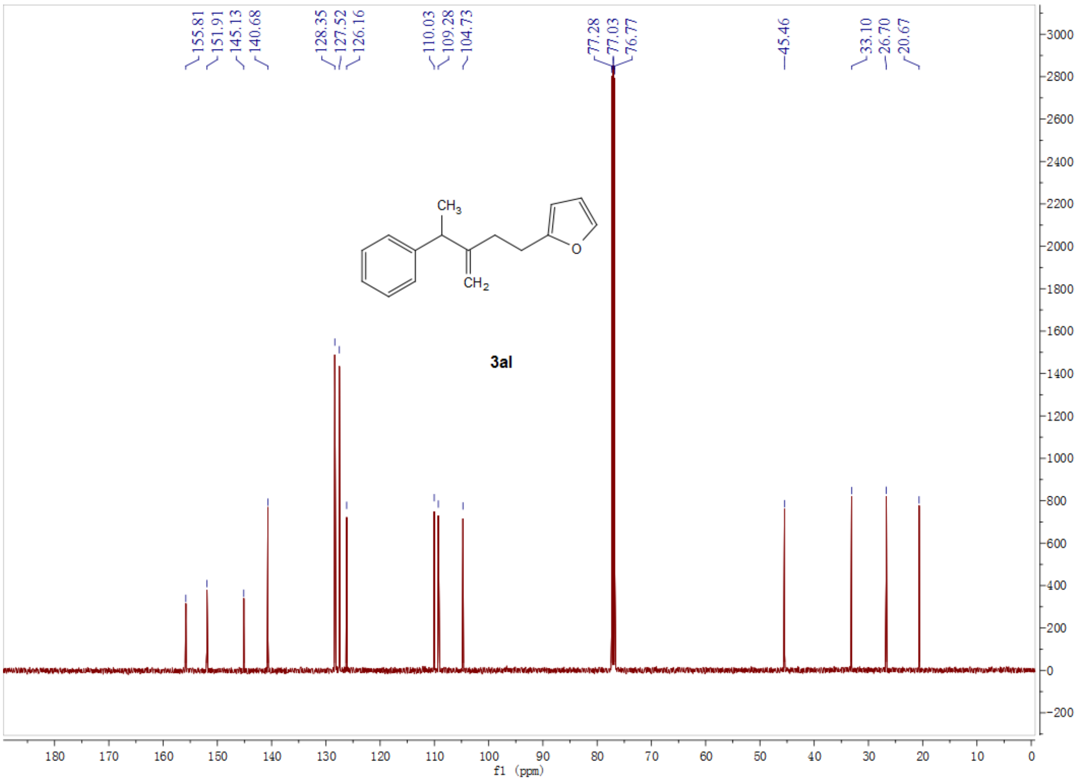

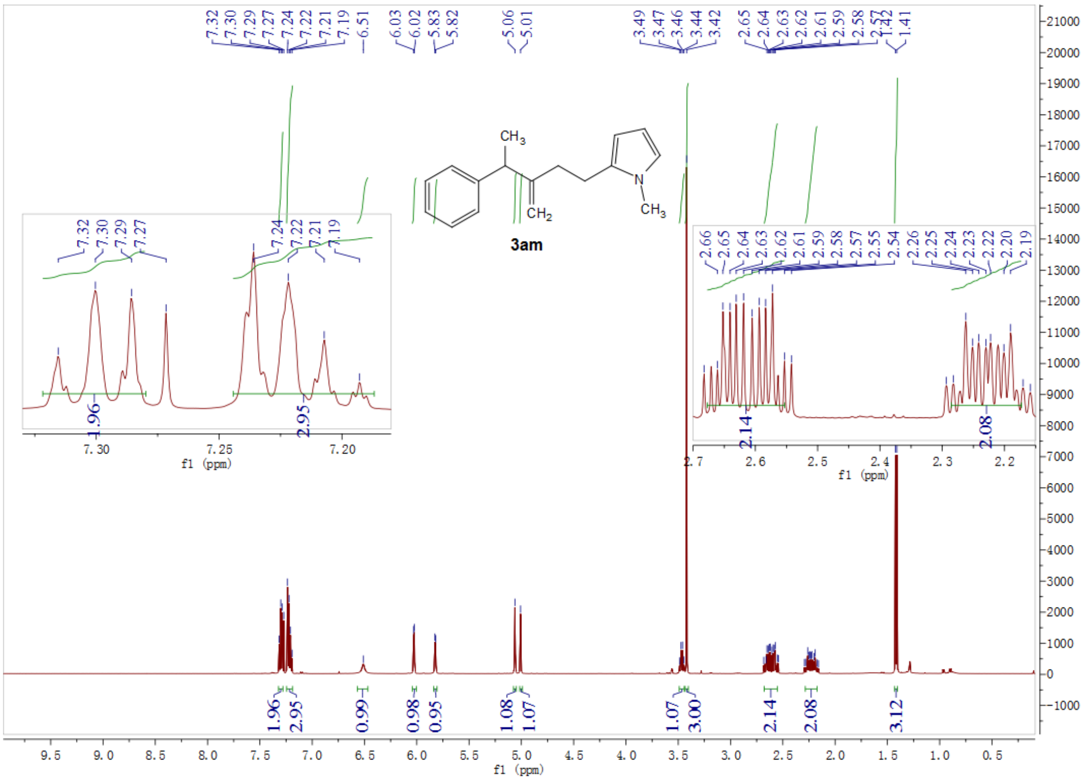

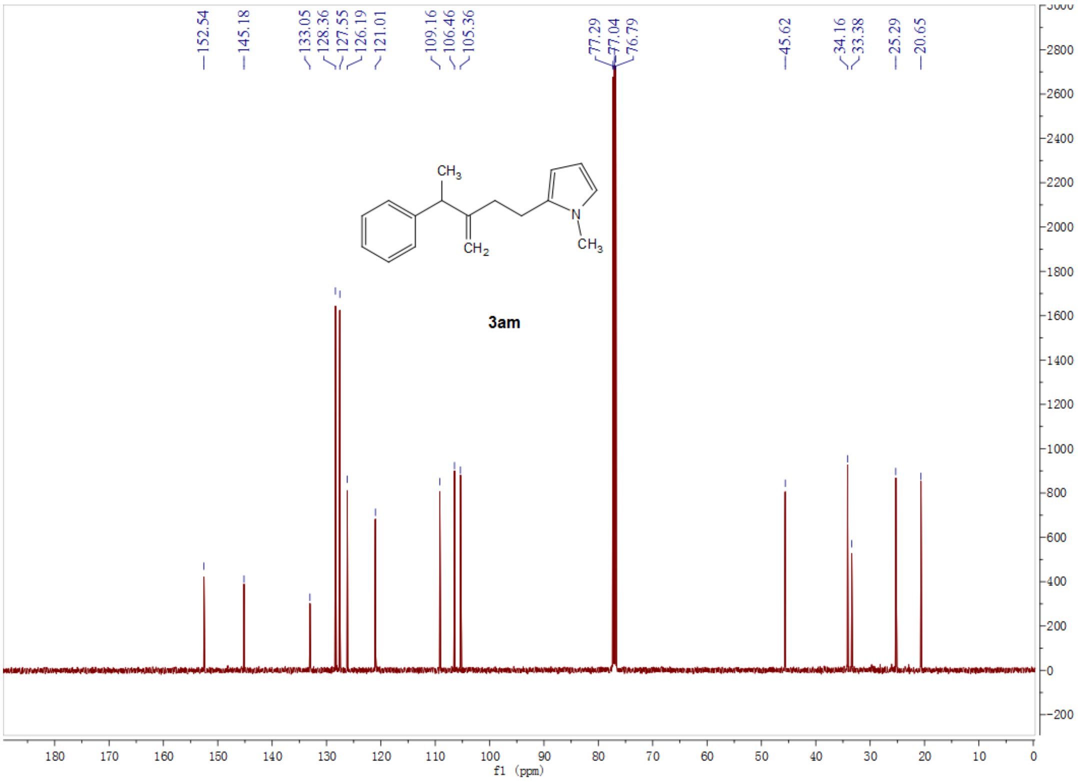

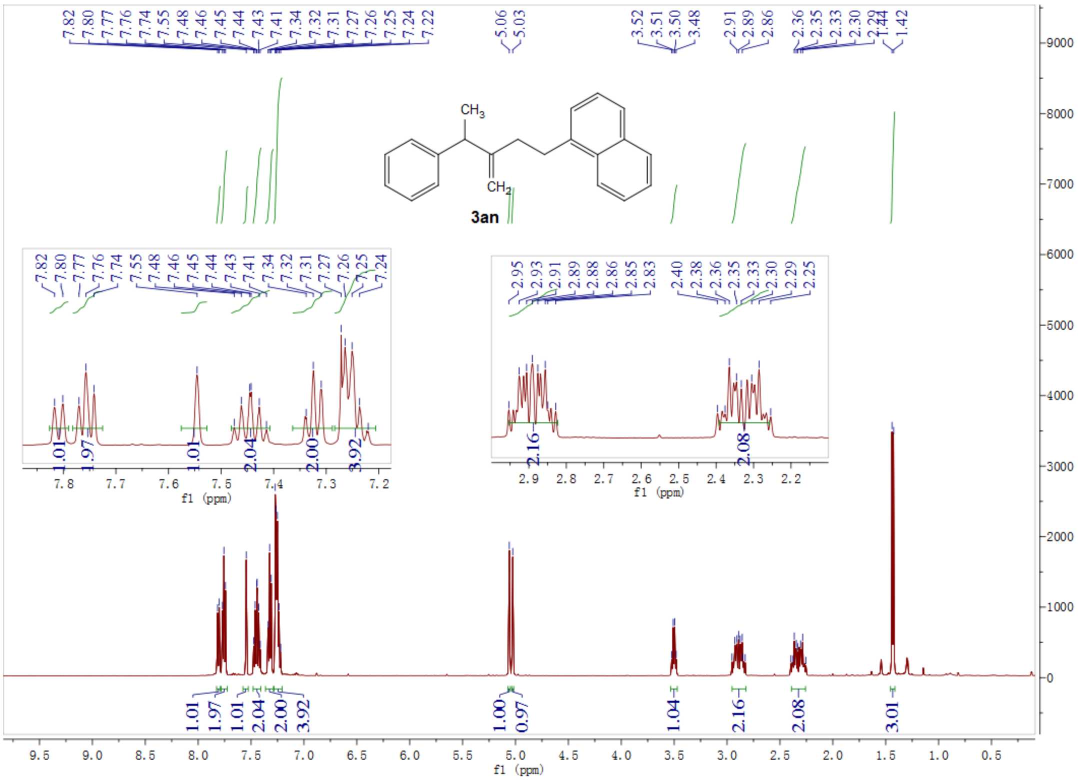
3an
f1 (ppm)

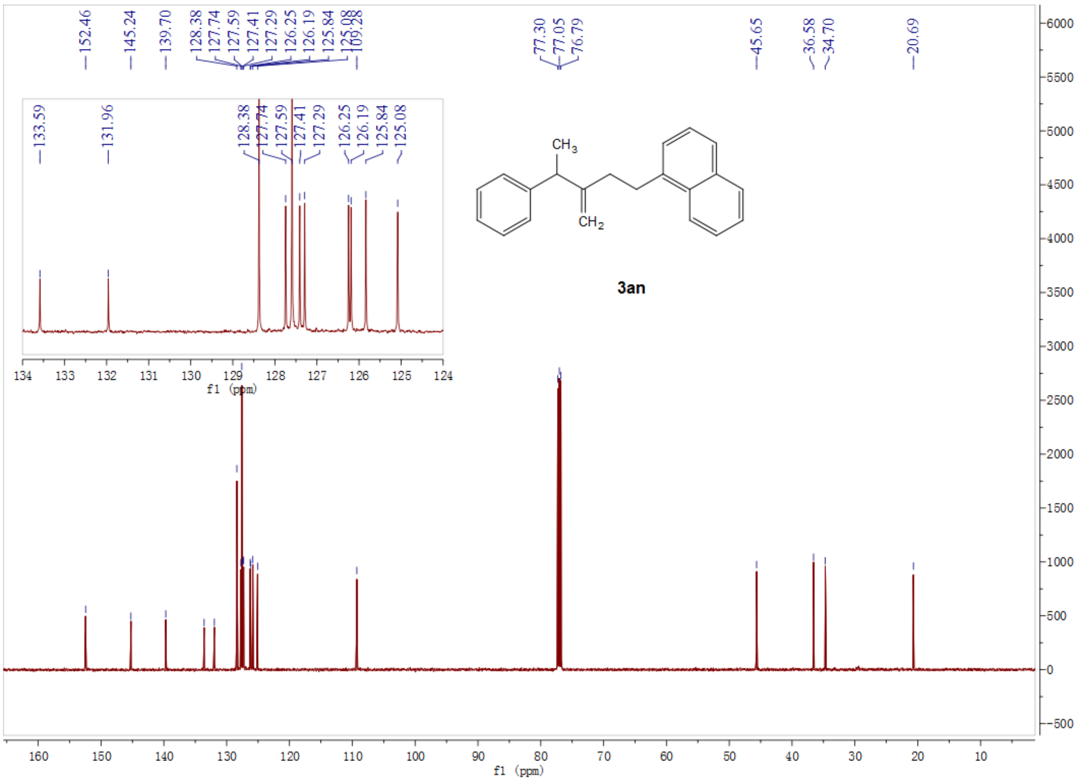
3an
f1 (ppm)

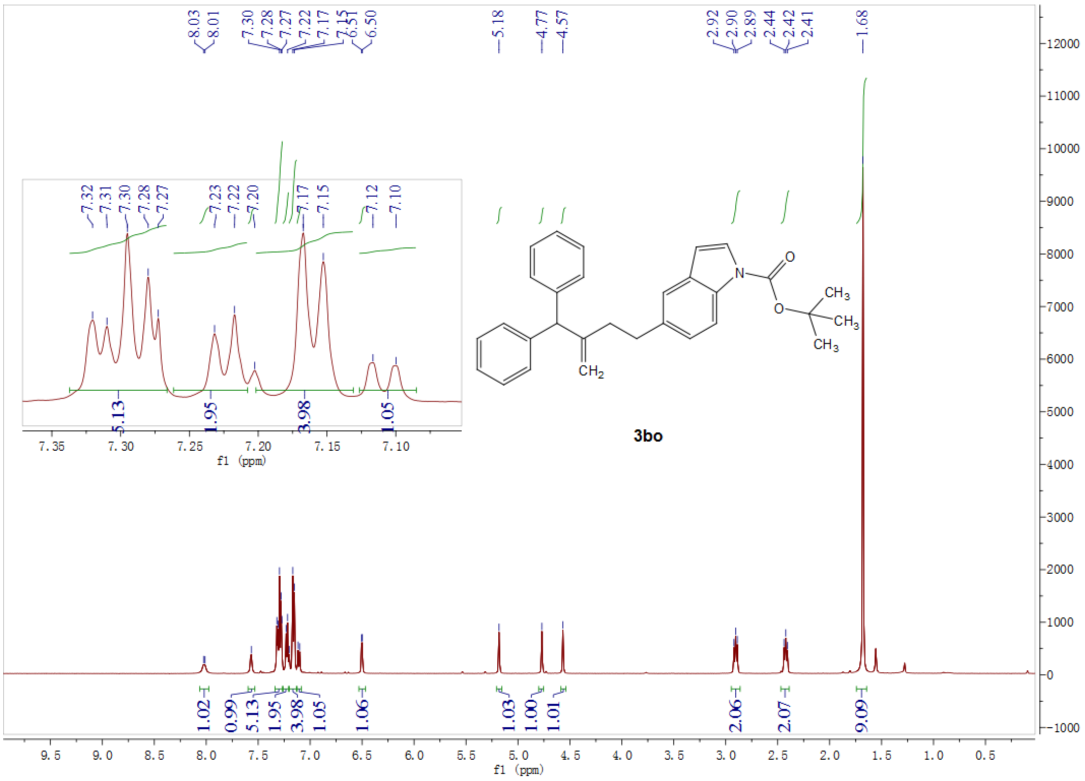

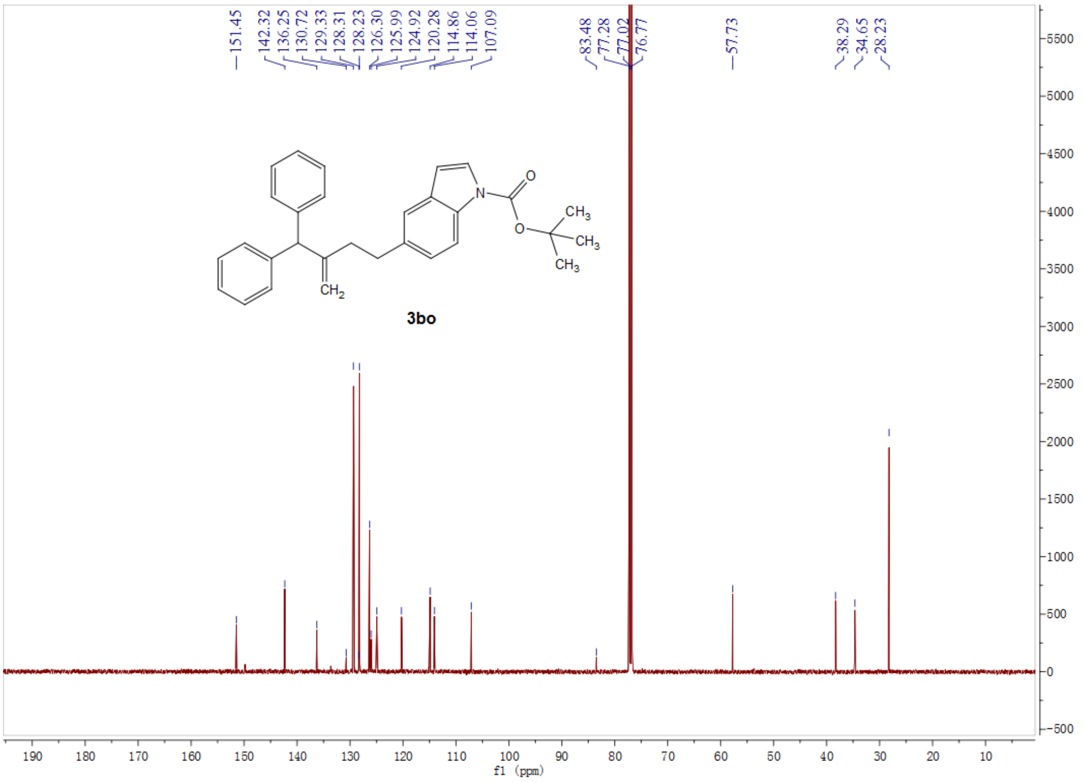

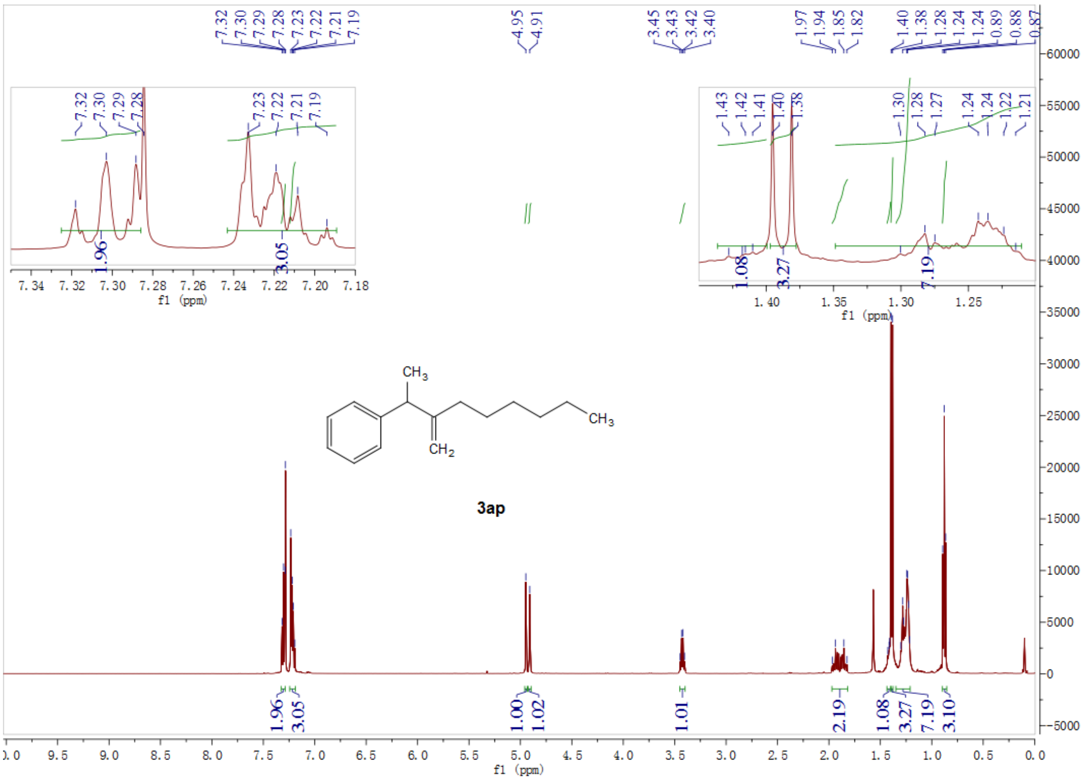
7.32 7.30 7.29 7.28 7.23 7.22 7.21 7.19
4.95 4.91
3.45 3.43 3.42 3.40
1.97 1.94 1.85 1.82
1.40 1.38 1.28 1.24 1.24 0.89 0.88 0.87
CH3
CH2
3ap
1.96 3.05 1.00 1.02 1.01 2.19 1.08 3.27 7.19 3.10
f1 (ppm)

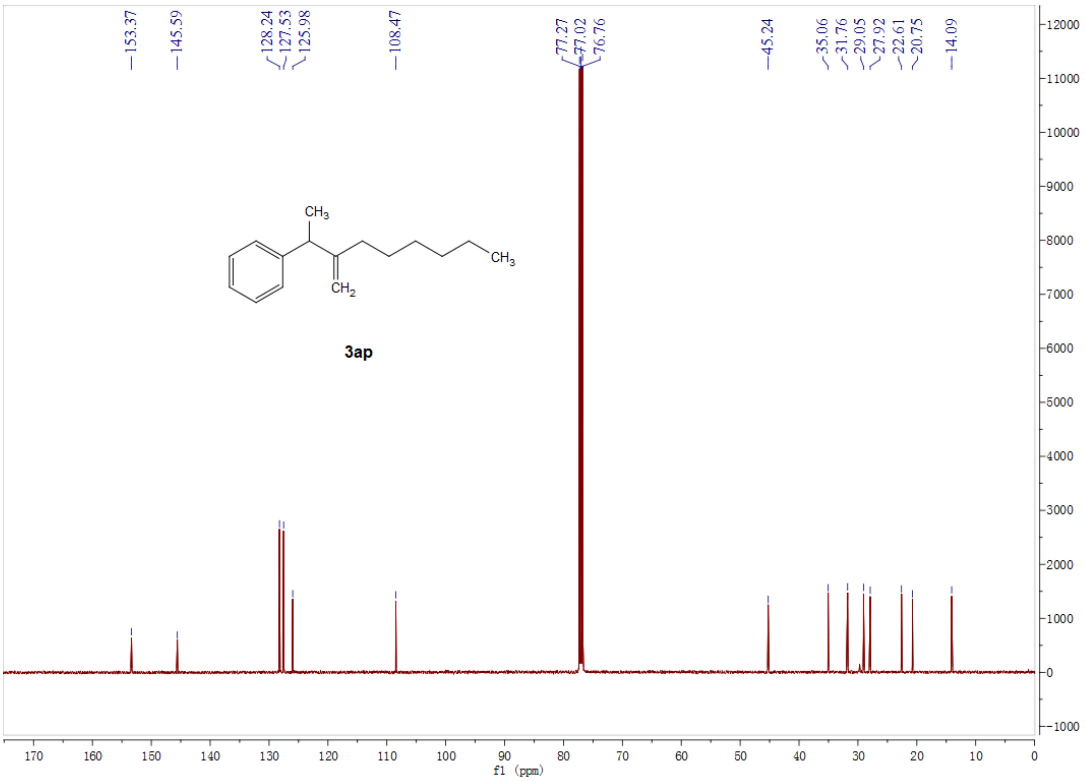
153.37
145.59
128.24 127.53 125.98
108.47
77.27 77.02 76.76
45.24
35.06 31.76 29.05 27.92 22.61 20.75
14.09
CH3
CH2
3ap
f1 (ppm)

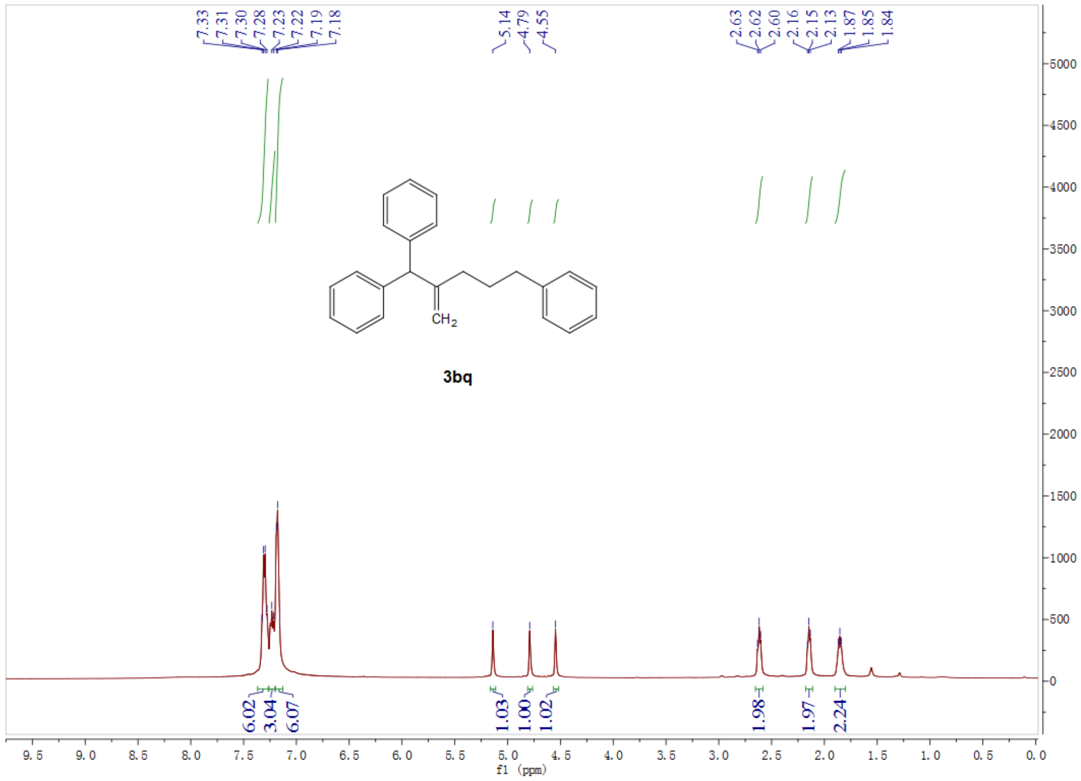
7.33
7.31
7.30
7.28
7.23
7.22
7.19
7.18
5.14
4.79
4.55
2.63
2.62
2.60
2.16
2.15
2.13
1.87
1.85
1.84
CH2
3bq
6.02
3.04
6.07
1.03
1.00
1.02
1.98
1.97
2.24
f1 (ppm)

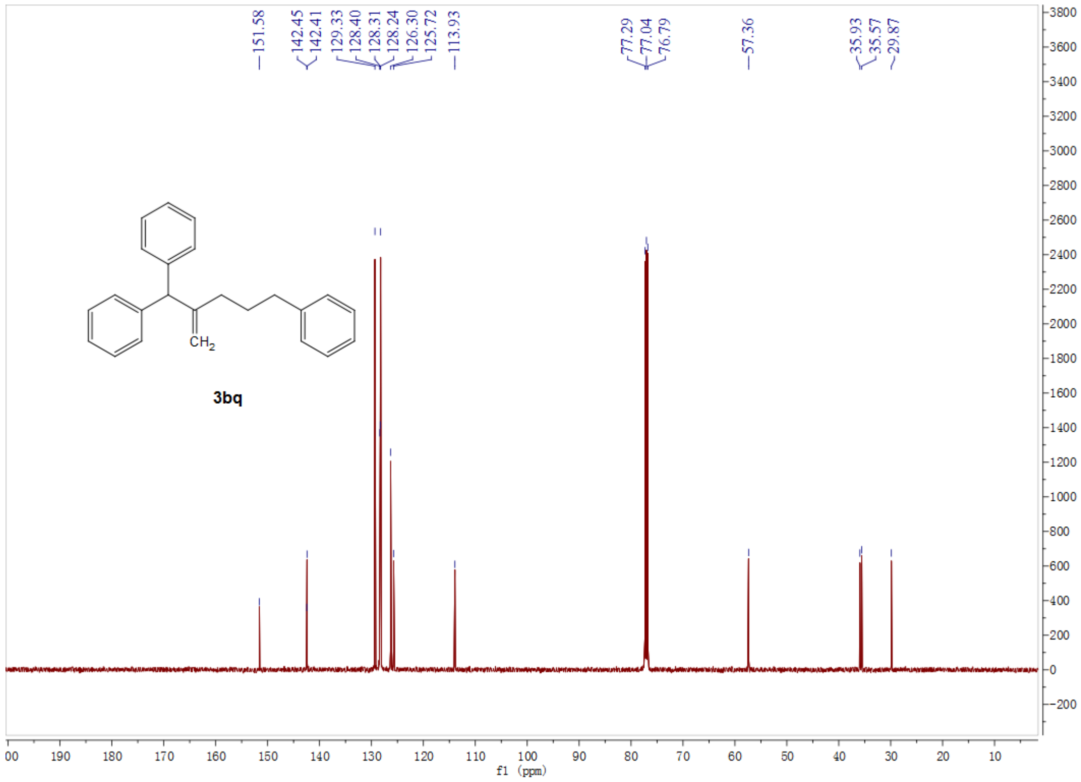
151.58
142.45
142.41
129.33
128.40
128.31
128.24
126.30
125.72
113.93
77.29
77.04
76.79
57.36
35.93
35.57
29.87
CH2
3bq
f1 (ppm)

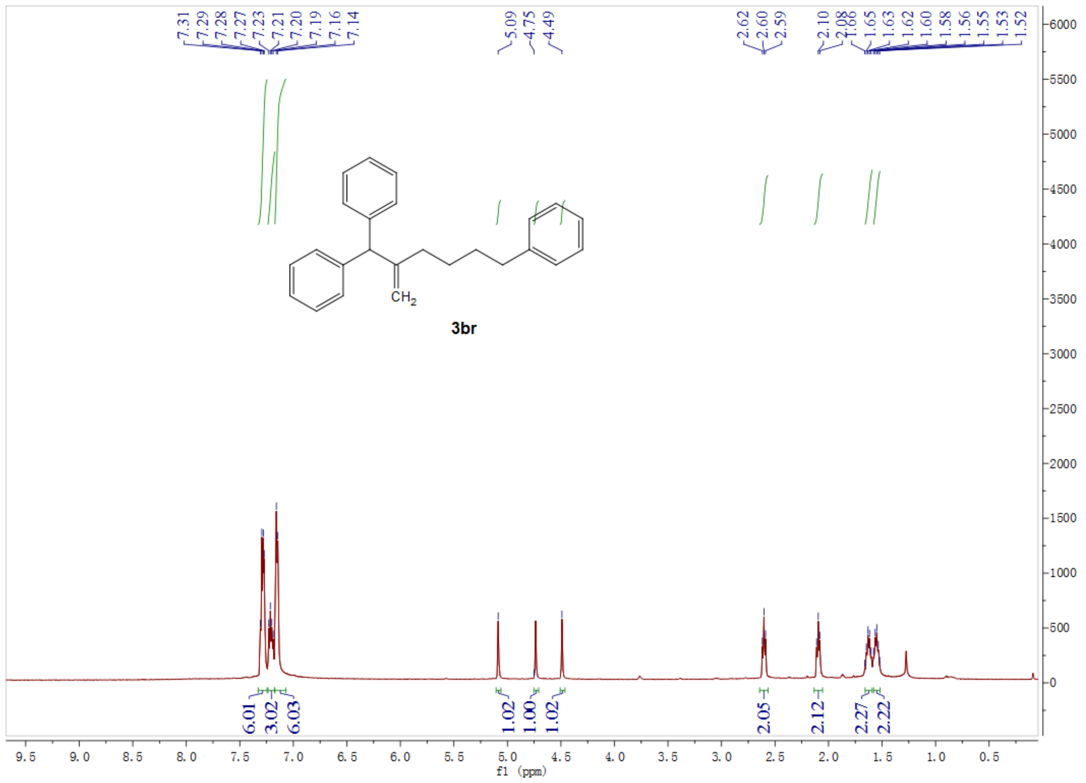

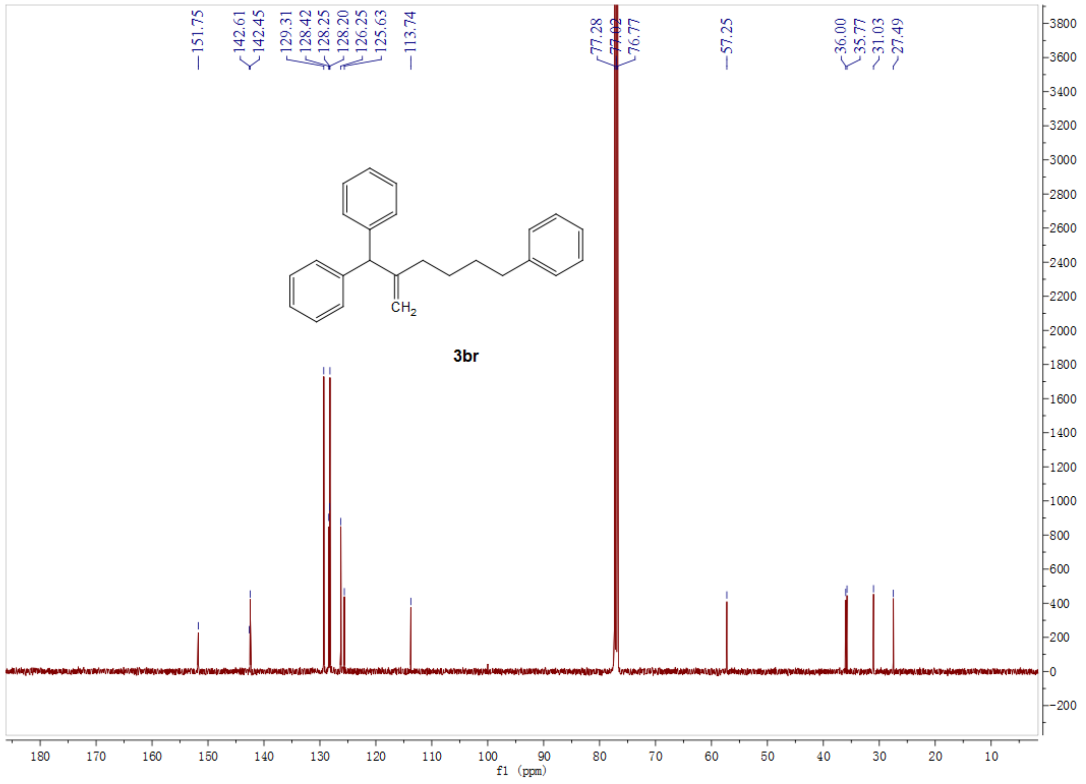

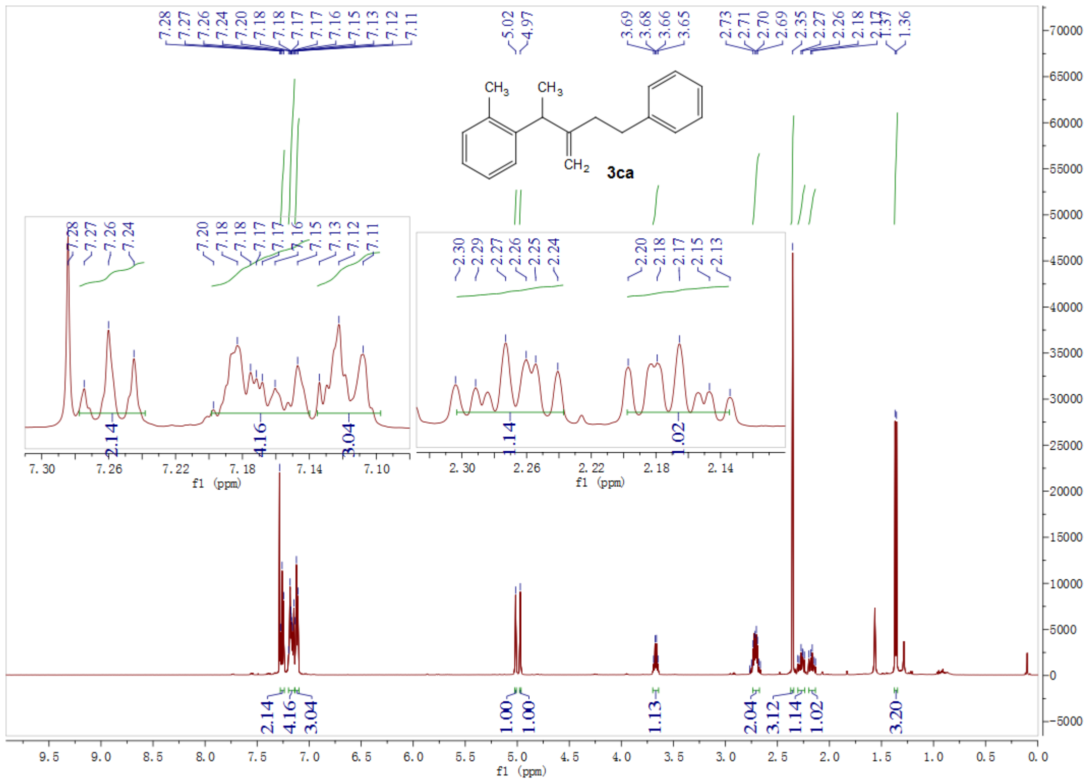
3ca

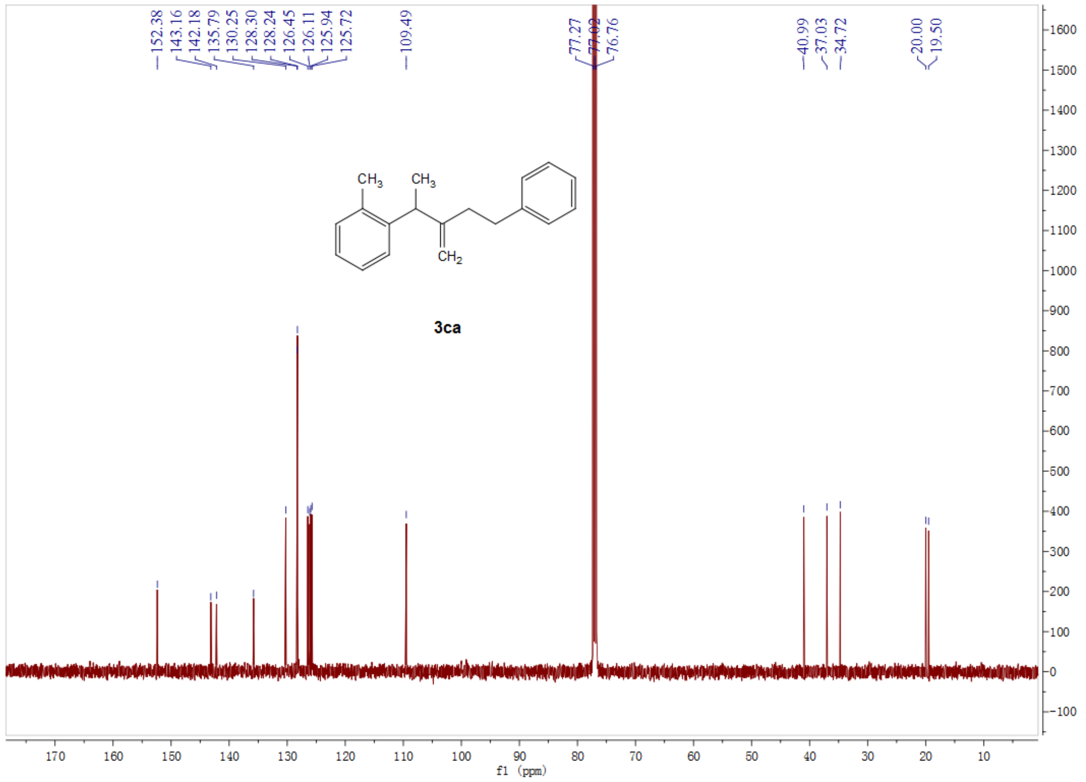
3ca

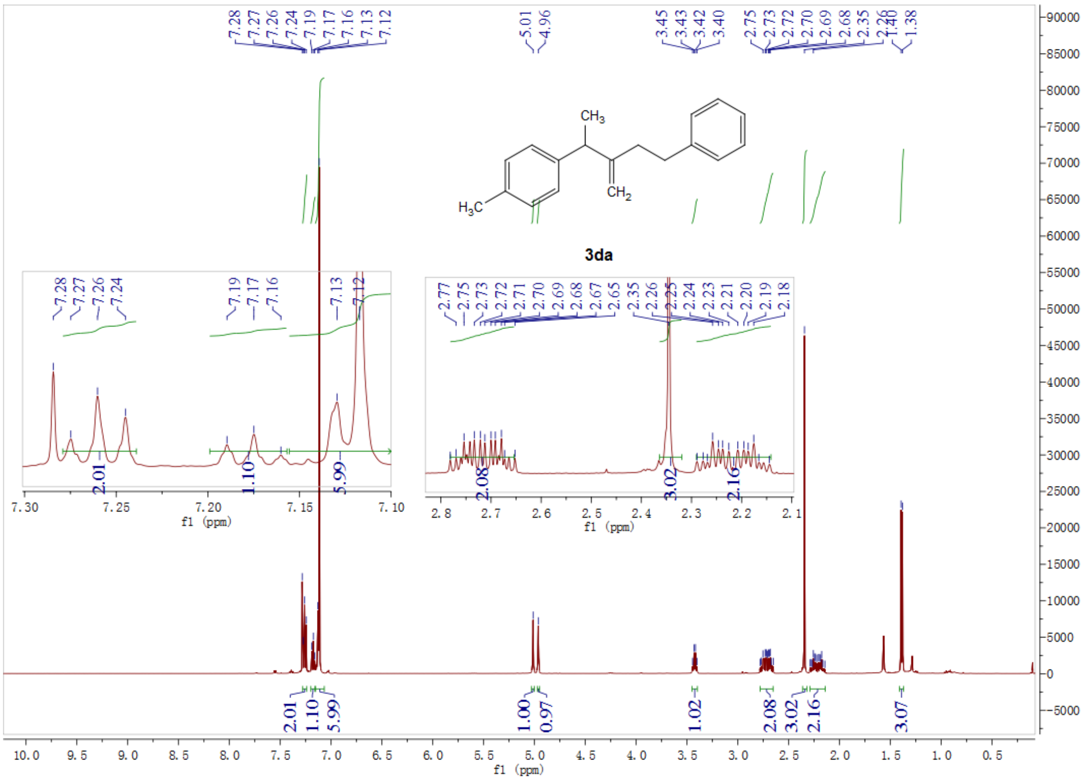
3da
f1 (ppm)

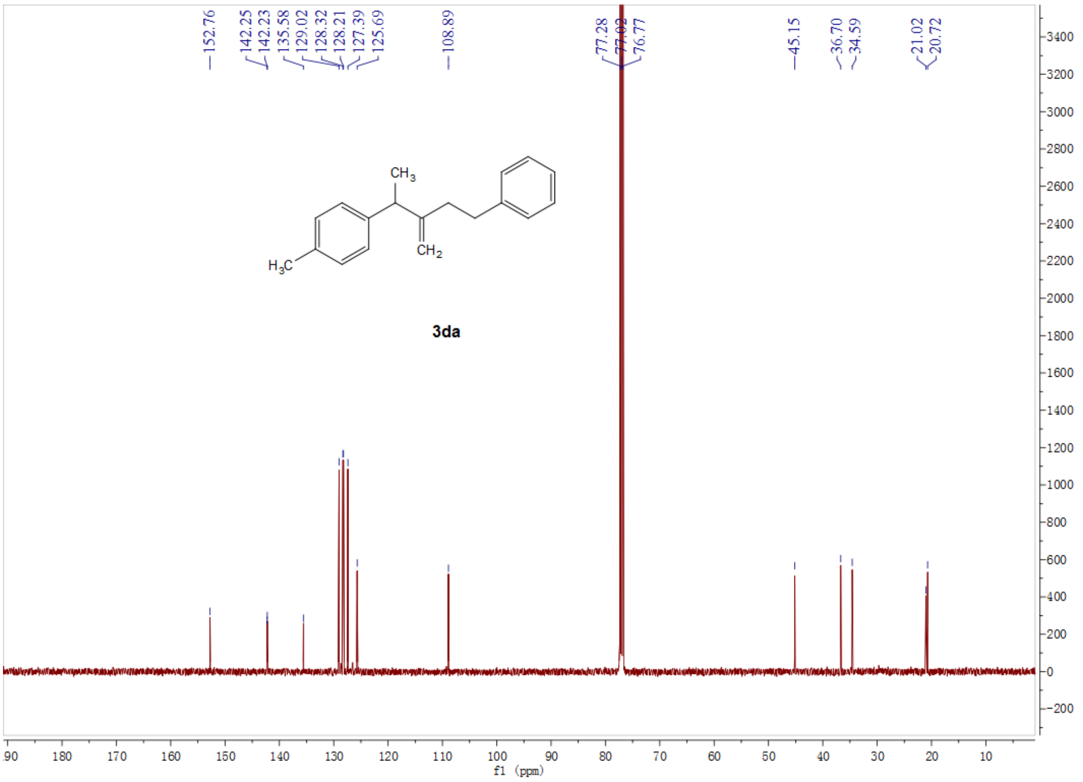
3da
f1 (ppm)

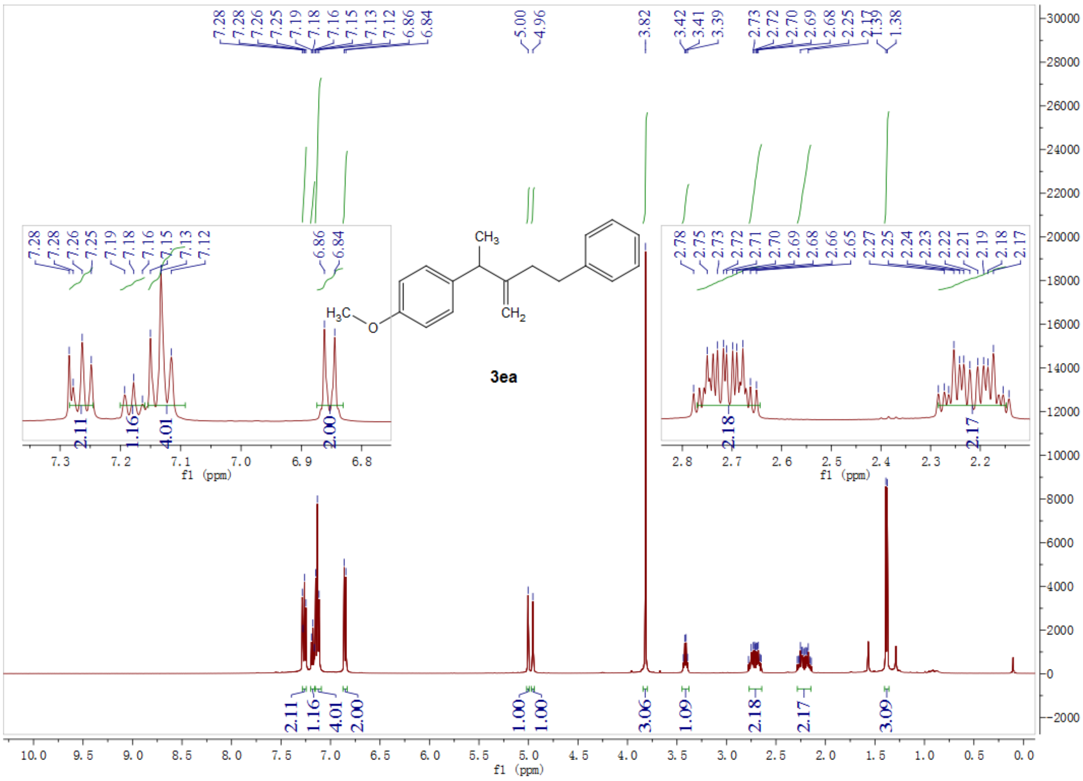
7.28 7.28 7.26 7.25 7.19 7.18 7.16 7.15 7.13 7.12 6.86 6.84
5.00 4.96
3.82
3.42 3.41 3.39
2.73 2.72 2.70 2.69 2.68 2.25 2.17 1.39 1.38
CH3
H3C
O
CH2
3ea
2.11 1.16 4.01 2.00 1.00 1.00 3.06 1.09 2.18 2.17 3.09
2.78 2.75 2.73 2.72 2.71 2.70 2.69 2.68 2.66 2.65 2.27 2.25 2.24 2.23 2.22 2.21 2.19 2.18 2.17
f1 (ppm)

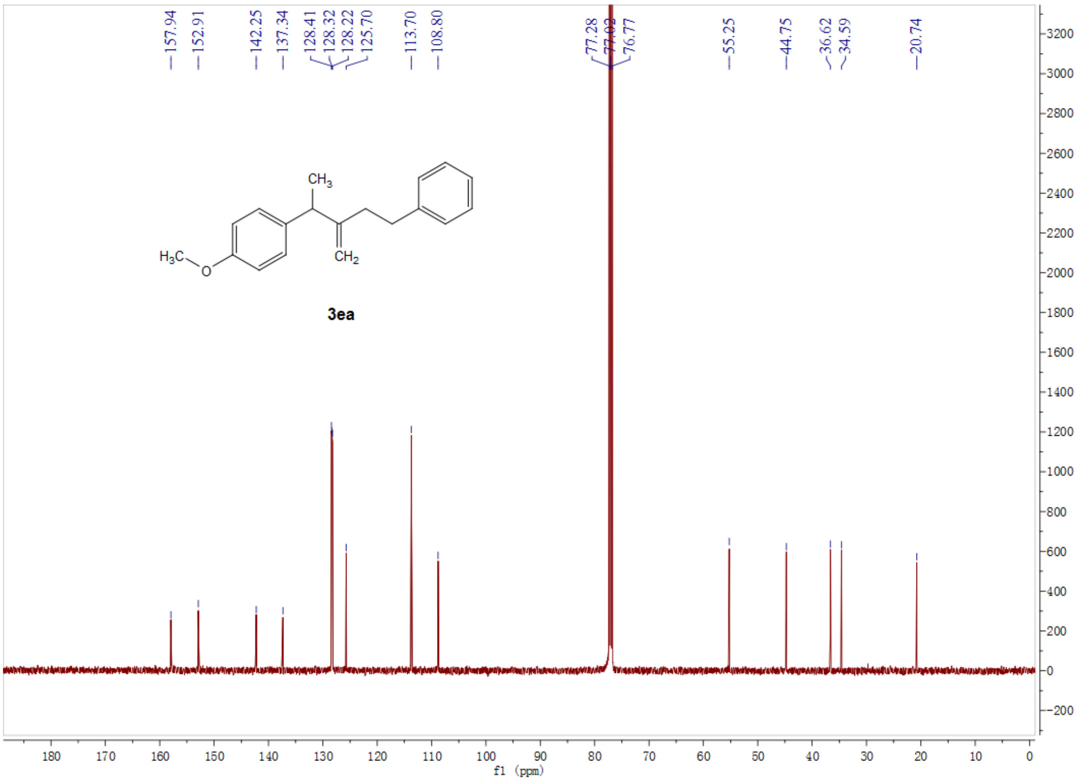
157.94 152.91 142.25 137.34 128.41 128.32 128.22 125.70 113.70 108.80
77.28 77.02 76.77
55.25 44.75 36.62 34.59 20.74
CH3
H3C
O
CH2
3ea
f1 (ppm)

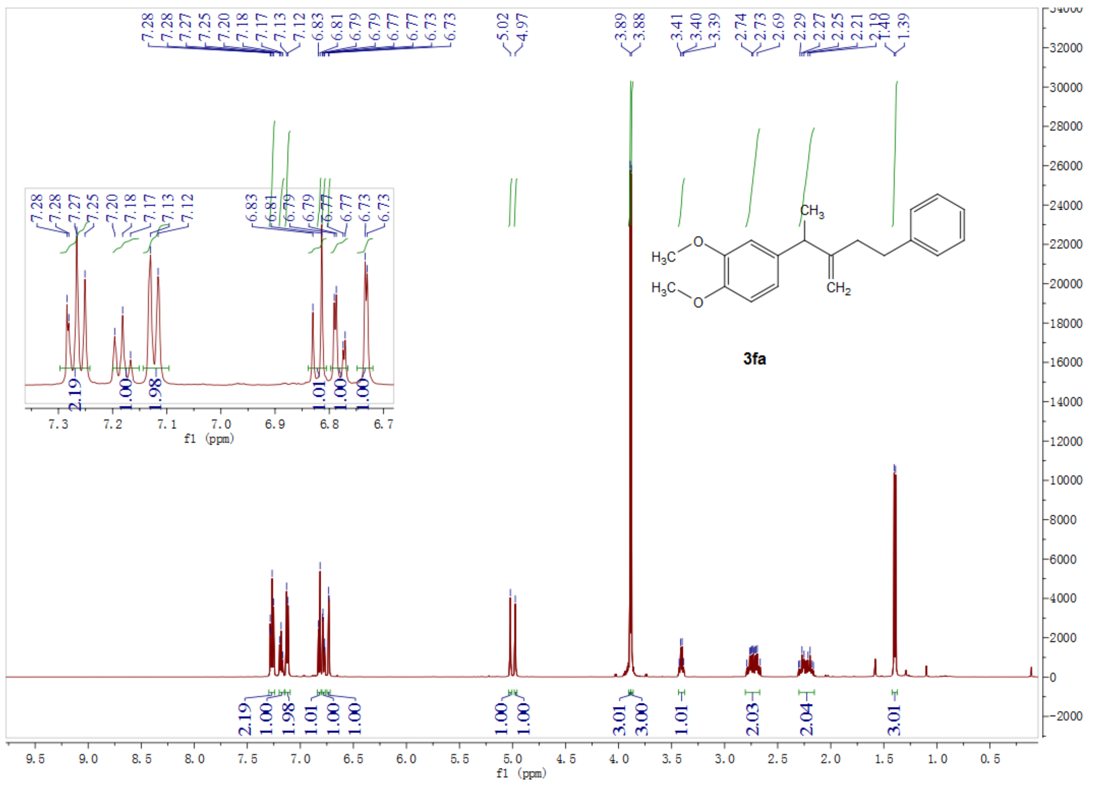

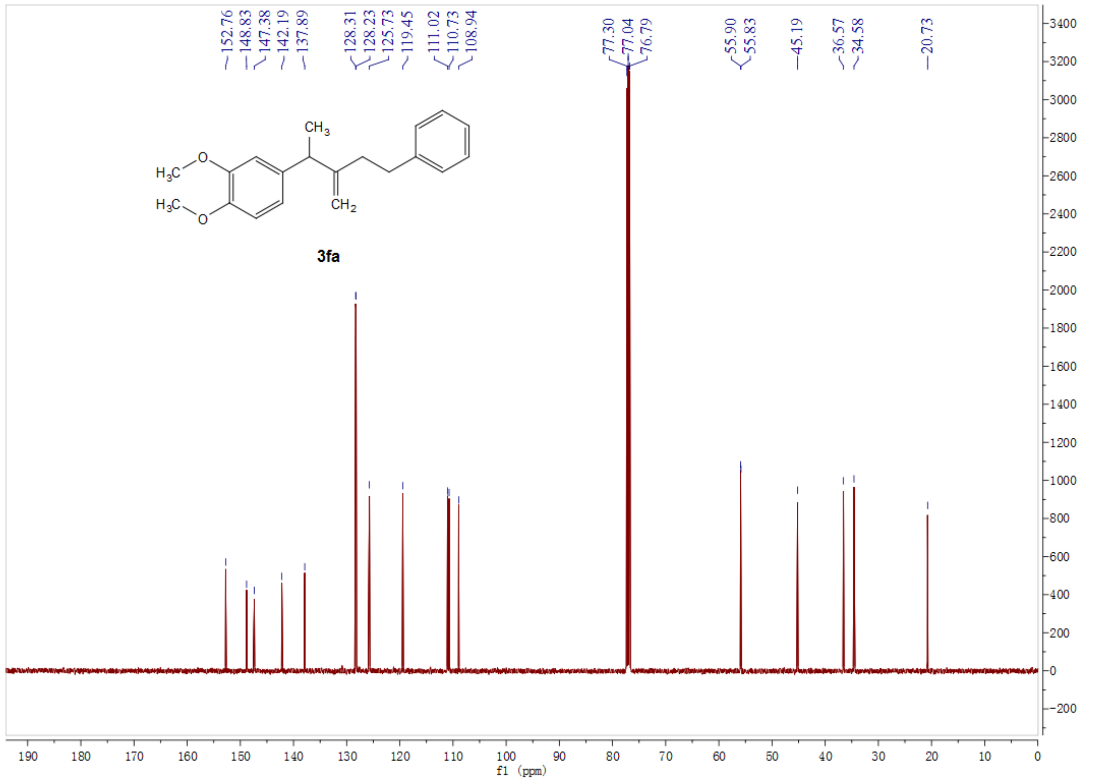

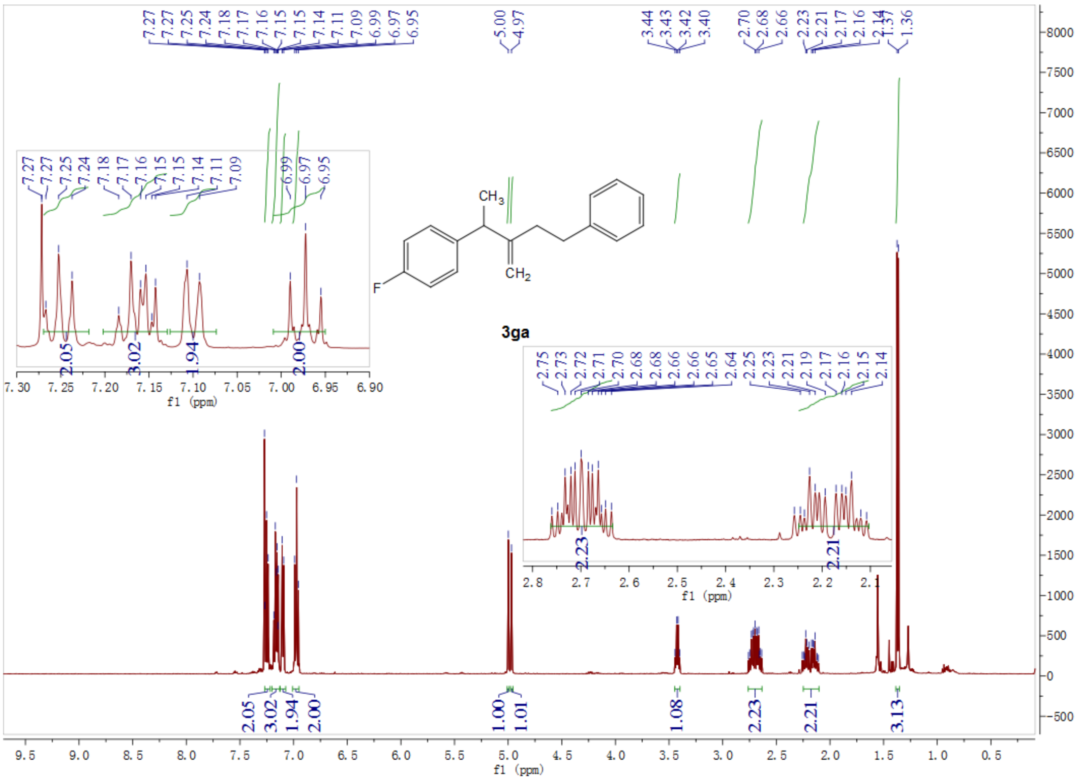
3ga
f1 (ppm)

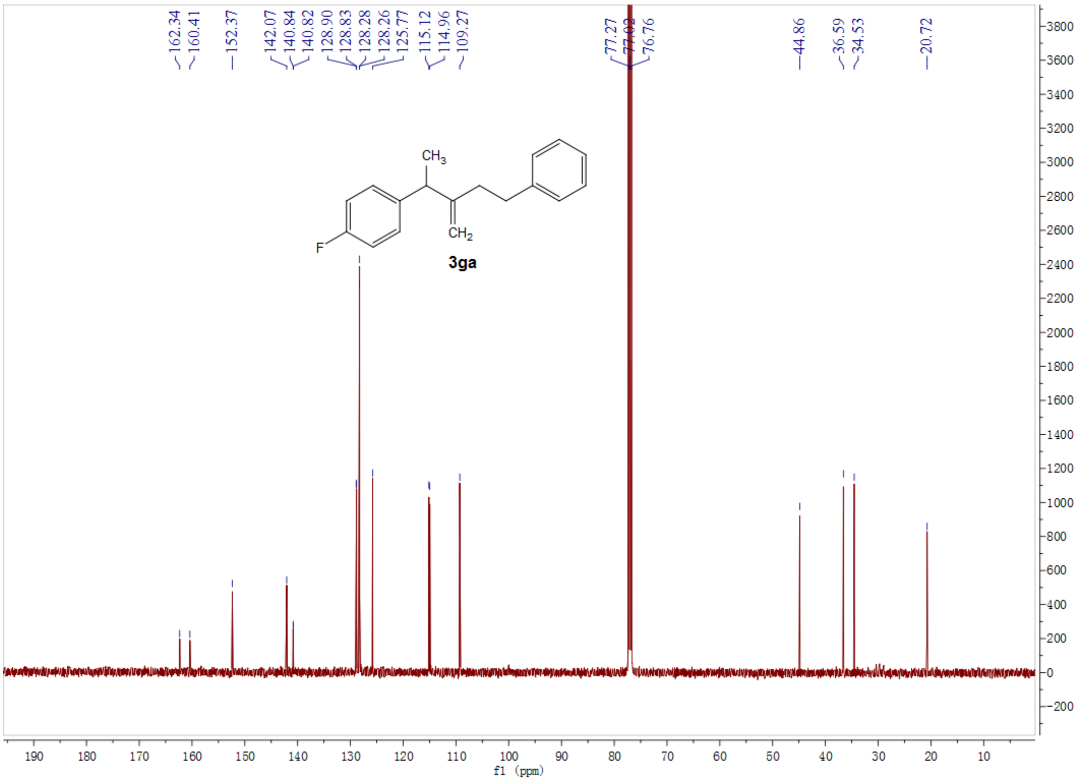
3ga
f1 (ppm)

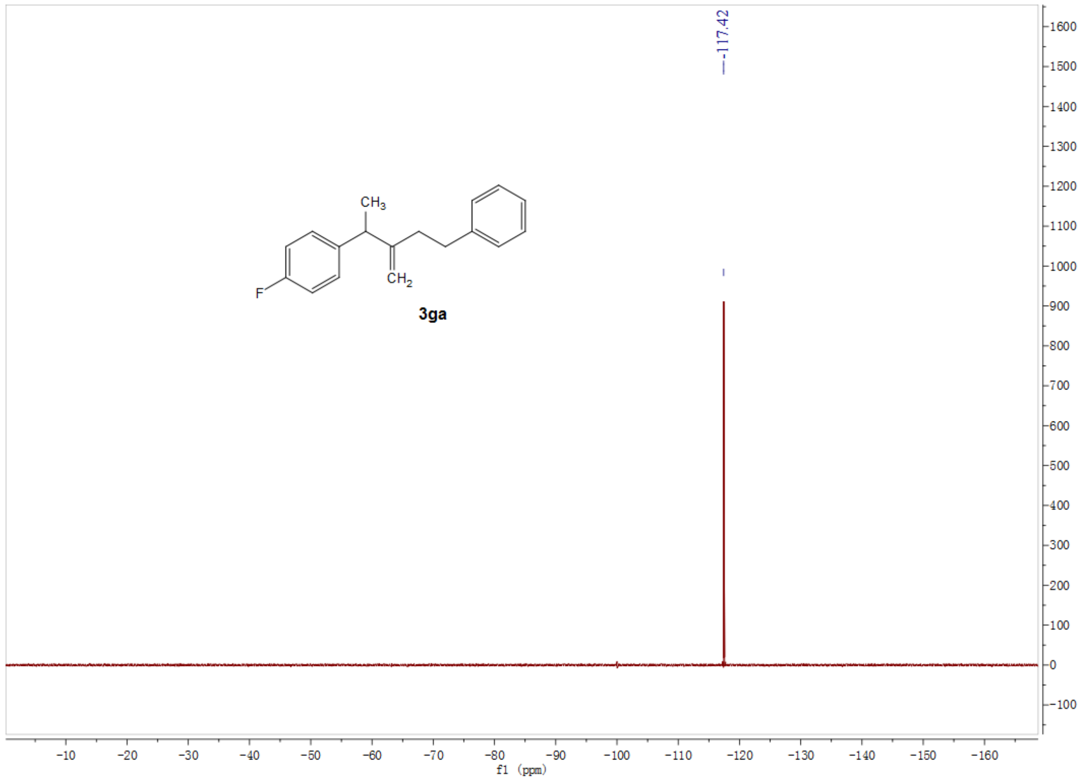

-117.42
CH3
CH2
F
3ga
f1 (ppm)

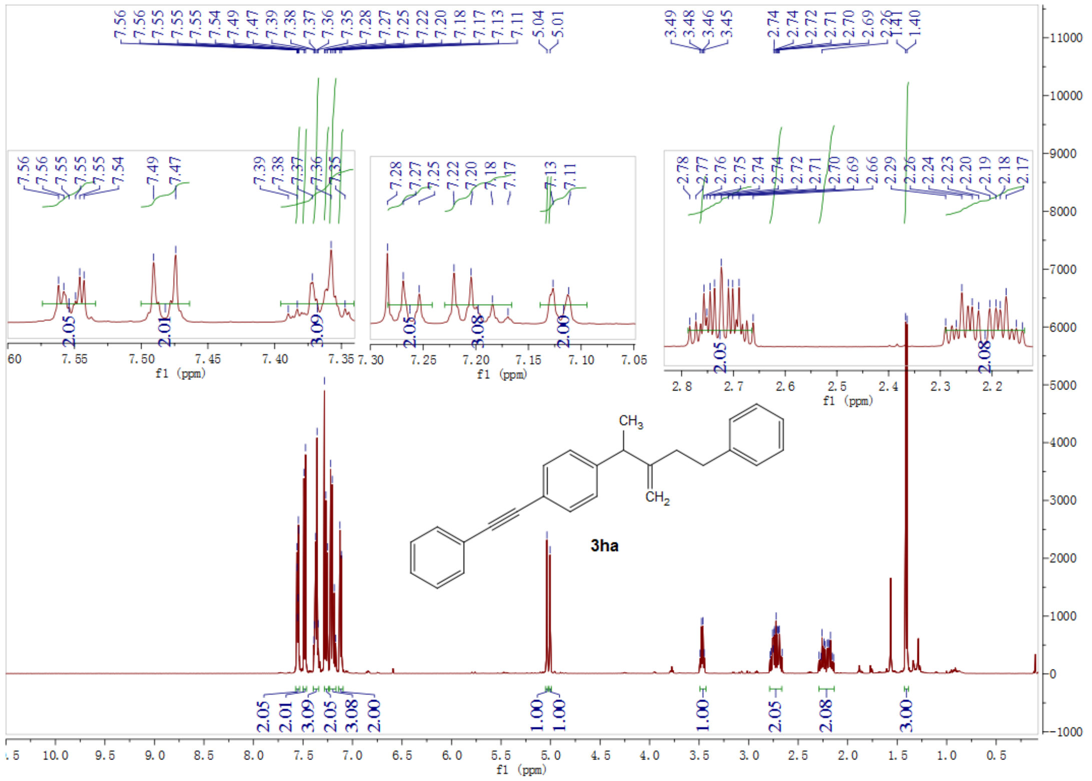
3ha

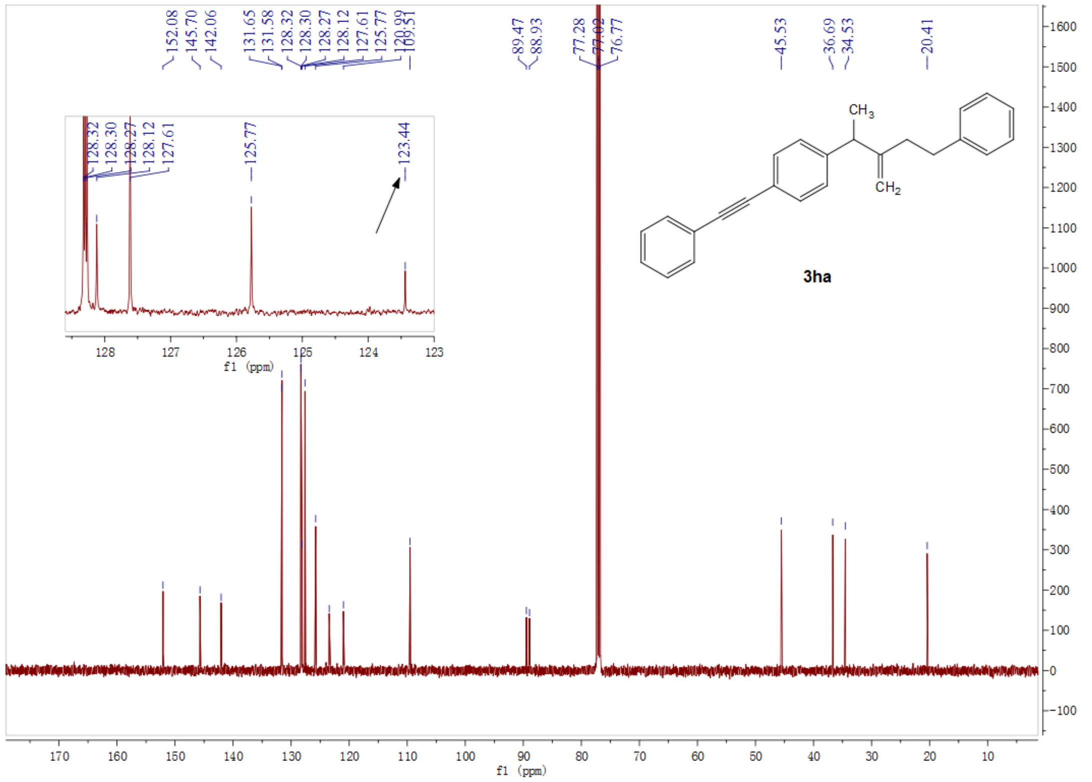
3ha

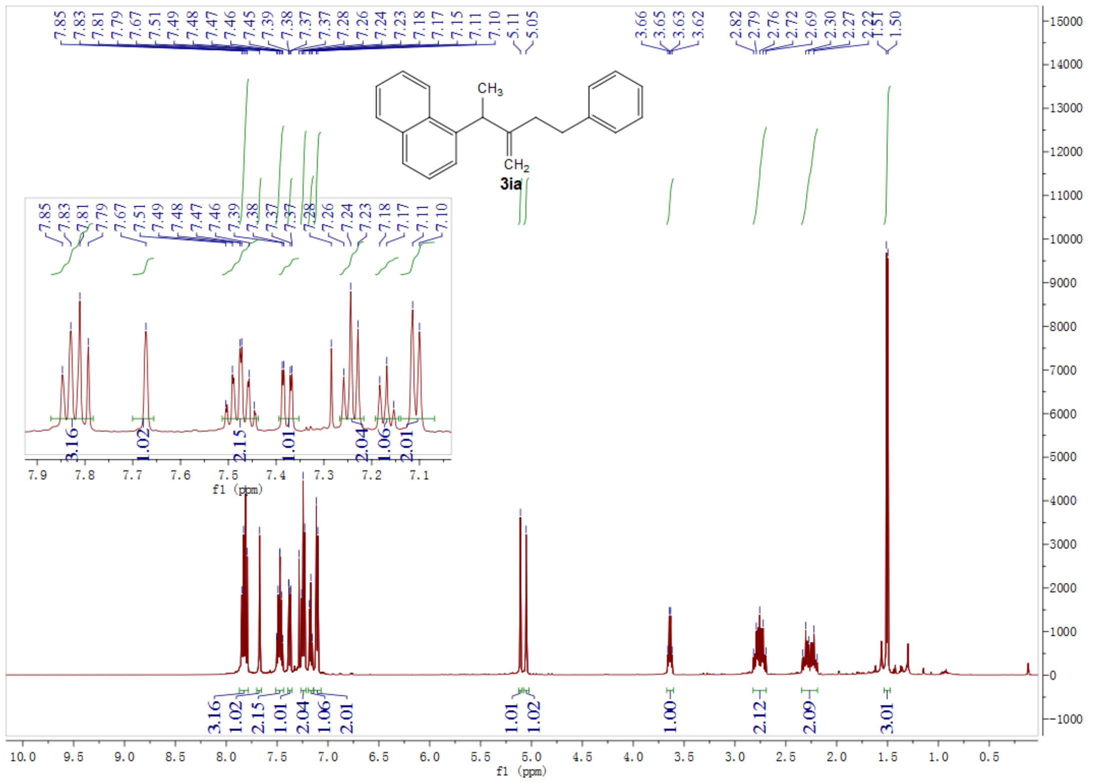

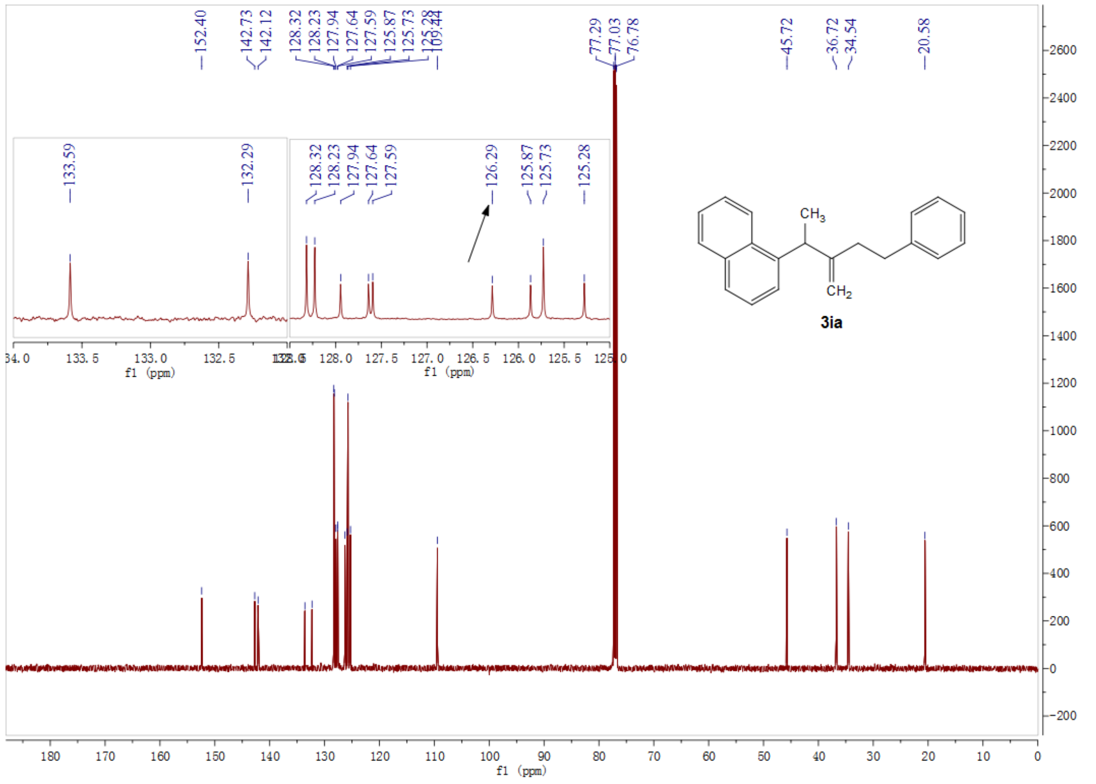

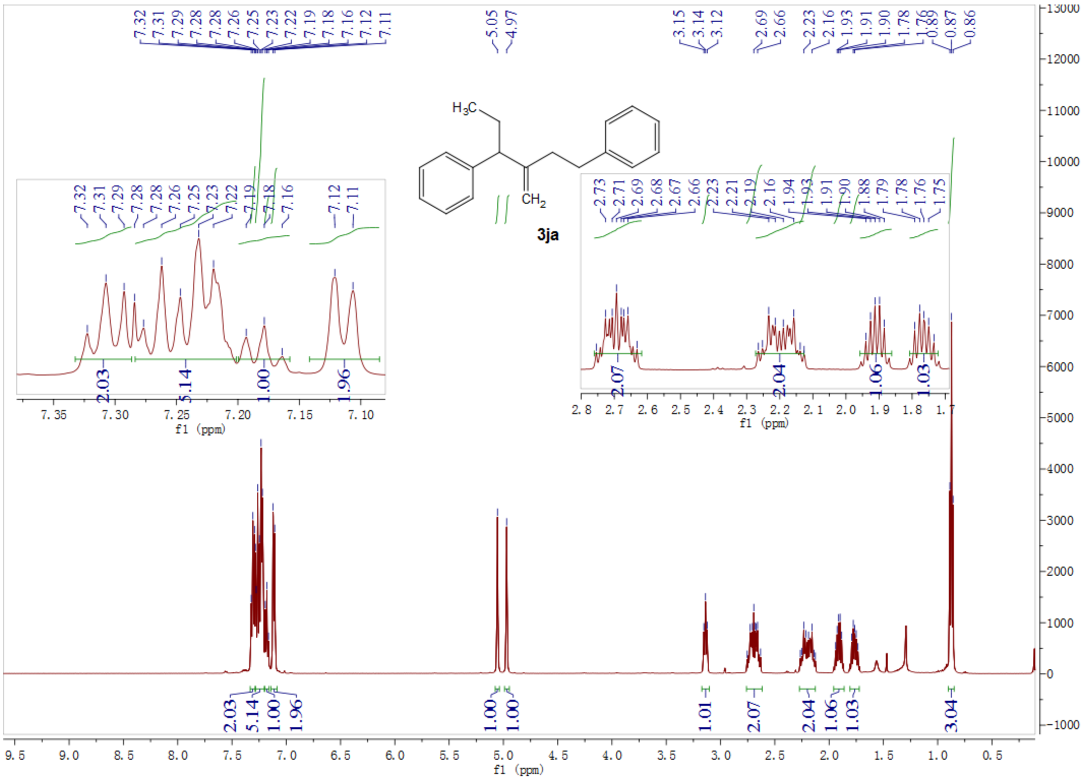

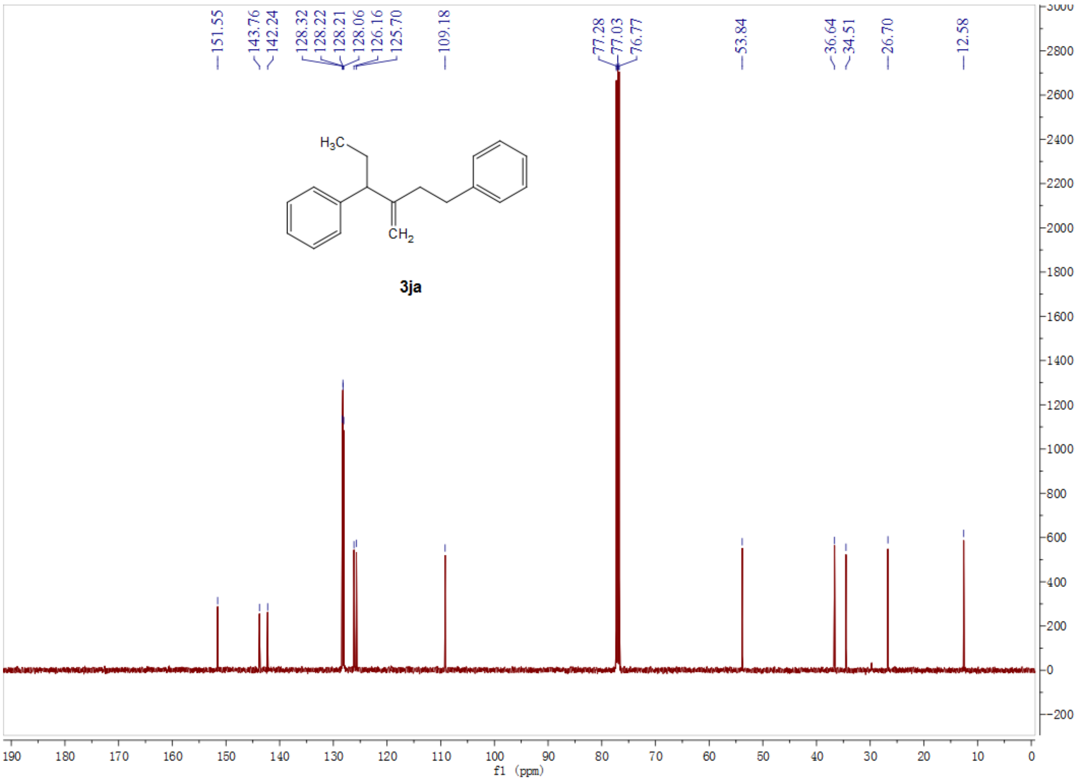

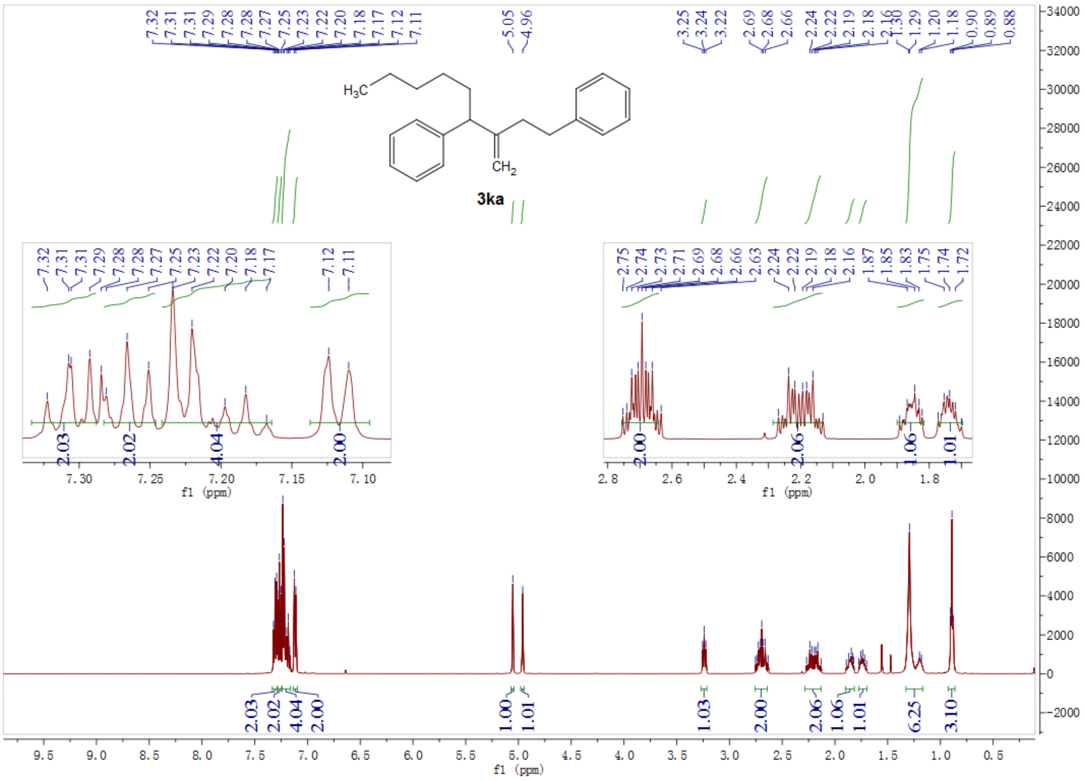
7.32 7.31 7.31 7.29 7.28 7.28 7.27 7.25 7.23 7.22 7.20 7.18 7.17 7.12 7.11
5.05 4.96
3.25 3.24 3.22 2.69 2.68 2.66 2.24 2.22 2.19 2.18 2.16 1.30 1.29 1.20 1.18 0.90 0.89 0.88
H3C
CH2
3ka
2.03 2.02 4.04 2.00 1.00 1.01 1.03 2.00 2.06 1.06 1.01 6.25 3.10
2.75 2.74 2.73 2.71 2.69 2.68 2.66 2.63 2.24 2.22 2.19 2.18 2.16 1.87 1.85 1.83 1.75 1.74 1.72
f1 (ppm)

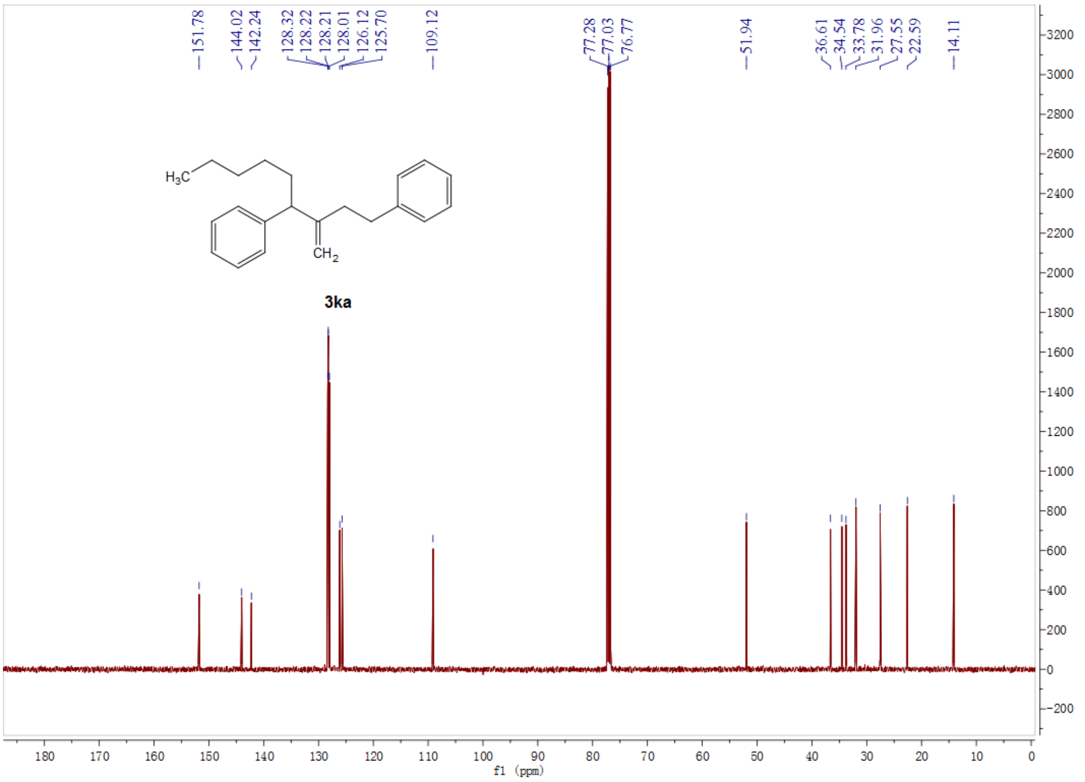
151.78 144.02 142.24 128.32 128.22 128.21 128.01 126.12 125.70 109.12 77.28 77.03 76.77 51.94 36.61 34.54 33.78 31.96 27.55 22.59 14.11
H3C
CH2
3ka
f1 (ppm)

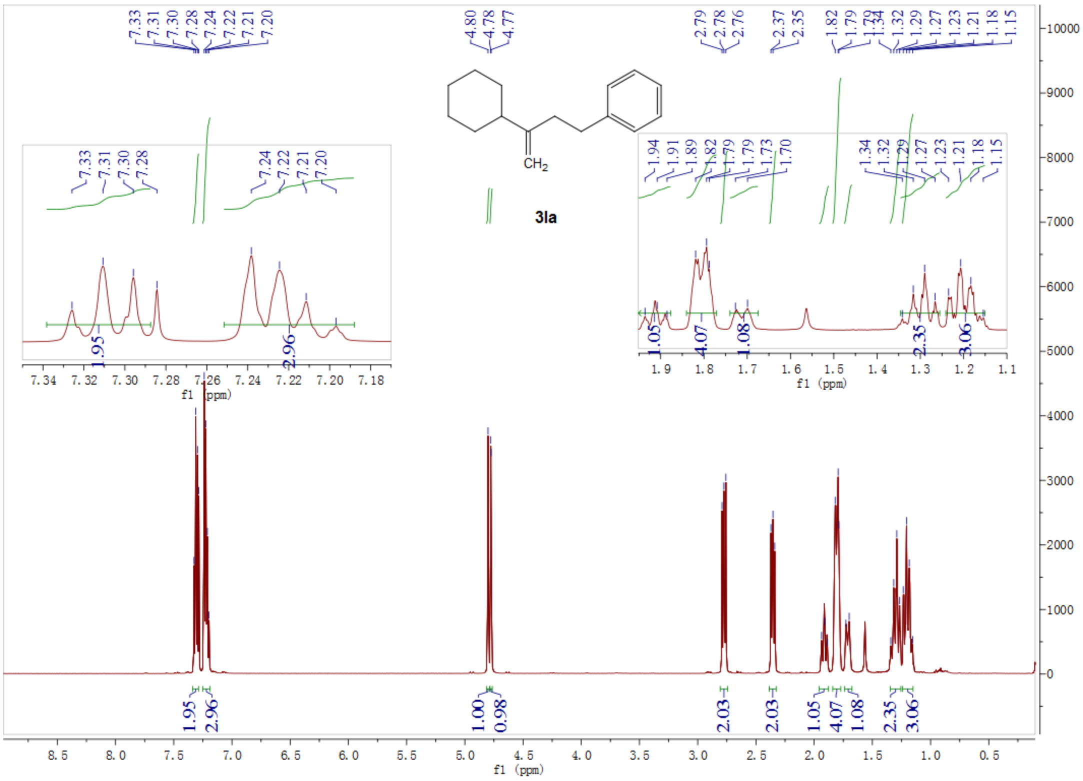

3la
CH2
f1 (ppm)

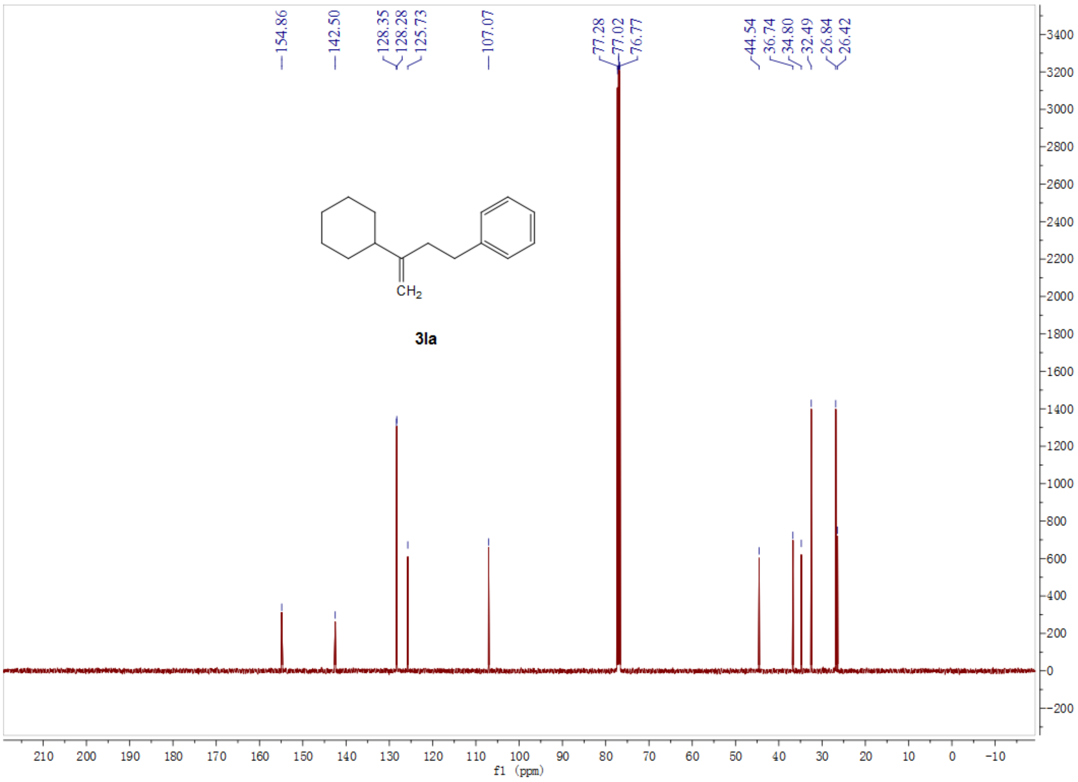

3la
CH2
f1 (ppm)

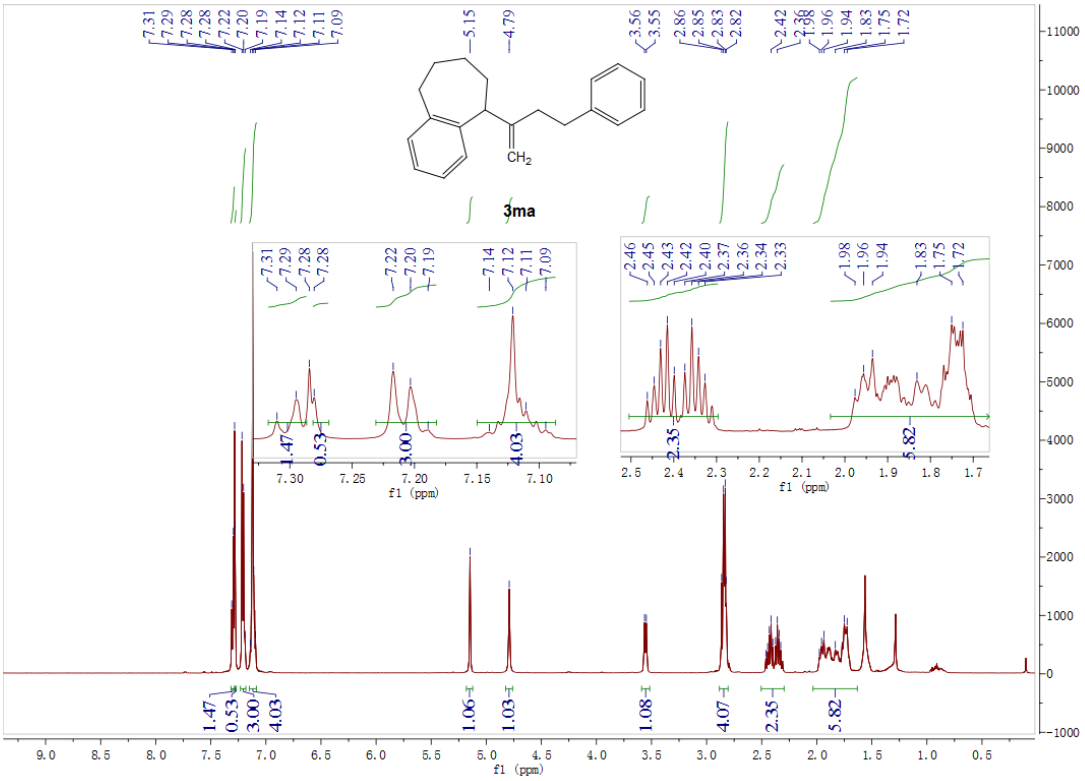
7.31 7.29 7.28 7.28 7.22 7.20 7.19 7.14 7.12 7.11 7.09
5.15
4.79
3.56 3.55
2.86 2.85 2.83 2.82
2.42 2.36 1.98 1.96 1.94 1.83 1.75 1.72
CH2
3ma
1.47 0.53 3.00 4.03 1.06 1.03 1.08 4.07 2.35 5.82
f1 (ppm)

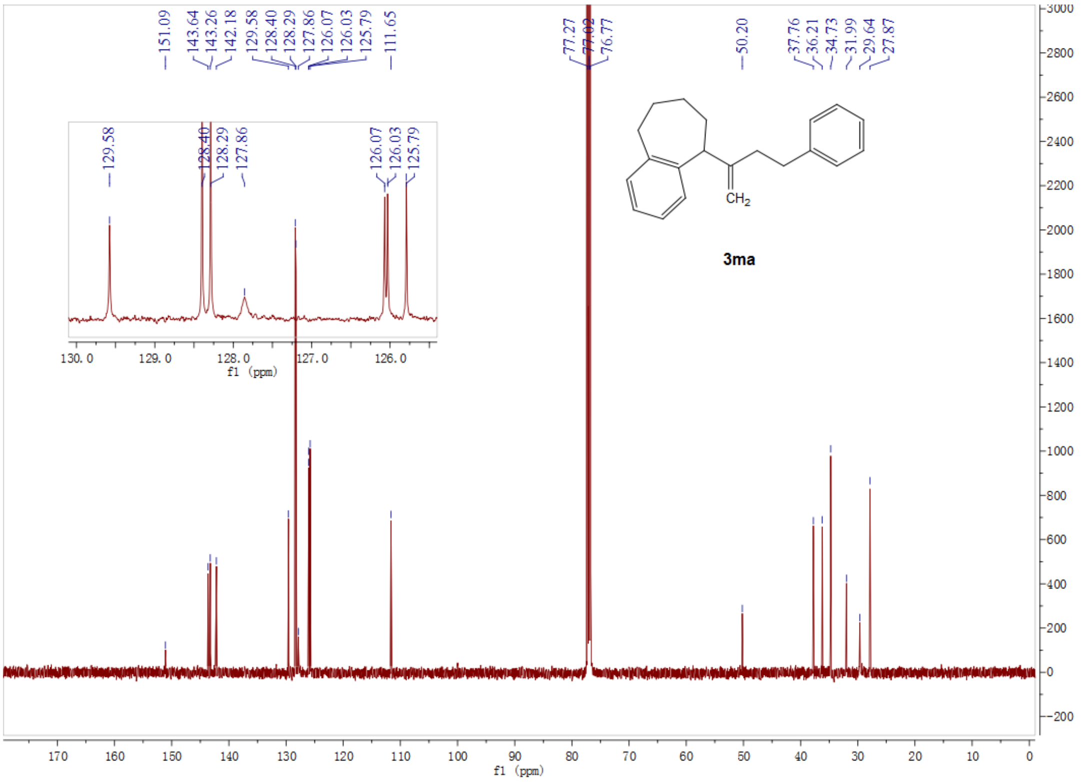
151.09
143.64 143.26 142.18 129.58 128.40 128.29 127.86 126.07 126.03 125.79
111.65
77.27 77.02 76.77
50.20
37.76 36.21 34.73 31.99 29.64 27.87
CH2
3ma
f1 (ppm)

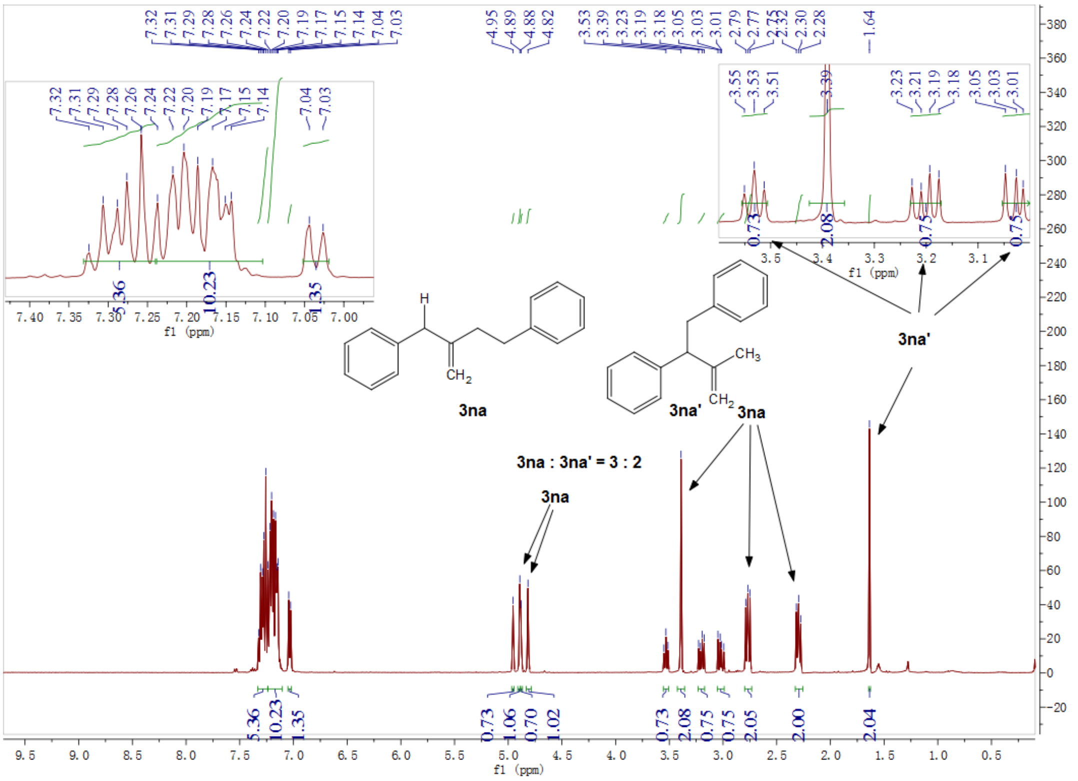
3na
3na'
3na : 3na' = 3 : 2
f1 (ppm)

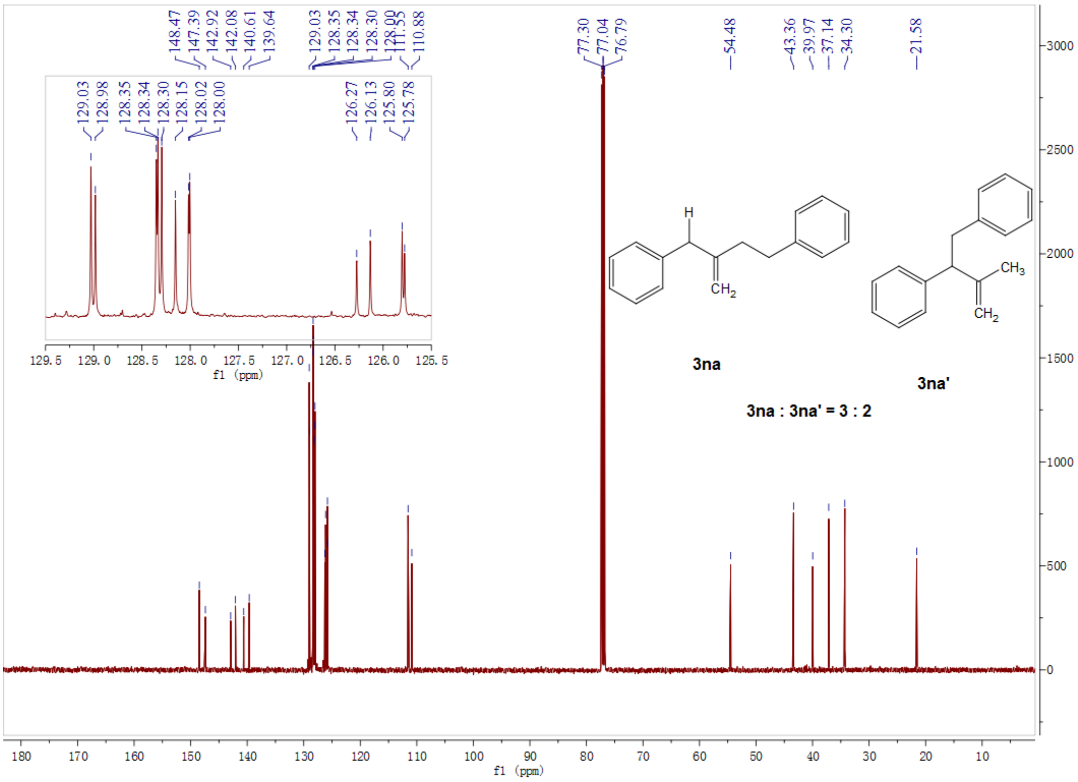
3na
3na'
3na : 3na' = 3 : 2
f1 (ppm)
